# Rare, convergent antibodies targeting the stem helix broadly neutralize diverse betacoronaviruses

**DOI:** 10.1016/j.chom.2023.05.016

**Published:** 2023-06-14

**Authors:** Cherrelle Dacon, Linghang Peng, Ting-Hui Lin, Courtney Tucker, Chang-Chun D. Lee, Yu Cong, Lingshu Wang, Lauren Purser, Andrew J.R. Cooper, Jazmean K. Williams, Chul-Woo Pyo, Meng Yuan, Ivan Kosik, Zhe Hu, Ming Zhao, Divya Mohan, Mary Peterson, Jeff Skinner, Saurabh Dixit, Erin Kollins, Louis Huzella, Donna Perry, Russell Byrum, Sanae Lembirik, Michael Murphy, Yi Zhang, Eun Sung Yang, Man Chen, Kwanyee Leung, Rona S. Weinberg, Amarendra Pegu, Daniel E. Geraghty, Edgar Davidson, Benjamin J. Doranz, Iyadh Douagi, Susan Moir, Jonathan W. Yewdell, Connie Schmaljohn, Peter D. Crompton, John R. Mascola, Michael R. Holbrook, David Nemazee, Ian A. Wilson, Joshua Tan

(Cell Host & Microbe *31*, 97–111.e1–e12; January 11, 2023)

In Figure 6A, the authors discovered after publication that the neutralization NT_50_ values of two similar IGHV1-46/IGKV3-20 mAbs (COV30-14 and COV72-37) for several SARS-CoV-2 variants were switched due to a copying error. This correction does not impact the conclusions of the original paper. The affected NT_50_ values of the two mAbs only differed <2 fold. The authors regret the inconvenience caused by this error, which has been corrected in the original article online.

**Figure 6A:**
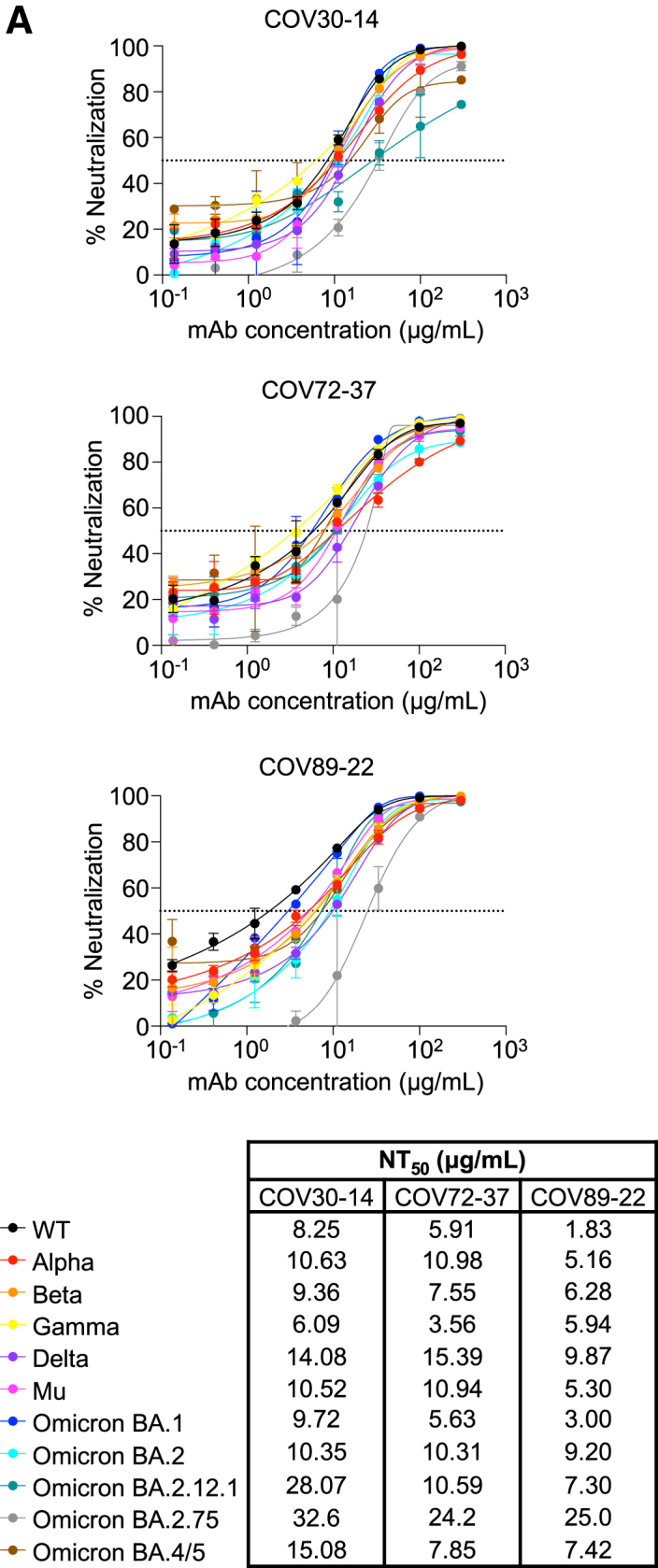
COV30-14, COV72-37, and COV89-22 neutralize SARS-CoV-2 variants of concern and inhibit SARS-CoV-2 spike-mediated fusion (corrected)

**Figure 6A:**
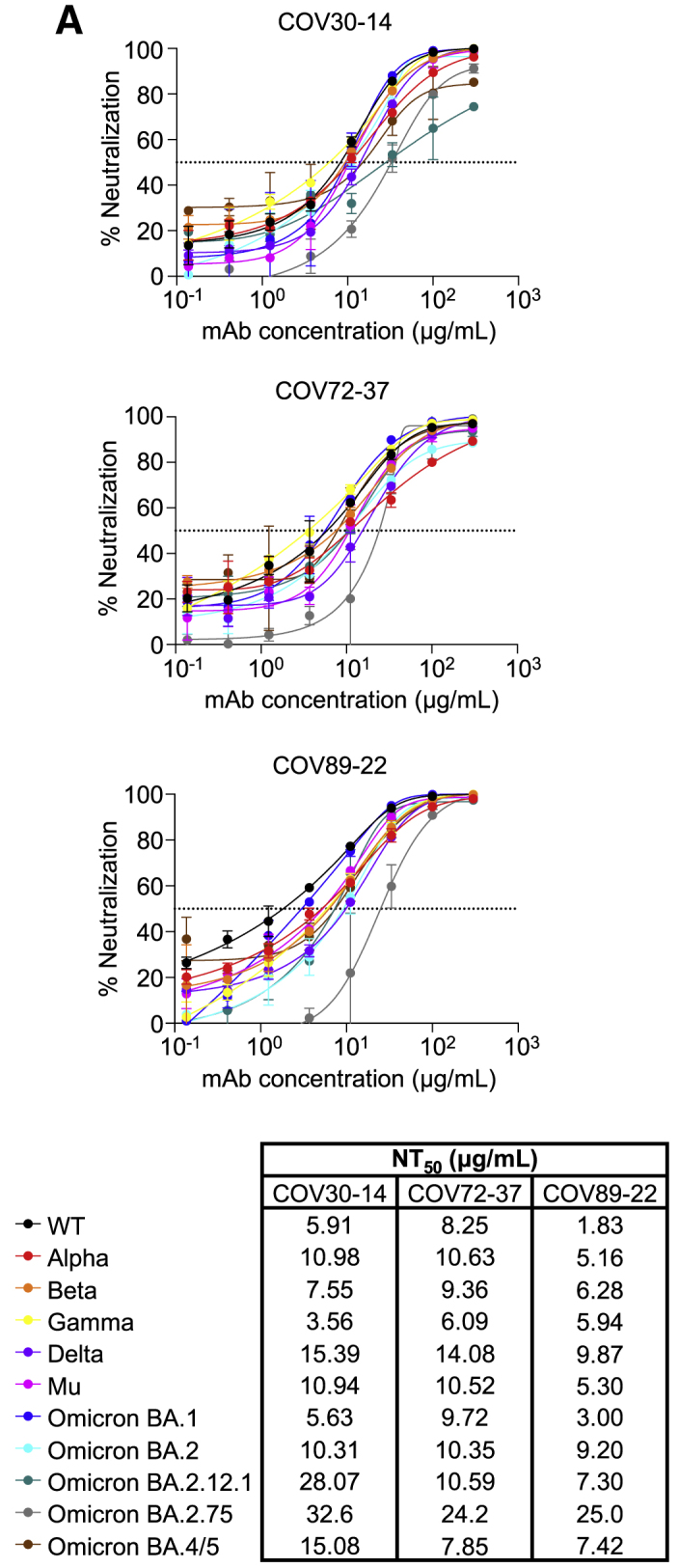
COV30-14, COV72-37, and COV89-22 neutralize SARS-CoV-2 variants of concern and inhibit SARS-CoV-2 spike-mediated fusion (original)

