# Supplementary material for: Rare, convergent antibodies targeting the stem helix broadly neutralize diverse betacoronaviruses
Source: Cell Host Microbe. 2023 Jan 11;31(1):97–111.e12. doi: 10.1016/j.chom.2022.10.010 (PMC9639329; doi:10.1016/j.chom.2022.10.010)
Supplement: Document S1. Figures S1–S7, Tables S1–S3, and supplemental references [file mmc1.pdf]

Cell Host & Microbe, Volume *31*

Supplemental information

Rare, convergent antibodies targeting the stem helix broadly neutralize diverse betacoronaviruses

Cherrelle Dacon, Linghang Peng, Ting-Hui Lin, Courtney Tucker, Chang-Chun D. Lee, Yu Cong, Lingshu Wang, Lauren Purser, Andrew J.R. Cooper, Jazmean K. Williams, Chul-Woo Pyo, Meng Yuan, Ivan Kosik, Zhe Hu, Ming Zhao, Divya Mohan, Mary Peterson, Jeff Skinner, Saurabh Dixit, Erin Kollins, Louis Huzella, Donna Perry, Russell Byrum, Sanae Lembirik, Michael Murphy, Yi Zhang, Eun Sung Yang, Man Chen, Kwanyee Leung, Rona S. Weinberg, Amarendra Pegu, Daniel E. Geraghty, Edgar Davidson, Benjamin J. Doranz, Iyadh Douagi, Susan Moir, Jonathan W. Yewdell, Connie Schmaljohn, Peter D. Crompton, John R. Mascola, Michael R. Holbrook, David Nemazee, Ian A. Wilson, and Joshua Tan

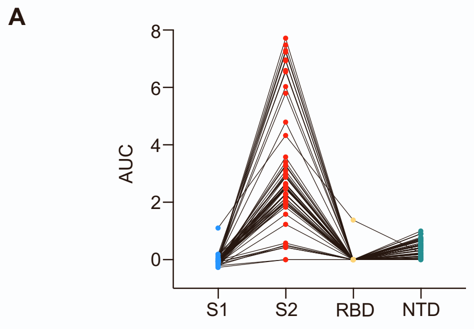

Figure S1. Broadly reactive mAbs against betacoronaviruses target two distinct epitopes on the SARS-CoV-2 S2 subunit, related to Figure 2.

(A) Area under the curve (AUC) analyses for titration of mAb binding to the S1 subunit, S2 subunit, receptor binding domain (RBD), and the N-terminal domain (NTD). AUC values for each antigen are shown after subtraction with values for the negative control antigen CD4.

(B) Epitope binning of broadly reactive antibodies. The S2 stem helix-targeting mAb S2P6 as well as fusion peptide-binding mAbs COV44-62 and COV44-79 were added as controls. Red boxes indicate competing antibody pairs, green boxes indicate non-competing antibody pairs and hashed filling indicates self-competition. Signals generated by both the ligand and analyte condition for each mAb were used to compute bins except for COV44-37, COV44-56, COV49-51, COV72-37 and COV77-02, which were computed as analytes only due to low signal in the ligand condition. These low signals may be a result of poor conjugation to the chip or acid sensitivity resulting in denaturation during the regeneration steps.

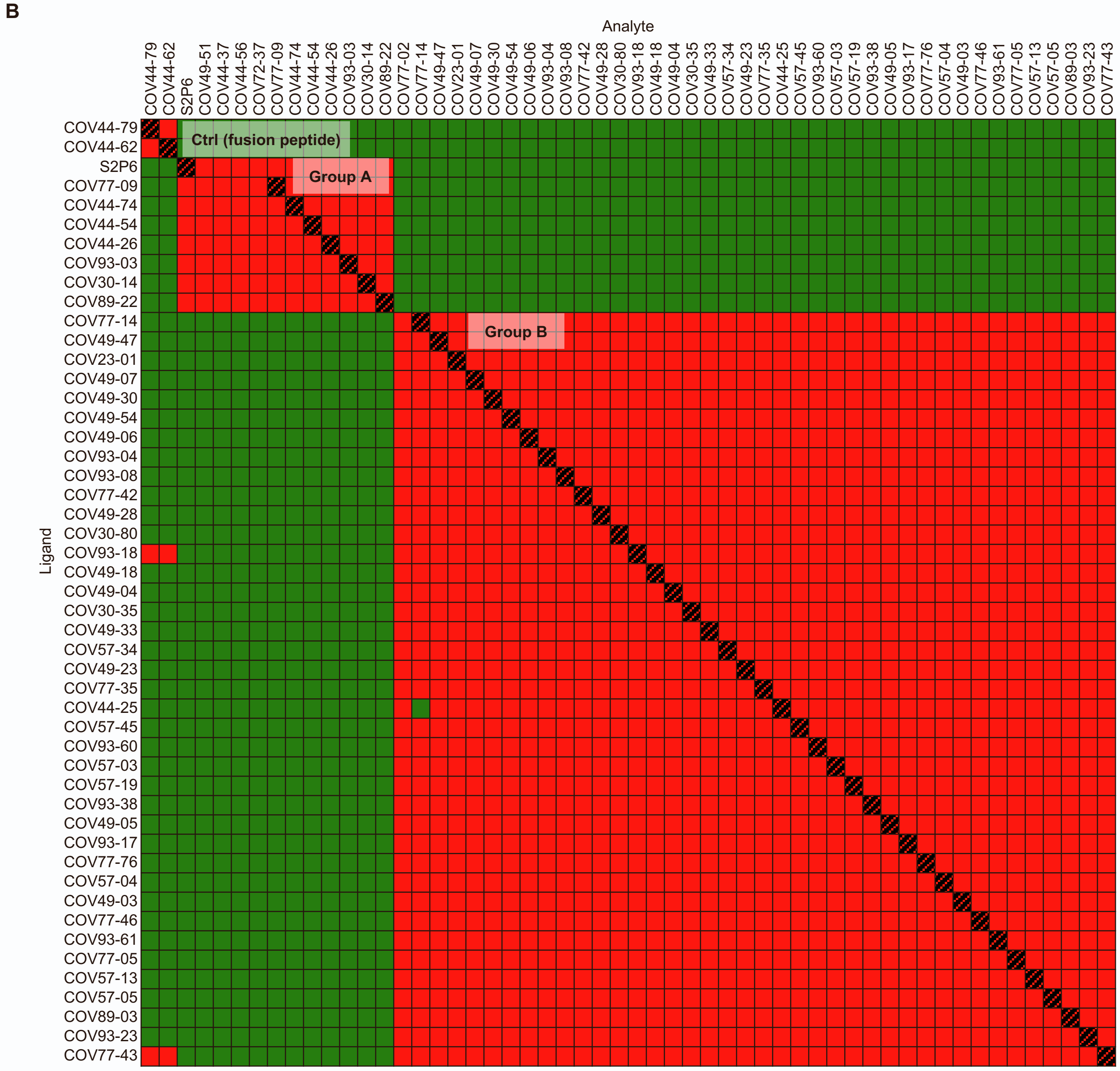

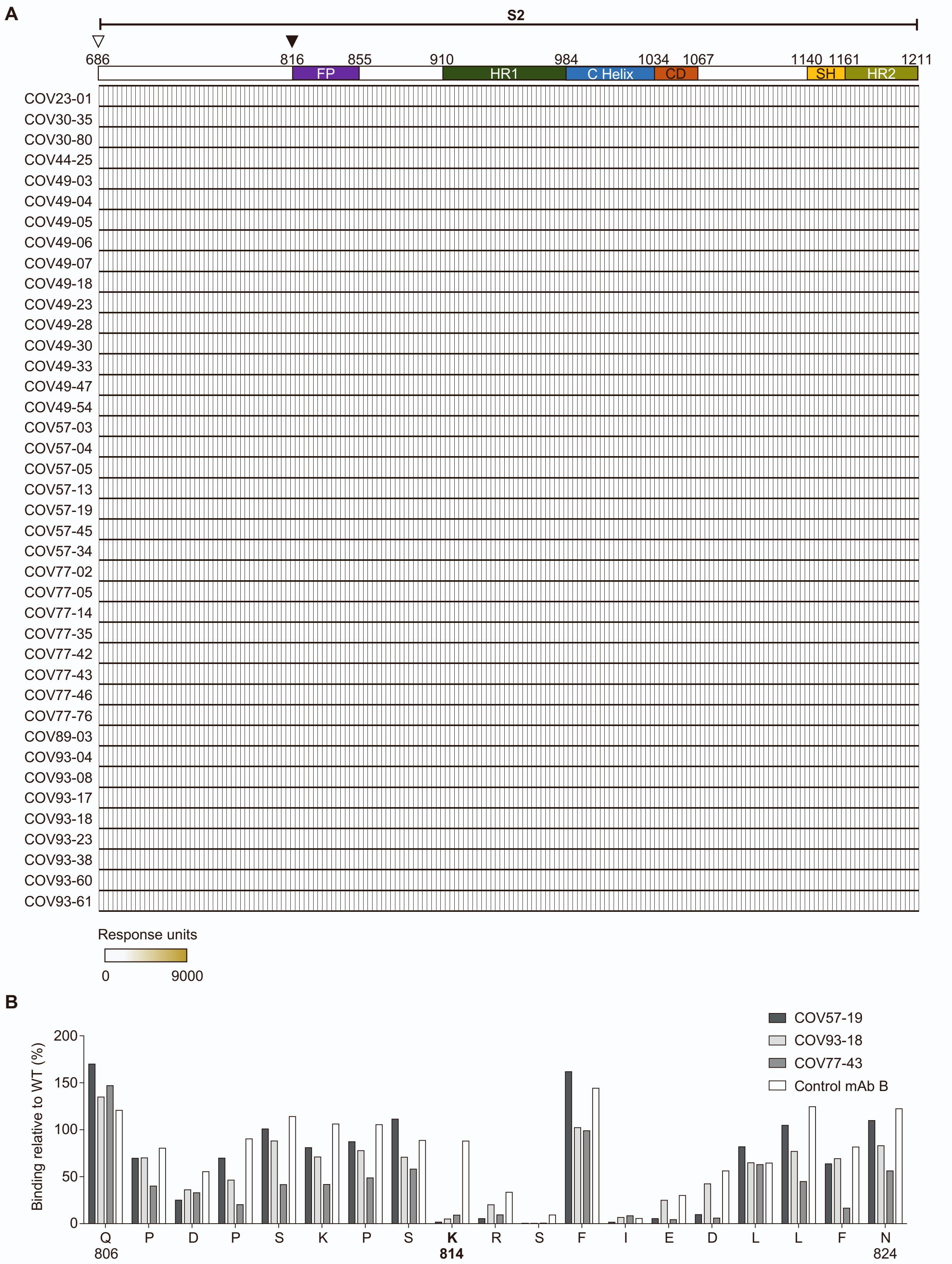

Figure S2. Group B mAbs bind to a conformational epitope that includes K814, related to Figure 2.

(A) The heat map shows the binding responses of Group B mAbs, 10 μg/mL, to an array of biotinylated peptides spanning the SARS-CoV-2 Wuhan-Hu-1 Spike S2 domain.

(B) Effect of mutations on binding of three group B mAbs in shotgun mutagenesis assay. A residue was considered critical if mutation of this residue resulted in a reduction of binding signal for the three group B mAbs but not control mAb B, which targets a conformational epitope not in this region.

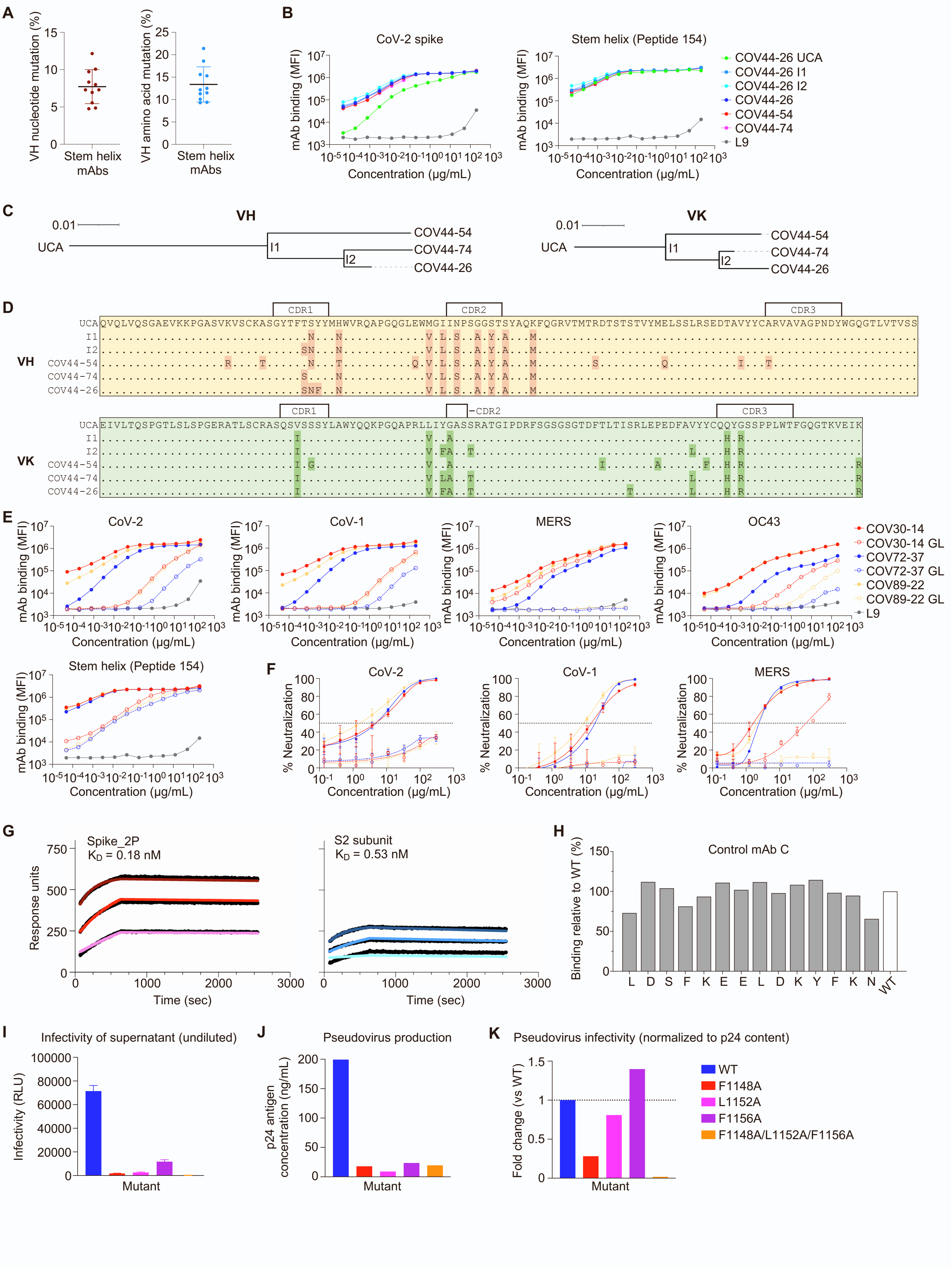
A
VH nucleotide mutation (%)
Stem helix mAbs
VH amino acid mutation (%)
Stem helix mAbs
B
CoV-2 spike
Stem helix (Peptide 154)
mAb binding (MFI)
Concentration (μg/mL)
COV44-26 UCA
COV44-26 I1
COV44-26 I2
COV44-26
COV44-54
COV44-74
L9
C
VH
VK
0.01
UCA
I1
I2
COV44-54
COV44-74
COV44-26
D
CDR1
CDR2
CDR3
UCA QVQLVQSGAEVKKPGASVKVSCKASGYTFTSYYMHWVRQAPGQGLEWMGIINPSGGSTSYAQKFQGRVTMTRDTSTSTVYMELSSLRSEDTAVYYCARVAVAGPNDYWGQGTLVTVSS
UCA EIVLTQSPGTLSLSPGERATLSCRASQSVSSSYLAWYQQKPGQAPRLLIYGASSRATGIPDRFSGSGSGTDFTLTISRLEPEDFAVYYCQQYGSSPPLWTFGQGTKVEIK
E
CoV-2
CoV-1
MERS
OC43
Stem helix (Peptide 154)
COV30-14
COV30-14 GL
COV72-37
COV72-37 GL
COV89-22
COV89-22 GL
L9
F
% Neutralization
G
Spike_2P
K_D = 0.18 nM
S2 subunit
K_D = 0.53 nM
Response units
Time (sec)
H
Control mAb C
Binding relative to WT (%)
L D S F K E E L D K Y F K N WT
I
Infectivity of supernatant (undiluted)
Infectivity (RLU)
Mutant
J
Pseudovirus production
p24 antigen concentration (ng/mL)
Mutant
K
Pseudovirus infectivity (normalized to p24 content)
Fold change (vs WT)
Mutant
WT
F1148A
L1152A
F1156A
F1148A/L1152A/F1156A

Figure S3. Characterization of stem helix-specific mAbs, related to Figure 3.

(A) Percentage mutation of VH nucleotides and VH amino acids for stem helix-specific mAbs, as inferred using IMGT (imgt.org). Error bars show mean ± SD.

(B) Binding of COV44-26-lineage antibodies to SARS-CoV-2 spike and peptide 154 from the stem helix region. L9 IgG1 (malaria-specific; [S1]) was included as a negative control mAb for binding experiments. Interconnected data points in the titration curves are shown without curve fitting.

(C) VH and VK lineage trees of the clonal family consisting of COV44-26, COV44-54 and COV44-74. The unmutated common ancestor (UCA) and intermediates (I1 and I2) were inferred using Cloanalyst [S2].

(D) Heavy and light chain sequence alignments of members of the COV44-26 lineage. CDR positions were determined using IMGT (imgt.org).

(E) Binding of VJ germline-reverted and mature COV30-14, COV72-37 and COV89-22 to spike of human coronaviruses. For the SARS-CoV-2 spike and stem helix peptide, the L9 negative control curves are the same as in Figure S3B.

(F) Neutralization of SARS-CoV-2, SARS-CoV and MERS-CoV pseudoviruses by VJ germline-reverted and mature COV30-14, COV72-37 and COV89-22. The dotted line represents 50% neutralization and error bars show mean ± SD.

(G) Kinetics of binding of COV89-22 Fab to SARS-CoV-2 spike protein, pre-fusion stabilized with two proline mutations (2P), as well as the unmodified S2 subunit of SARS-CoV-2 spike.

(H) Shotgun mutagenesis showing binding of control mAb C to residues in the stem helix region.

(I) Infectivity of undiluted supernatants containing WT SARS-CoV-2 pseudotyped virus, compared to F1148A, L1152A, F1156A (single), and F1148A/L1152A/F1156A (triple) spike mutants. RLU, relative light units. Error bars show mean ± SD.

(J) Pseudovirus production of WT SARS-CoV-2, as well as F1148A, L1152A, F1156A (single), and F1148A/L1152A/F1156A (triple) spike mutants, based on p24 antigen concentrations of each preparation.

(K) Normalized pseudovirus infectivity as calculated by ratio of infectivity in RLU to p24 antigen concentration in ng/mL, with the WT pseudovirus normalized to 1.

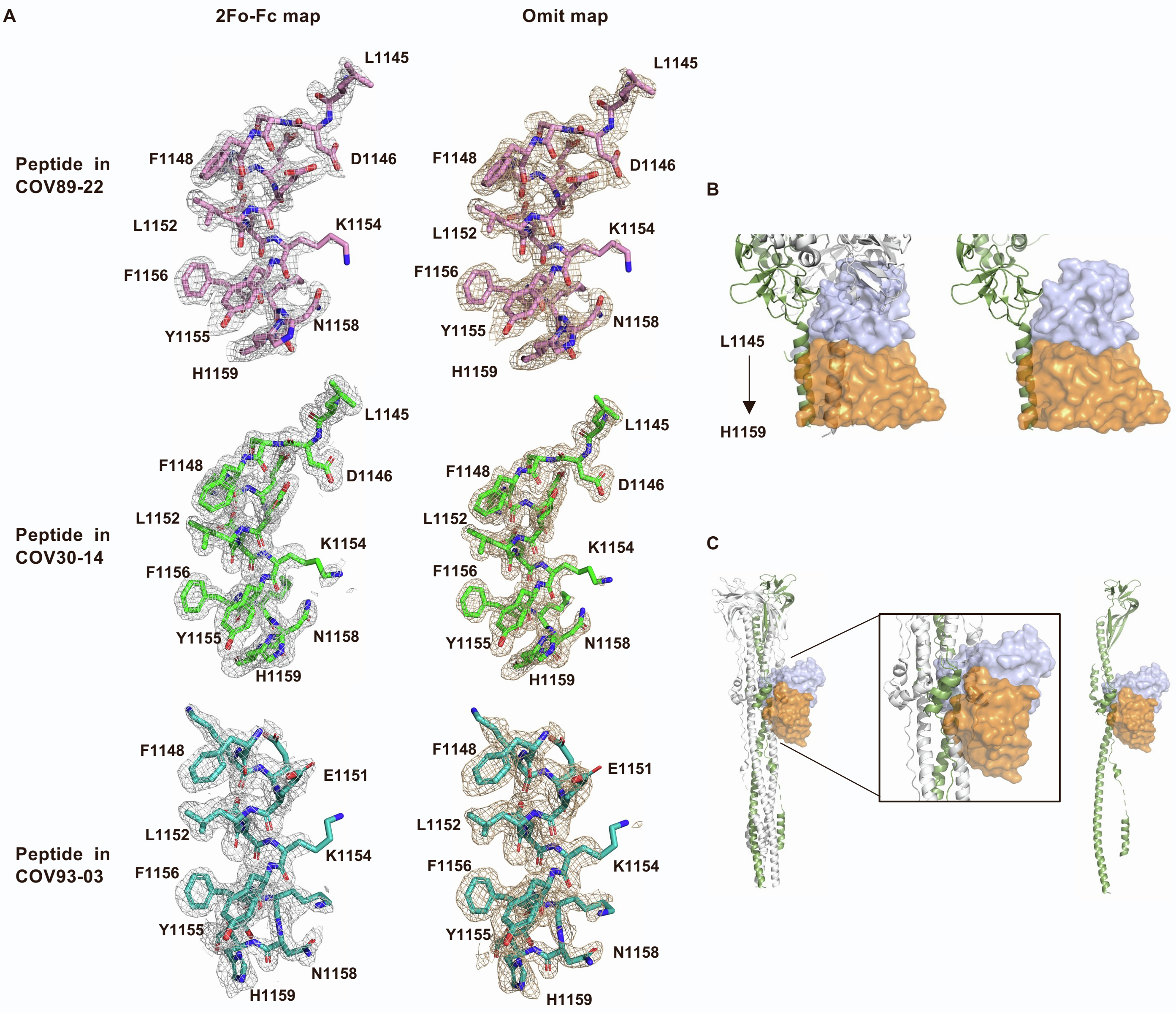

Figure S4. Electron density maps for the stem helix peptide and overall binding of COV89-22 to the stem helix peptide, related to Figures 4 and 5.

(A) The 2Fo-Fc electron density maps are represented in a gray mesh and contoured at 1.0σ for the stem helix peptide bound to COV89-22, COV30-14, and COV93-03, respectively. The Fo-Fc unbiased omit electron density maps are represented in a brown mesh and contoured at 2.0σ for each of the stem helix peptides.

(B) Location of stem helix peptide (L1145-H1159) on a protomer of the SARS-CoV-2 spike protein. Structures of COV89-22-peptide complex were superimposed onto the stem helix of an intact SARS-CoV-2 spike trimer and monomer structure in the pre-fusion state (PDB: 6XR8). The monomer shows the interaction surface of stem helix with COV89-22 which is located on the inside of three helix bundle in the stem region. COV89-22 would clash with the three-helix bundle in the stem region in the pre-fusion state. Conformational changes or conformational dynamics would be required for COV89-22 binding.

(C) The structures of the COV89-22-peptide complex was superimposed onto the stem helix of an intact SARS-CoV-2 spike trimer and monomer structure in the post-fusion state (PDB: 6XRA). Stabilization of an intermediate state would suggest a possible neutralization mechanism of COV89-22.

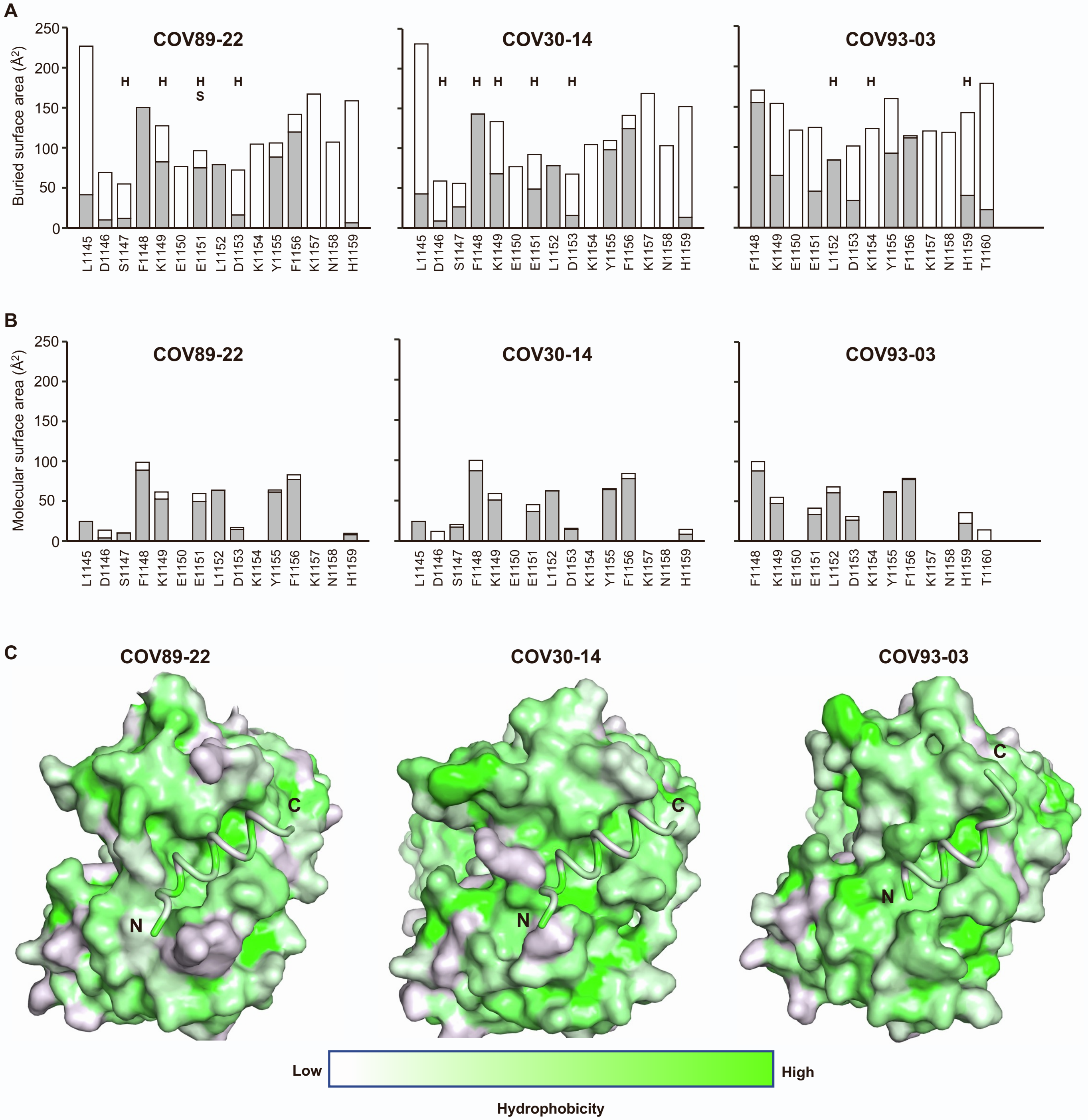

Figure S5. Buried molecular surface area and hydrophobicity of COV89-22, COV30-14, and COV93-03 in complex with SARS-CoV-2 stem helix peptides, related to Figures 4 and 5.

(A) Comparison of the peptide buried area of COV89-22, COV30-14, and COV93-03. Buried surface area (in gray) and accessible surface area (in white) of each residue of the stem helix peptide in complex with antibody is shown in the stacked bar chart. Residues of COV89-22, COV30-14 or COV93-03 that form polar interactions are denoted with "H" on top of each bar if they form a hydrogen-bond or "S" if they form a salt bridge. Buried and accessible surface areas were calculated with PISA [S3].

(B) Comparison of the peptide molecular surface contact area with COV89-22, COV30-14, and COV93-03. The molecular surface contact area for side chain (in gray) and main chain (in white) of each residue of the stem helix peptide in complex with antibody is shown in the stacked bar chart. Where there is no value, the peptide residue is not in contact with the antibody. The molecular surface contact areas were computed with the Molecular Surface package [S4].

(C) The SARS-CoV-2 stem peptide inserts into a hydrophobic groove shaped by the heavy and light chains of COV30-14, COV89-22, and COV93-03. Surfaces of the Fabs are colored in a green gradient by hydrophobicity calculated by Color h (https://pymolwiki.org/index.php/Color_h).

A

```
                                                      HCDR1
                                                      *   *
IGHV1-46  Q V Q L V Q S G A E V K K P G A S V K V S C K A S G Y T F T S Y Y M H W V R Q A
COV89-22  Q E Q L V Q S G A E V K K P G A S V K V S C K S S G F T F S Y F Y L H W V R Q A
COV30-14  Q V Q L V Q S G A E V K K P G A S V K V S C Q T S G Y T F T S Y Y M H W V R Q A
COV93-03  Q L Q L V Q S G A E V K K P G A S V K L S C K A S G N T F T S H Y I H W V R Q A
                                                                31      35
```

```
                              HCDR2
                    *   * *       *
IGHV1-46  P G Q G L E W M G I I N P S G G S T S Y A Q K F Q G R V T M T R D T S T S T V Y
COV89-22  P G Q G L E W M G I I N P R G D G T R Y A Q K F Q G R V T M T R D A S T G T L Y
COV30-14  P G Q G L E W M G L I T P S G D D T Y Y A Q R F Q G R V T M T R D T S T S P T Y
COV93-03  P G Q G P E W M G I I N P S G S G T R Y A Q K F Q G R V T M T R D T S T S T V Y
                    50  52 52a 53   57      65
```

```
                                          HCDR3
IGHV1-46  M E L S S L R S E D T A V Y Y C A R - - - - - - - - - - - - - - - - - - - - -
COV89-22  M E L R S L R S E D T A V Y Y C A R G A D H G A F D I W G Q G T M V T V S S
COV30-14  M E L S S L T S E D T A V Y Y C A K M S R A G G F D V W G Q G T L V T V S S
COV93-03  M E L V S L R F E D T A V Y Y C A G D Y - - - Y L D Y W G Q G T L V T V S S
                                          95        100 100a 101 102
```

B

```
                                                      LCDR1
                                                          *
IGKV3-20  E I V L T Q S P G T L S L S P G E R A T L S C R A S Q S V S S S Y L A W Y Q Q K
COV89-22  E I V L T Q S P G T L S L S P G E R A T L S C R A S Q S V R R N Y F A W Y Q Q K
COV30-14  E V V L T Q S P G T L S L S P G E R A T L S C R A S Q S I T G R Y L A W Y Q Q K
COV93-03  E F V L T Q S P G T L S L S P G E R A T L S C R A S Q S V T S N Y F A W Y Q Q K
                                                24    27 27a        34
```

```
                          LCDR2
IGKV3-20  P G Q A P R L L I Y G A S S R A T G I P D R F S G S G S G T D F T L T I S R L E
COV89-22  R G Q A P R L L I Y D A S T R A T G I P D R F S G S G S G T D F T L T I S R L E
COV30-14  P G Q A P R L L M Y G E S S R V T G I P D R F S G G G S G T D F T L T I S R L E
COV93-03  P G Q A P R L L I F G A S S R A T G I P D R F S G S G S G T D F T L T I S R L E
                              50      56
```

```
                          LCDR3
                            *   * * *
IGKV3-20  P E D F A V Y Y C Q Q Y G S S P - - - - - - - - - - - - - -
COV89-22  P E D F A V Y Y C Q Q Y D S S P P M Y I F G Q G T K L E I K
COV30-14  P E D F A V Y Y C Q H F A S S P P T Y T F G Q G T K L E I R
COV93-03  P E D F A V Y Y C Q Q Y G S S P P M Y T F G P G T K V E I K
                          89        95 95a 95b 96 97
```

C

Gene	FR1		CDR1 (33, 35)	FR2		CDR2 (50, 52, 57)	FR3			CDR3
IGHV1-2	QVQLVQSGA.EVKKP	GASVKVSCKAS	GYTF....TGYY	MHWVRQAP	GQGLEWMGR	INPN..SGGT	NYAQKFQ.G	RVTSTRDTSI	STAYMELSRLRS DDTVVYYC	AR
IGHV1-3	QVQLVQSGA.EVKKP	GASVKVSCKAS	GYTF....TSYA	MHWVRQAP	GQRLEWMGW	INAG..NGNT	KYSQKFQ.G	RVTITRDTSA	STAYMELSSLRS EDTAVYYC	AR
IGHV1-8	QVQLVQSGA.EVKKP	GASVKVSCKAS	GYTF....TSYD	INWVRQAT	GQGLEWMGW	MNPN..SGNT	GYAQKFQ.G	RVTMTRNTSI	STAYMELSSLRS EDTAVYYC	AR
IGHV1-18	QVQLVQSGA.EVKKP	GASVKVSCKAS	GYTF....TSYG	ISWVRQAP	GQGLEWMGW	ISAY..NGNT	NYAQKLQ.G	RVTMTTDTST	STAYMELRSLRS DDTAVYYC	AR
IGHV1-24	QVQLVQSGA.EVKKP	GASVKVSCKVS	GYTL....TELS	MHWVRQAP	GKGLEWMGG	FDPE..DGET	IYAQKFQ.G	RVTMTEDTST	DTAYMELSSLRS EDTAVYYC	AT
IGHV1-38-4	QVQLVQSWA.EVRKS	GASVKVSCSFS	GFTI....TSYG	IHWVQQSP	GQGLEWMGW	INPG..NGSP	SYAKKFQ.G	RFTMTRDMST	TTAYTDLSSLTS EDMAVYYY	AR
IGHV1-45	QMQLVQSGA.EVKKT	GSSVKVSCKAS	GYTF....TYRY	LHWVRQAP	GQALEWMGW	ITPF..NGNT	NYAQKFQ.D	RVTITRDRSM	STAYMELSSLRS EDTAMYYC	AR
**IGHV1-46**	**QVQLVQSGA.EVKKP**	**GASVKVSCKAS**	**GYTF....TSYY**	**MHWVRQAP**	**GQGLEWMGI**	**INPS..GGST**	**SYAQKFQ.G**	**RVTMTRDTST**	**STVYMELSSLRS EDTAVYYC**	**AR**
IGHV1-58	QMQLVQSGP.EVKKP	GTSVKVSCKAS	GFTF....TSSA	VQWVRQAR	GQRLEWIGW	IVVG..SGNT	NYAQKFQ.E	RVTITRDMST	STAYMELSSLRS EDTAVYYC	AA
IGHV1-68	QVQLGQSEA.EVKKP	GASVKVSCKAS	GYTF....TCCS	LHWLQQAP	GQGLERMRW	ITLY..NGNT	NYAKKFQ.G	RVTITRDMSL	RTAYIELSSLRS EDSAVYYW	AR
IGHV1-69	QVQLVQSGA.EVKKP	GSSVKVSCKAS	GGTF....SSYA	ISWVRQAP	GQGLEWMGG	IIPI..FGTA	NYAQKFQ.G	RVTITADEST	STAYMELSSLRS EDTAVYYC	AR
IGHV1-69-2	EVQLVQSGA.EVKKP	GATVKISCKVS	GYTF....TDYY	MHWVQQAP	GKGLEWMGL	VDPE..DGET	IYAEKFQ.G	RVTITADTST	DTAYMELSSLRS EDTAVYYC	AT
IGHV1-69D	QVQLVQSGA.EVKKP	GSSVKVSCKAS	GGTF....SSYA	ISWVRQAP	GQGLEWMGG	IIPI..FGTA	NYAQKFQ.G	RVTITADEST	STAYMELSSLRS EDTAVYYC	AR
IGHV1-NL1	QVQLLQPGV.QVKKP	GSSVKVSC*AS	RYTF....TKYF	TRWV*QSP	GQGHXWMG*	INPY..NDNT	HYAQTFW.G	RVTITSDRSM	STAYMELSXLRS EDMVVYYC	VR

Gene	FR1		CDR1 (32)	FR2		CDR2	FR3				CDR3 (91, 95)
IGKV3-7	EIVMTQSPPTLSLSP	GERVTLSCRAS	QSVS.....SSY	LTWYQQKP	GQAPRLLIY	GA.......S	TRATSIP.A	RFSGSG..SG	TDFTLTISSLQP	EDFAVYYC	QQDHNLP
IGKV3-11	EIVLTQSPATLSLSP	GERATLSCRAS	QSV......SSY	LAWYQQKP	GQAPRLLIY	DA.......S	NRATGIP.A	RFSGSG..SG	TDFTLTISSLEP	EDFAVYYC	QQRSNWP
IGKV3-15	EIVMTQSPATLSVSP	GERATLSCRAS	QSV......SSN	LAWYQQKP	GQAPRLLIY	GA.......S	TRATGIP.A	RFSGSG..SG	TEFTLTISSLQS	EDFAVYYC	QQYNNWP
**IGKV3-20**	**EIVLTQSPGTLSLSP**	**GERATLSCRAS**	**QSVS.....SSY**	**LAWYQQKP**	**GQAPRLLIY**	**GA.......S**	**SRATGIP.D**	**RFSGSG..SG**	**TDFTLTISRLEP**	**EDFAVYYC**	**QQYGSSP**
IGKV3D-7	EIVMTQSPATLSLSP	GERATLSCRAS	QSVS.....SSY	LSWYQQKP	GQAPRLLIY	GA.......S	TRATGIP.A	RFSGSG..SG	TDFTLTISSLQP	EDFAVYYC	QQDYNLP
IGKV3D-11	EIVLTQSPATLSLSP	GERATLSCRAS	QGV......SSY	LAWYQQKP	GQAPRLLIY	DA.......S	NRATGIP.A	RFSGSG..PG	TDFTLTISSLEP	EDFAVYYC	QQRSNWH
IGKV3D-15	EIVMTQSPATLSVSP	GERATLSCRAS	QSV......SSN	LAWYQQKP	GQAPRLLIY	GA.......S	TRATGIP.A	RFSGSG..SG	TEFTLTISSLQS	EDFAVYYC	QQYNNWP
IGKV3D-20	EIVLTQSPATLSLSP	GERATLSCGAS	QSVS.....SSY	LAWYQQKP	GLAPRLLIY	DA.......S	SRATGIP.D	RFSGSG..SG	TDFTLTISRLEP	EDFAVYYC	QQYGSSP

Figure S6. Comparison of COV89-22, COV30-14 and COV93-03 sequences to the IGHV1-46 and IGKV3-20 germlines, related to Figures 4 and 5.

(A) Alignment of the heavy-chain variable domain sequences of COV89-22, COV30-14, and COV93-03 with the human germline IGHV1-46 sequence.

(B) Alignment of the light-chain variable domain sequences of COV89-22, COV30-14, and COV93-03 with the human germline IGKV3-20 sequence. Sequences that correspond to HCDR1, HCDR2, HCDR3, LCDR1, LCDR2, and LCDR3 are indicated. Residues that differ from the germline are in red. Residues interacting with the stem helix peptide are highlighted in yellow. Residue are labeled in Kabat numbering. Black asterisks represent residues identical to germline that interact with the stem helix. The orange asterisk represents the residue identical to germline but interacts in only 2 of 3 antibodies with the stem helix. Green asterisks represent residues that are identical in 2 of 3 antibodies to germline, but still all interact with the stem helix. CDR positions were determined using IgBlast (https://www.ncbi.nlm.nih.gov/igblast/).

(C) Alignment of IGHV1- and IGKV3-encoded amino acid sequences. Amino acids in green are important contact residues for the stem helix and those highlighted in red are unique residues to IGHV1-46 or unique motifs to IGKV3-20/IGKV3D-20.

A

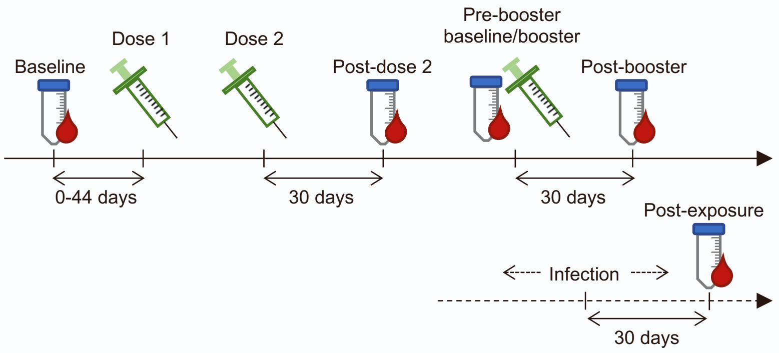

B

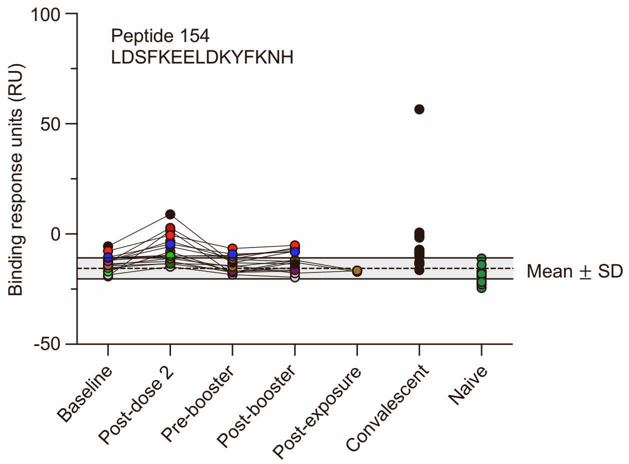

Analysis	*P-value*
Post-dose 2 v Baseline	**<0.001**
Post-dose 2 v Pre-booster	**<0.001**
Post-booster v Pre-booster	1
Post-booster v Baseline	1
Post-dose 2 v Naïve	**0.0182**
Convalescent v Post-dose 2	1
Convalescent v Naïve	**0.0049**

Figure S7. Polyclonal antibody responses to the stem helix after mRNA-1273 vaccination and SARS-CoV-2 infection, related to Figure 2.

(A) Timeline of plasma/serum sample collection and Moderna mRNA-1273 vaccination. Plasma was collected 30 days after documented SARS-CoV-2 infection for three out of 16 volunteers.

(B) Polyclonal IgG reactivity from mRNA-1273-vaccinated (n=16), convalescent unvaccinated (n=16) and COVID-19 naïve (n=13) individuals to peptide 154 from the stem helix region in the S2 subunit. All polyclonal IgG samples were tested at a 100 µg/mL concentration. Background is shown as mean ± SD of donors in the baseline and COVID-19-naïve groups. Pairwise comparisons were made from a nested, mixed-model ANOVA with Bonferroni-adjusted P-values.

Table S1. X-ray data collection and refinement statistics, related to Figures 4 and 5.

**Data collection**	COV89-22+ peptide 154	COV30-14+ peptide 154	COV93-03+ peptide 155
Beamline	SSRL12-1	APS 23-ID-B	APS 23-ID-B
Wavelength (Å)	0.9795	1.033	1.033
Space group	$P2_1$	$P2_12_12_1$	$P2_12_12_1$
Unit cell parameters			
a, b, c (Å)	42.08 76.91 154.38	75.36 83.20 170.38	65.86 65.97 197.65
α, β, γ (°)	90 93.8 90	90 90 90	90 90 90
Resolution (Å) [a]	50-1.6 (1.66-1.60)	50-1.5 (1.55-1.50)	50-1.75 (1.81-1.75)
Unique reflections [a]	99,459 (2,721)	137,051 (5,040)	80,275 (4,909)
Redundancy [a]	6.0 (3.7)	11.7 (6.0)	9.8 (3.4)
Completeness (%) [a]	77.3 (17.7)	80.5 (27.3)	92.6 (52.6)
$<I/\sigma_I>$ [a]	21.3 (0.9)	40.5 (0.8)	22.8 (0.9)
R_{sym}[b] (%) [a]	8.4 (94)	6.9 (>100)	13.8 (65)
R_{pim}[b] (%) [a]	3.6 (55)	2.0 (46)	4.3 (40)
$CC_{1/2}$[c] (%) [a]	99.4 (52.6)	99.9 (51.0)	99.1 (52.0)
**Refinement statistics**			
Resolution (Å)	42.71-1.60	46.9-1.5	46.6-1.75
Reflections (work)	99,449	137,013	80,269
Reflections (test)	4972	6742	4017
R_{cryst}[d] / R_{free}[e] (%)	18.5/21.0	19.8/22.1	19.4/22.3
No. of copies in ASU	2	2	2
No. of atoms	8002	7825	7463
Fab	6694	6487	6473
Peptide	270	270	215
Solvent	1038	1068	775
Average *B*-values ($Å^2$)	27	31	32
Fab	25	30	31
Peptide	36	29	35
Solvent	35	37	39
Wilson *B*-value ($Å^2$)	20	21	26
**RMSD from ideal geometry**			
Bond length (Å)	0.005	0.006	0.004
Bond angle (°)	0.79	0.86	0.76
**Ramachandran statistics (%)**[f]			
Favored	98.5	98.4	98.3
Outliers	0.00	0.00	0.12
**PDB code**	8DTX	8DTR	8DTT

[a] Numbers in parentheses refer to the highest resolution shell.

[b] $R_{sym} = \Sigma_{hkl} \Sigma_i | I_{hkl,i} - <I_{hkl}> | / \Sigma_{hkl} \Sigma_i I_{hkl,i}$ and $R_{pim} = \Sigma_{hkl} (1/(n-1))^{1/2} \Sigma_i | I_{hkl,i} - <I_{hkl}> | / \Sigma_{hkl} \Sigma_i I_{hkl,i}$, where $I_{hkl,i}$ is the scaled intensity of the ith measurement of reflection h, k, l, $<I_{hkl}>$ is the average intensity for that reflection, and *n* is the redundancy.

[c] $CC_{1/2}$ = Pearson correlation coefficient between two random half datasets.

[d] $R_{cryst} = \Sigma_{hkl} | F_o - F_c | / \Sigma_{hkl} | F_o |$ x 100, where F_o and F_c are the observed and calculated structure factors, respectively.

[e] R_{free} was calculated as for R_{cryst}, but on a test set comprising 5% of the data excluded from refinement.

[f] From MolProbity [S5].

Table S2. Coronavirus neutralization by IGHV1-46/IGKV3-20 stem helix-specific mAbs ($Assay_{NIH}$), related to Figure 6.

mAb	Pseudovirus neutralization (NT_{50}) (μg/mL)			
	SARS-CoV-2 (WA-1)	SARS-CoV	MERS-CoV	NL63
**COV30-14**	35.9	6.54	0.228	>100
**COV72-37**	33.2	7.10	0.863	>100
**COV89-22**	13.8	1.95	1.24	>100

Table S3. Primers for generation of SARS-CoV-2 spike mutants, related to STAR Methods.

	Forward primer	Reverse primer
**F1148A**	gaactggactccGCcaaggaggaactggac	tcctccttggcggagtccagttcaggttggag
**L1152A**	ccttcaaggaggaaGCTgacaaatacttcaagaaccac	cttgaagtatttgtcAGCttcctccttgaaggagtccagttc
**F1156A**	actggacaaatacGCcaagaaccacaccagc	tggtgtggttcttgGCgtatttgtccagttcctc
**F1148A/ L1152A/ F1156A**	GCcaaggaggaaGCTgacaaatacGCcaagaaccacac	gtatttgtcAGCttcctccttgGCggagtccagttcaggttgga
**End**	ctggacaaggtggaggctgag	caagcttccatggctcgagtcacttacaacaggagccacagga

[a]Capitalized nucleotides are those that differ from the wild-type sequence and were used to generate the mutants.

Supplementary References

S1. Wang, L.T., Pereira, L.S., Flores-Garcia, Y., O'Connor, J., Flynn, B.J., Schon, A., Hurlburt, N.K., Dillon, M., Yang, A.S.P., Fabra-Garcia, A., et al. (2020). A potent anti-malarial human monoclonal antibody targets circumsporozoite protein minor repeats and neutralizes sporozoites in the liver. Immunity *53*, 733-744.e8. 10.1016/j.immuni.2020.08.014.

S2. Kepler, T.B. (2013). Reconstructing a B-cell clonal lineage. I. Statistical inference of unobserved ancestors. F1000Res *2*, 103. 10.12688/f1000research.2-103.v1.

S3. Krissinel, E., and Henrick, K. (2007). Inference of macromolecular assemblies from crystalline state. J Mol Biol *372*, 774-797. 10.1016/j.jmb.2007.05.022.

S4. Connolly, M.L. (1983). Analytical molecular surface calculation. J Appl Crystallogr *16*, 548-558. 10.1107/S0021889883010985.

S5. Chen, V.B., Arendall, W.B., 3rd, Headd, J.J., Keedy, D.A., Immormino, R.M., Kapral, G.J., Murray, L.W., Richardson, J.S., and Richardson, D.C. (2010). MolProbity: all-atom structure validation for macromolecular crystallography. Acta Crystallogr D Biol Crystallogr *66*, 12-21. 10.1107/S0907444909042073.
