# Supplementary material for: Rare, convergent antibodies targeting the stem helix broadly neutralize diverse betacoronaviruses
Source: Cell Host Microbe. 2023 Jan 11;31(1):97–111.e12. doi: 10.1016/j.chom.2022.10.010 (PMC9639329; doi:10.1016/j.chom.2022.10.010)
Supplement: Document S2. Article plus supplemental information [file mmc2.pdf]

Article

Cell Host & Microbe

Rare, convergent antibodies targeting the stem helix broadly neutralize diverse betacoronaviruses

Graphical abstract

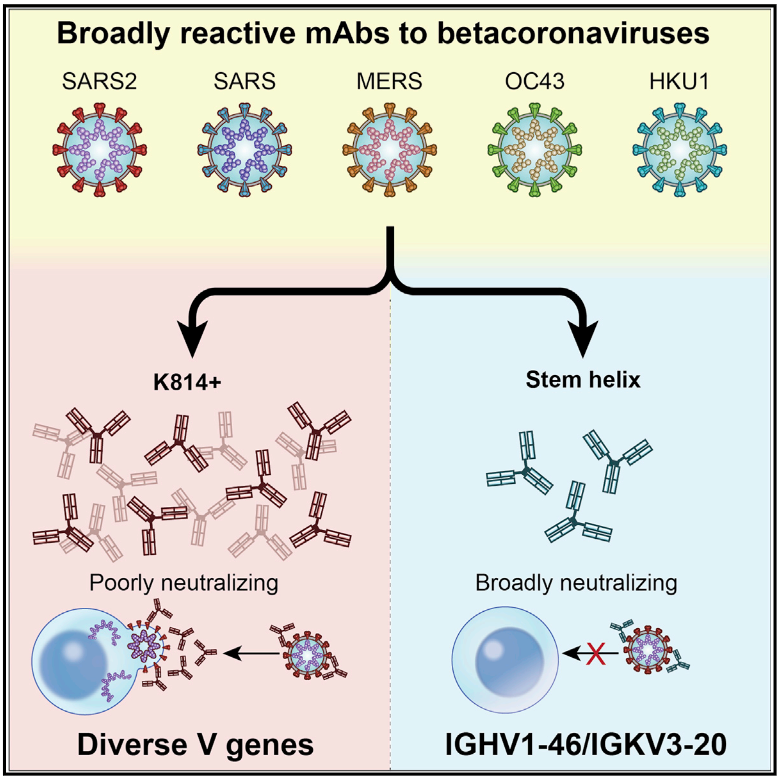

Highlights

- Isolation of 55 broadly reactive monoclonal antibodies to betacoronaviruses
- Only a minority are broadly neutralizing and target the stem helix
- Identification of an IGHV1-46/IGKV3-20 gene signature of the broad neutralizers
- Structures of IGHV1-46/IGKV3-20 antibodies reveal a conserved mode of binding

Authors

Cherrelle Dacon, Linghang Peng, Ting-Hui Lin, ..., David Nemazee, Ian A. Wilson, Joshua Tan

Correspondence

tanj4@nih.gov

In brief

The characteristics of antibodies that broadly neutralize coronaviruses are poorly understood. Here, Dacon et al. identify a class of stem helix-specific monoclonal antibodies from COVID-19 convalescent donors that neutralize diverse betacoronaviruses, use an IGHV1-46/IGKV3-20 gene signature, and bind in a conserved manner to the spike protein.

Dacon et al., 2023, Cell Host & Microbe *31*, 97–111
January 11, 2023 Published by Elsevier Inc.
https://doi.org/10.1016/j.chom.2022.10.010

CellPress

Cell Host & Microbe

CellPress
OPEN ACCESS

Article

Rare, convergent antibodies targeting the stem helix broadly neutralize diverse betacoronaviruses

Cherrelle Dacon,[1,16] Linghang Peng,[2,16] Ting-Hui Lin,[3,16] Courtney Tucker,[1,4,16] Chang-Chun D. Lee,[3,16] Yu Cong,[5] Lingshu Wang,[6] Lauren Purser,[1] Andrew J.R. Cooper,[1] Jazmean K. Williams,[7] Chul-Woo Pyo,[8] Meng Yuan,[3] Ivan Kosik,[9] Zhe Hu,[9] Ming Zhao,[10] Divya Mohan,[1] Mary Peterson,[11] Jeff Skinner,[11] Saurabh Dixit,[5] Erin Kollins,[5] Louis Huzella,[5] Donna Perry,[5] Russell Byrum,[5] Sanae Lembirik,[5] Michael Murphy,[5] Yi Zhang,[6] Eun Sung Yang,[6] Man Chen,[6] Kwanyee Leung,[6] Rona S. Weinberg,[12] Amarendra Pegu,[6] Daniel E. Geraghty,[8] Edgar Davidson,[7] Benjamin J. Doranz,[7] Iyadh Douagi,[13] Susan Moir,[14] Jonathan W. Yewdell,[9] Connie Schmaljohn,[5] Peter D. Crompton,[11] John R. Mascola,[6] Michael R. Holbrook,[5] David Nemazee,[2] Ian A. Wilson,[3,15] and Joshua Tan[1,17,*]

[1]Antibody Biology Unit, Laboratory of Immunogenetics, National Institute of Allergy and Infectious Diseases, National Institutes of Health, Rockville, MD 20852, USA
[2]Department of Immunology and Microbiology, The Scripps Research Institute, La Jolla, CA 92037, USA
[3]Department of Integrative Structural and Computational Biology, The Scripps Research Institute, La Jolla, CA 92037, USA
[4]Department of Biology, The Catholic University of America, Washington, DC 20064, USA
[5]Integrated Research Facility, Division of Clinical Research, National Institute of Allergy and Infectious Diseases, National Institutes of Health, Frederick, MD 21702, USA
[6]Vaccine Research Center, National Institute of Allergy and Infectious Diseases, National Institutes of Health, Bethesda, MD 20892, USA
[7]Integral Molecular, Philadelphia, PA 19104, USA
[8]Clinical Research Division, Fred Hutchinson Cancer Research Center, Seattle, WA 98109, USA
[9]Cellular Biology Section, Laboratory of Viral Diseases, National Institute of Allergy and Infectious Diseases, National Institutes of Health, Bethesda, MD 20892, USA
[10]Protein Chemistry Section, Research Technologies Branch, National Institute of Allergy and Infectious Diseases, National Institutes of Health, Rockville, MD 20852, USA
[11]Malaria Infection Biology and Immunity Section, Laboratory of Immunogenetics, National Institute of Allergy and Infectious Diseases, National Institutes of Health, Rockville, MD 20852, USA
[12]New York Blood Center, Lindsley F. Kimball Research Institute, New York, NY 10065, USA
[13]Flow Cytometry Section, Research Technologies Branch, National Institute of Allergy and Infectious Diseases, National Institutes of Health, Bethesda, MD 20892, USA
[14]B Cell Immunology Section, Laboratory of Immunoregulation, National Institute of Allergy and Infectious Diseases, National Institutes of Health, Bethesda, MD 20892, USA
[15]The Skaggs Institute for Chemical Biology, The Scripps Research Institute, La Jolla, CA 92037, USA
[16]These authors contributed equally
[17]Lead contact
*Correspondence: tanj4@nih.gov
https://doi.org/10.1016/j.chom.2022.10.010

SUMMARY

Humanity has faced three recent outbreaks of novel betacoronaviruses, emphasizing the need to develop approaches that broadly target coronaviruses. Here, we identify 55 monoclonal antibodies from COVID-19 convalescent donors that bind diverse betacoronavirus spike proteins. Most antibodies targeted an S2 epitope that included the K814 residue and were non-neutralizing. However, 11 antibodies targeting the stem helix neutralized betacoronaviruses from different lineages. Eight antibodies in this group, including the six broadest and most potent neutralizers, were encoded by IGHV1-46 and IGKV3-20. Crystal structures of three antibodies of this class at 1.5–1.75-Å resolution revealed a conserved mode of binding. COV89-22 neutralized SARS-CoV-2 variants of concern including Omicron BA.4/5 and limited disease in Syrian hamsters. Collectively, these findings identify a class of IGHV1-46/IGKV3-20 antibodies that broadly neutralize betacoronaviruses by targeting the stem helix but indicate these antibodies constitute a small fraction of the broadly reactive antibody response to betacoronaviruses after SARS-CoV-2 infection.

INTRODUCTION

Betacoronaviruses constitute one of four coronavirus genera and are a major cause of respiratory disease (V'Kovski et al., 2021). They can be divided into five subgenera, of which three currently contain members that are pathogenic to humans. HCoV-OC43 and HCoV-HKU1 are lineage A betacoronaviruses that cause mild upper respiratory disease, whereas MERS-CoV (lineage C), SARS-CoV, and SARS-CoV-2 (lineage B) are responsible for severe outbreaks that led to a large number of

This is an open access article under the CC BY license (http://creativecommons.org/licenses/by/4.0/).

deaths in the past 20 years (Fung and Liu, 2019). SARS-CoV-2, the causative agent of COVID-19, has claimed more than six million lives since the first cases emerged in late 2019 (Dong et al., 2020). The currently dominant SARS-CoV-2 Omicron subvariant BA.5 is resistant to most monoclonal antibody (mAb) therapeutics available in the clinic (Yamasoba et al., 2022; Takashita et al., 2022b). Furthermore, other betacoronaviruses infect a range of animal species that regularly come into contact with humans, increasing the possibility of future zoonotic spillover (Peck et al., 2015). Therefore, there is an urgent need to develop vaccines and therapeutic mAbs that broadly target betacoronaviruses.

The major immune target on the coronavirus surface is the spike protein, a homotrimeric type I viral fusion protein that is composed of two subunits, S1 and S2 (Li, 2016). The S1 subunit uses either its N-terminal domain (NTD) or C-terminal domain (CTD) as the receptor-binding domain (RBD) to engage host cell receptors. Following receptor engagement, the S2 subunit undergoes conformational rearrangements to bridge and fuse the virus and host cell membrane, allowing the release of virus genetic material into the host cell cytoplasm. The SARS-CoV-2 spike protein is the target of currently available COVID-19 vaccines and therapeutic mAbs (Edwards et al., 2022). Although these vaccines are predominantly based on whole-spike constructs, most of the neutralizing antibody response following immunization is thought to be directed against the RBD. Similarly, all therapeutic mAbs available for public use target this domain. However, given the poor conservation of the RBD across different betacoronavirus lineages (Li, 2015), these vaccines and therapies are unlikely to be effective against betacoronaviruses that are distantly related to SARS-CoV-2. Instead, more conserved regions of the spike protein may be more suitable for the design of vaccines that cover a wider range of betacoronaviruses.

Here, we performed an epitope-agnostic screen to identify mAbs that broadly neutralize betacoronaviruses, with the goal of studying the nature of these antibodies and the characteristics of their target epitopes. We found that the majority of broadly reactive mAbs were non-neutralizing and bound to an epitope that included the K814 residue. However, 11 mAbs targeted the conserved stem helix in the S2 subunit and cross-neutralized betacoronaviruses from different subgenera, highlighting the importance of this site as a target of neutralizing antibodies in conjunction with reports from previous studies (Li et al., 2022; Pinto et al., 2021; Sauer et al., 2021; Wang et al., 2021; Zhou et al., 2022b). Eight of these mAbs, isolated from multiple donors, used the same germline gene combination of IGHV1-46/IGKV3-20. Crystal structures of three Fab-peptide complexes of antibodies COV89-22, COV30-14, and COV93-03 revealed that they all targeted the stem helix in a similar way. Two IGHV1-46/IGKV3-20 mAbs, COV89-22 and COV72-37, limited disease in the Syrian hamster model. In summary, these data suggest that the broadly reactive antibody response to betacoronaviruses after SARS-CoV-2 infection largely focuses on an immunodominant, weakly neutralizing site, but a minor part of this response consists of broadly neutralizing mAbs with shared gene usage that target the stem helix. Therefore, stem helix-specific vaccine constructs that elicit this antibody class may be an efficient way to generate protective antibody responses to betacoronaviruses, including all SARS-CoV-2 variants of concern.

RESULTS

Identification of mAbs that broadly neutralize betacoronaviruses

To isolate mAbs with broad reactivity, we selected 19 COVID-19 convalescent donors that had plasma reactivity to diverse betacoronaviruses from a previously described cohort (Cho et al., 2021). A total of 673,671 IgG^+ and 305,142 IgA^+ memory B cells (MBCs) from these donors were screened in a two-step workflow that utilized sequential oligoclonal and monoclonal B cell culture to downselect B cells of interest. Recombinant mAbs were screened for binding to spike protein from the betacoronaviruses SARS-CoV-2, SARS-CoV, MERS-CoV, HCoV-HKU1, and HCoV-OC43, as well as from the alphacoronaviruses HCoV-NL63 and HCoV-229E. We isolated six mAbs that targeted multiple coronavirus genera by binding the conserved fusion peptide, as recently described (Dacon et al., 2022). From this screen, we also obtained a panel of 54 IgG mAbs and one IgA mAb that were broadly reactive to betacoronaviruses but were mostly unreactive to alphacoronavirus spike proteins, with a few exceptions. All 55 mAbs bound to both SARS-CoV-2 and SARS-CoV spike, and the overwhelming majority of mAbs (53 of 55) also bound to HCoV-OC43 spike (Figure 1). Furthermore, 70.9% (n = 39) of the mAbs bound to the spike proteins of all five human-infecting betacoronaviruses. We next screened the 55 broadly reactive mAbs in neutralization assays against SARS-CoV-2, SARS-CoV, MERS-CoV, and HCoV-NL63 envelope pseudoviruses, as well as authentic HCoV-OC43, to assess the breadth and potency of their neutralization. Eighteen mAbs neutralized at least one virus, among which the mAbs COV89-22, COV30-14, COV72-37, COV44-26, and COV44-74 neutralized all four of the human betacoronaviruses tested (Figure 1). However, the majority of mAbs were non-neutralizing, and, consistent with their spike binding being largely restricted to the betacoronavirus genera, none of the 55 mAbs neutralized the alphacoronavirus HCoV-NL63 even at the highest concentration tested (100 μg/mL).

Broadly neutralizing mAbs against betacoronaviruses target the stem helix

To identify the spike domain targeted by these broadly reactive mAbs, we tested the mAb panel for binding to SARS-CoV-2 RBD, NTD, S1, and S2. Flow cytometry analyses revealed the SARS-CoV-2 spike S2 subunit as the target of the majority of the mAbs (Figure S1A). Subsequent surface plasmon resonance (SPR)-based epitope binning analysis demonstrated that the mAbs could be separated into two groups that were distinct from control mAbs targeting the fusion peptide (Figure S1B) (Dacon et al., 2022). The mAbs sorted into Group A (n = 11) competed for epitope binding with a previously described stem helix-targeting mAb S2P6 (Pinto et al., 2021), whereas mAbs sorted into Group B (n = 40) bound to a separate epitope on the S2 subunit. To further investigate the specific binding sites of these mAbs, we performed peptide mapping using an array of overlapping 15-mer biotinylated peptides spanning the S2 subunit of SARS-CoV-2. Consistent with the epitope binning analysis, antibodies in Group A bound to peptides covering the $_{1142}$QPELDSFKEELDKYFKNHTSP$_{1162}$ sequence in the stem helix region of SARS-CoV-2 (Figure 2A). However, antibodies in

CellPress
OPEN ACCESS

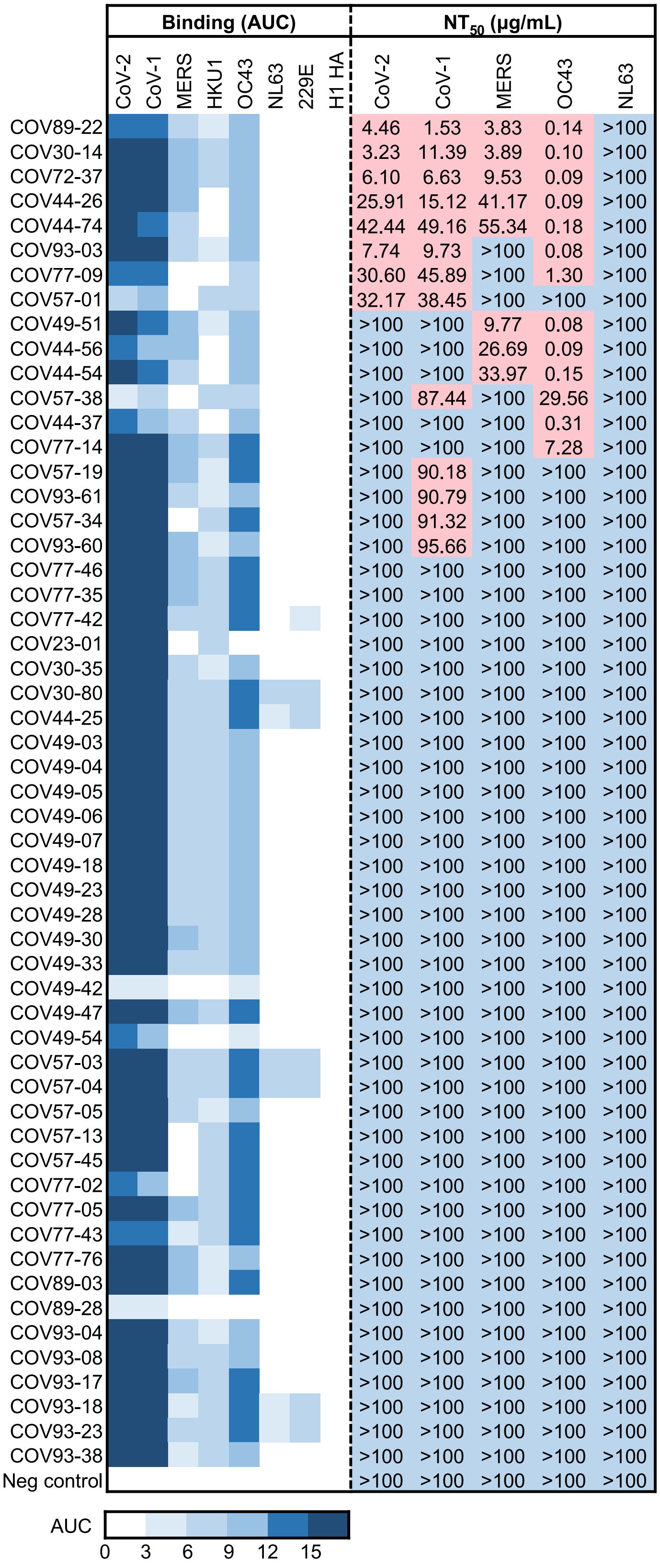

	NT50 (μg/mL) CoV-2	CoV-1	MERS	OC43	NL63
COV89-22	4.46	1.53	3.83	0.14	>100
COV30-14	3.23	11.39	3.89	0.10	>100
COV72-37	6.10	6.63	9.53	0.09	>100
COV44-26	25.91	15.12	41.17	0.09	>100
COV44-74	42.44	49.16	55.34	0.18	>100
COV93-03	7.74	9.73	>100	0.08	>100
COV77-09	30.60	45.89	>100	1.30	>100
COV57-01	32.17	38.45	>100	>100	>100
COV49-51	>100	>100	9.77	0.08	>100
COV44-56	>100	>100	26.69	0.09	>100
COV44-54	>100	>100	33.97	0.15	>100
COV57-38	>100	87.44	>100	29.56	>100
COV44-37	>100	>100	>100	0.31	>100
COV77-14	>100	>100	>100	7.28	>100
COV57-19	>100	90.18	>100	>100	>100
COV93-61	>100	90.79	>100	>100	>100
COV57-34	>100	91.32	>100	>100	>100
COV93-60	>100	95.66	>100	>100	>100
COV77-46	>100	>100	>100	>100	>100
COV77-35	>100	>100	>100	>100	>100
COV77-42	>100	>100	>100	>100	>100
COV23-01	>100	>100	>100	>100	>100
COV30-35	>100	>100	>100	>100	>100
COV30-80	>100	>100	>100	>100	>100
COV44-25	>100	>100	>100	>100	>100
COV49-03	>100	>100	>100	>100	>100
COV49-04	>100	>100	>100	>100	>100
COV49-05	>100	>100	>100	>100	>100
COV49-06	>100	>100	>100	>100	>100
COV49-07	>100	>100	>100	>100	>100
COV49-18	>100	>100	>100	>100	>100
COV49-23	>100	>100	>100	>100	>100
COV49-28	>100	>100	>100	>100	>100
COV49-30	>100	>100	>100	>100	>100
COV49-33	>100	>100	>100	>100	>100
COV49-42	>100	>100	>100	>100	>100
COV49-47	>100	>100	>100	>100	>100
COV49-54	>100	>100	>100	>100	>100
COV57-03	>100	>100	>100	>100	>100
COV57-04	>100	>100	>100	>100	>100
COV57-05	>100	>100	>100	>100	>100
COV57-13	>100	>100	>100	>100	>100
COV57-45	>100	>100	>100	>100	>100
COV77-02	>100	>100	>100	>100	>100
COV77-05	>100	>100	>100	>100	>100
COV77-43	>100	>100	>100	>100	>100
COV77-76	>100	>100	>100	>100	>100
COV89-03	>100	>100	>100	>100	>100
COV89-28	>100	>100	>100	>100	>100
COV93-04	>100	>100	>100	>100	>100
COV93-08	>100	>100	>100	>100	>100
COV93-17	>100	>100	>100	>100	>100
COV93-18	>100	>100	>100	>100	>100
COV93-23	>100	>100	>100	>100	>100
COV93-38	>100	>100	>100	>100	>100
Neg control	>100	>100	>100	>100	>100

Figure 1. Identification of broadly neutralizing antibodies against human betacoronaviruses

The area under the curve (AUC) from titration of mAb binding to spike proteins from human betacoronaviruses SARS-CoV-2 Wuhan-Hu-1 (CoV-2), SARS-CoV (CoV-1), MERS-CoV, HCoV-HKU1, and HCoV-OC43, as well as alphacoronaviruses HCoV-NL63 and HCoV-229E, is shown on the panel of the left. Influenza H1 hemagglutinin (HA) was included as a control antigen and L9 IgG1 (malaria specific; Wang et al., 2020) was included as a negative control mAb for binding experiments. AUC values for each antigen are shown after subtraction with values for the negative control antigen CD4. The antibody titers at 50% neutralization (NT_{50}) against SARS-CoV-2 Wuhan-Hu-1, SARS-CoV, MERS-CoV, HCoV-NL63 envelope-pseudotyped virus, as well as authentic HCoV-OC43, are shown on the right. Neutralizing mAbs are ranked by their breadth of neutralization and the geometric mean of their NT_{50} values. Cells highlighted in blue denote mAbs that did not show neutralizing potency at the highest concentration tested (100 μg/mL). Negative control mAbs for neutralization are DEN3 (dengue-specific; Rogers et al., 2020) for SARS-CoV-2, SARS-CoV, MERS-CoV and HCoV-NL63, and CV503 (SARS-CoV-2 RBD-specific; Cho et al., 2021) for HCoV-OC43. NT_{50} values were calculated using the dose-response-inhibition model with 5-parameter Hill slope equation in GraphPad Prism version 9.3.1.

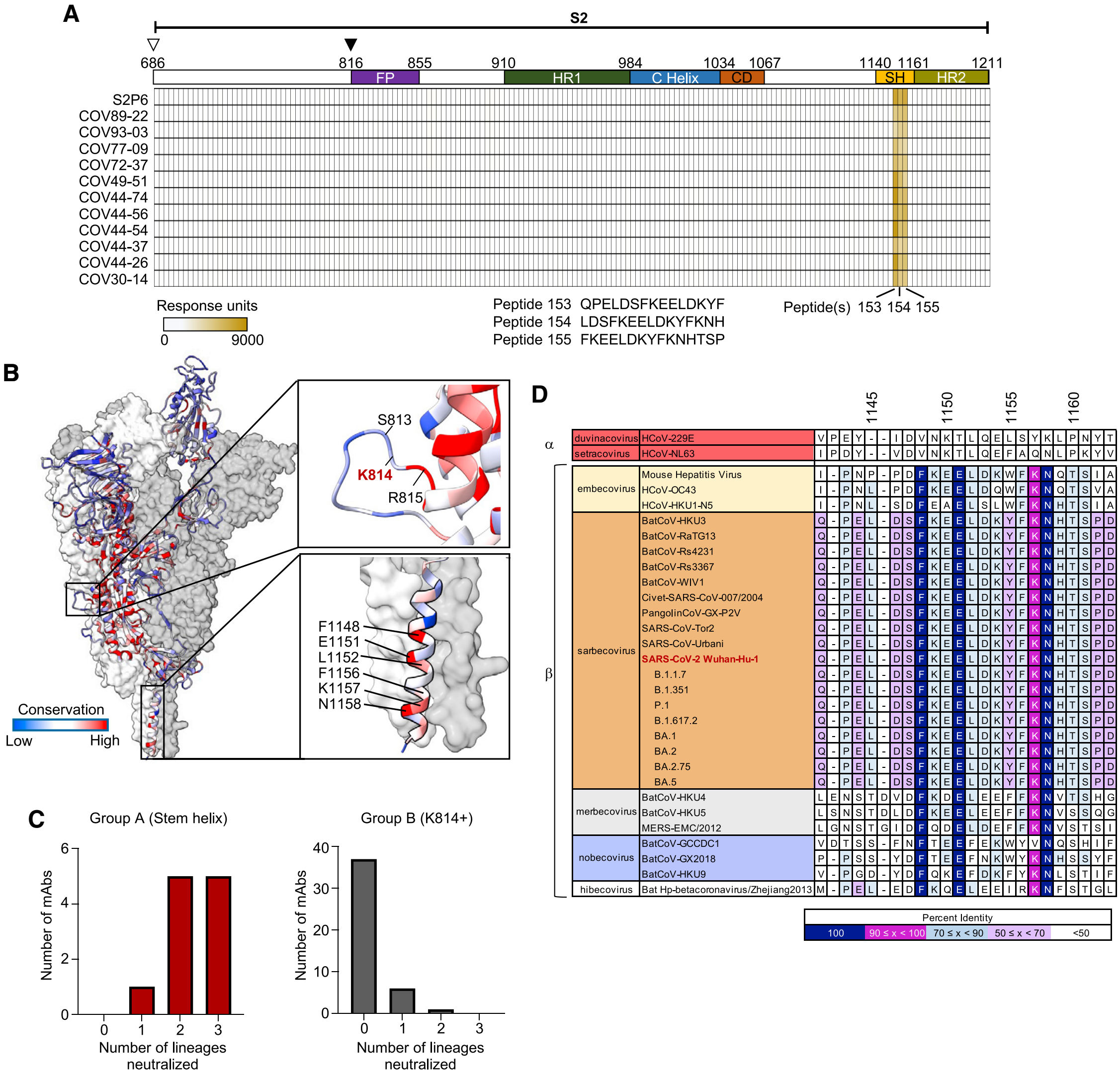

Figure 2. Broadly neutralizing mAbs against betacoronaviruses target the stem helix

(A) Heatmap of SARS-CoV-2 Wuhan-Hu-1 S2 peptide array. SPR was used to measure binding responses to 15-mer peptides (x axis, 3-aa offset) spanning the SARS-CoV-2 Wuhan-Hu-1 S2 subunit. Open triangle indicates the S1/S2 cleavage site, closed triangle indicates the S2′ cleavage site; FP, fusion peptide: HR1, heptad repeat 1; C Helix, central helix; CD, connector domain; SH, stem helix; HR2, heptad repeat 2.

(B) Sequence conservation of native SARS-CoV-2 spike protein (PDB: 7N1Q) using sequence alignment of 28 betacoronaviruses representing each of the 5 subgenera. Insets show K814 and surrounding residues, as well as the stem helix region. Generated using ChimeraX.

(C) Number of betacoronavirus lineages neutralized by group A (stem helix) and group B (K814+) mAbs.

(D) Alignment of stem helix region of betacoronavirus spike proteins using the MAFFT v7.0 software and L-INS-i algorithm. Percent identity of amino acid residues was calculated using only betacoronavirus isolates.

See also Figures S1, S2, and S7.

Group B did not bind to any of the 15-mer peptides (Figure S2A), suggesting that these antibodies recognize a conformational epitope within the S2 subunit. To identify this epitope, we utilized a shotgun mutagenesis approach, wherein S2 subunit residues were individually mutated to alanine in the context of the whole SARS-CoV-2 spike protein to generate a panel of spike mutants. We screened three mAbs in Group B, COV57-19, COV93-18, and COV77-43, against this panel and identified a single amino acid, K814, as critical for binding of all three mAbs (Figure S2B). K814 is located at a poorly characterized site just N-terminal to the S2′ cleavage site and fusion peptide region and is part of a loop that extends to the side of the spike protein (Figure 2B). This residue has also been recently identified as a target of two other SARS-CoV-2 S2-specific mAbs, suggesting that this is a common recognition site (Chen et al., 2021). We named this site K814+, as the epitope recognized by the Group B mAbs most likely encompasses more than K814, but no surrounding amino acid was clearly identified as a target of all three mAbs from this group (Figure S2B).

We compared the neutralization breadth of the Group A and B mAbs to determine the utility of each S2 site as a neutralizing epitope. Strikingly, 10 of 11 of the Group A (stem helix-specific)

CellPress
OPEN ACCESS

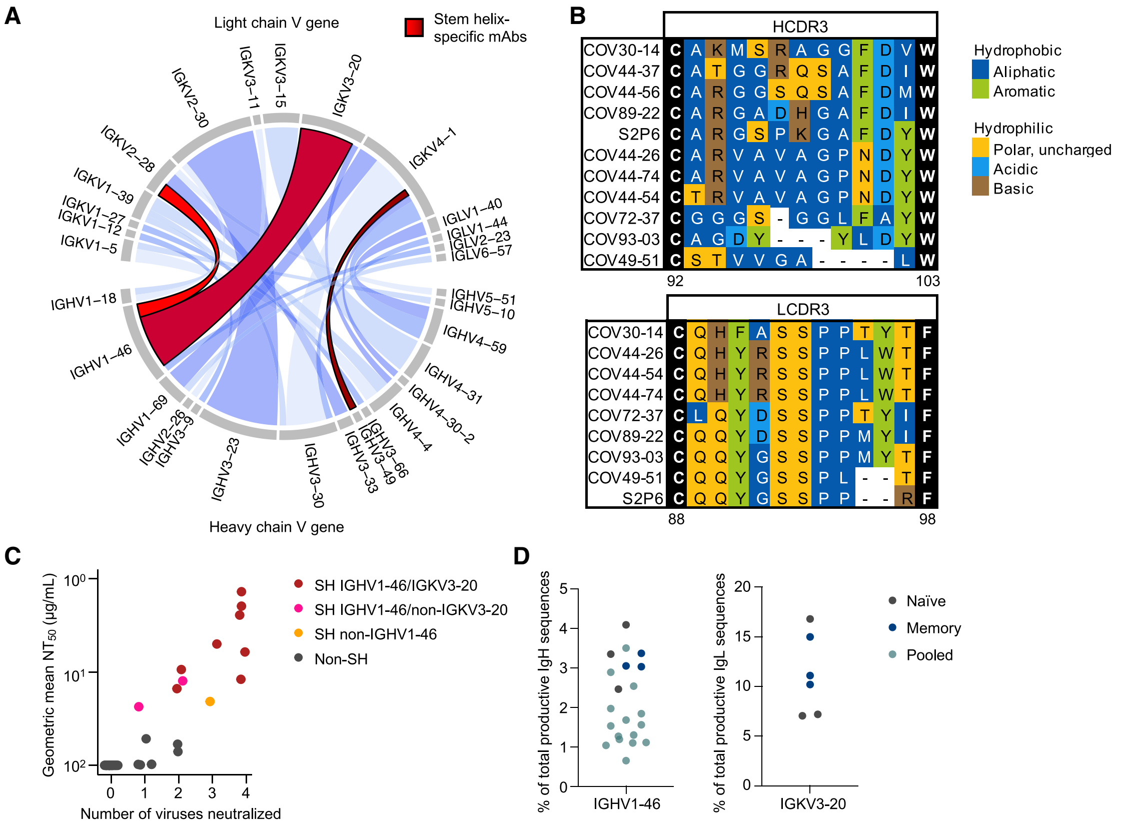

E

		WT	Q (1142)	P	E	L	D	S	**F**	K	E	E	**L**	D	K	Y	**F**	K	N	H	T	S (1161)
IGHV1-46/IGKV3-20	COV89-22	100	100	102	106	100	113	133	174	104	108	92	117	114	69	117	94	64	89	87	96	92
	COV93-03	100	100	100	107	101	111	139	71	145	109	104	155	131	79	146	41	66	84	77	97	88
	COV30-14	100	105	95	100	100	95	108	55	97	97	88	31	113	84	102	64	90	94	94	94	88
	COV72-37	100	105	96	102	102	99	107	81	101	101	89	29	100	89	126	14	93	95	97	96	89
	COV44-26	100	102	99	105	109	76	151	12	87	101	103	27	83	75	61	31	73	93	114	90	89
	COV44-54	100	101	99	104	103	85	148	8	100	103	98	27	85	73	60	36	76	92	112	90	88
	COV44-74	100	102	99	102	101	86	146	7	110	101	93	11	61	77	38	16	81	91	108	91	88
	COV49-51	100	108	89	96	92	76	104	39	100	91	3	1	65	83	2	3	91	79	88	93	79
IGHV1-46/other	COV44-37	100	105	93	103	103	80	105	3	96	98	56	3	74	73	6	1	79	86	99	90	83
	COV44-56	100	108	89	100	100	78	95	5	86	94	48	4	40	79	6	1	87	80	88	91	78
Non-IGHV1-46	COV77-09	100	105	100	104	105	105	136	155	145	97	68	83	107	77	128	5	76	100	124	96	90

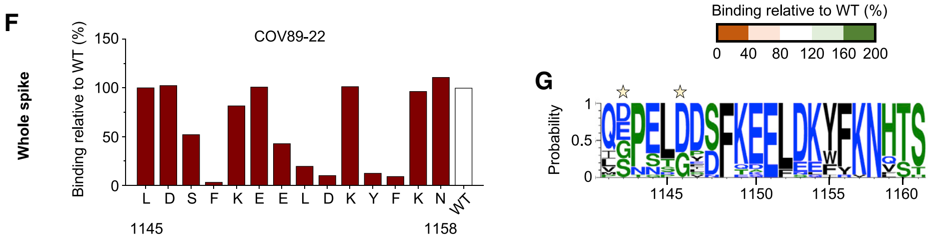

Figure 3. Broadly neutralizing mAbs use IGHV1-46 and target conserved residues on the stem helix

(A) Chord diagram showing paired heavy and light chain V genes used by broadly reactive mAbs against betacoronaviruses. The width of the linkage between a heavy and light chain gene is proportional to the number of mAbs that use the highlighted gene pair.

(B) Alignment of heavy and light chain CDR3 sequences of stem helix-specific mAbs, performed using MAFFT v7.0, G-INS-i algorithm. Only IGHV1-46 mAbs were included in the HCDR3 alignment and only IGKV3-20 mAbs were included in the LCDR3 alignment. Amino acids are colored by chemistry. Black residues indicate conserved residues flanking the CDR3. Amino acid residues are numbered using the Kabat numbering system.

(C) Neutralization potency and breadth of mAbs targeting betacoronaviruses, classified by specificity and V gene usage. The geometric mean NT_{50} was calculated based on neutralization of four betacoronaviruses: SARS-CoV-2, SARS-CoV, MERS-CoV, and HCoV-OC43. Non-neutralizing mAbs were given an NT_{50} value of 100 µg/mL. SH, stem helix.

(D) Percentage of B cells using IGHV1-46 and IGKV3-20 genes in healthy donors. Values were obtained from next-generation B cell receptor sequencing datasets in the iReceptor database (https://gateway.ireceptor.org/).

(E) Alanine scan of stem helix peptide. Key binding residues for the majority of mAbs, F1148, L1152, and F1156, are shown in bold.

(legend continued on next page)

mAbs were capable of neutralizing at least two different betacoronavirus lineages (Figure 2C). Moreover, the broadest neutralizing mAbs isolated in this study (COV89-22, COV30-14, COV72-37, COV44-26, and COV44-74) all belonged to Group A. In contrast, the majority of Group B mAbs did not neutralize a single betacoronavirus, and only a single mAb from this group cross-neutralized coronaviruses from two different lineages. These findings suggest that the majority of broadly reactive mAbs against betacoronaviruses target the K814+ site and are poorly neutralizing, whereas a minority target the stem helix and are capable of broadly neutralizing betacoronaviruses.

Therefore, we decided to focus our efforts on further characterizing the Group A, stem helix-specific mAbs. We performed a sequence alignment of 28 isolates representing the five betacoronavirus subgenera to determine the degree of conservation of the stem helix sequence among betacoronaviruses (Figures 2B and 2D). In particular, amino acids F1148, E1151, K1157, and N1158 within the stem helix are highly conserved (>90%) within the betacoronavirus subgenera, which is consistent with the breadth observed in the stem helix-specific mAbs (Figure 2C). All SARS-CoV-2 variants of concern identified to date, including the Omicron subvariant BA.5, have identical sequences in this region. The human alphacoronaviruses HCoV-229E and HCoV-NL63 have divergent sequences at this location, which explains the lack of binding and neutralization of the Group A mAbs to these viruses (Figures 1 and 2D).

Stem helix-specific mAbs from multiple donors use an IGHV1-46/IGKV3-20 gene signature

To investigate the genetic profile of the stem helix-specific mAbs, we examined their heavy and light chain V gene usage, as well as their complementarity-determining region 3 (CDR3) amino acid sequences. Interestingly, 10 of 11 mAbs targeting the stem helix used an IGHV1-46 heavy chain (Figure 3A). In eight mAbs, this heavy chain was paired with an IGKV3-20 light chain. Of the remaining 44 broadly reactive mAbs, only one used IGHV1-46 and a different mAb used IGKV3-20. This V gene preference was not due to the expansion of a single B cell clone or V gene bias from a single donor, as the 10 mAbs were isolated from six different donors and only three (COV44-26, COV44-54, and COV44-74) were clonally related. The VH nucleotide mutation levels of the IGHV1-46 stem helix mAbs were between 4.8% and 12.2%, and the VH amino acid mutations were between 9.3% and 21.4%, indicative of prior experience in a germinal center (Figure S3A). A comparison of the heavy chain CDR3 sequences of the IGHV1-46 mAbs revealed that this group of mAbs had divergent HCDR3 sequences, supporting a role for IGHV1-46-specific elements, such as HCDR1 and HCDR2, in binding to the stem helix (Figure 3B). The light chain CDR3s of the IGHV1-46/IGKV3-20 mAbs were more similar (Figure 3B), but this was unsurprising, given the large contribution of IGKV3-20 residues to this region.

Next, we compared the potency and breadth of the IGHV1-46/IGKV3-20 mAbs to the other broadly reactive mAbs. Notably, the six most potent and broadly neutralizing mAbs in our panel used the IGHV1-46/IGKV3-20 combination, and all eight mAbs in this group neutralized at least two betacoronaviruses (Figure 3C). These findings suggest that the ability to produce IGHV1-46/IGKV3-20 mAbs is advantageous for immune defense against betacoronaviruses. To determine the frequency of B cells using these V genes, we used the iReceptor database (https://gateway.ireceptor.org/) (Corrie et al., 2018) to screen published next-generation B cell receptor sequencing datasets from healthy individuals where at least 1 million rearranged sequences were obtained (Briney et al., 2019; DeKosky et al., 2015, 2016; DeWitt et al., 2016; Tipton et al., 2015). At least 0.65% of B cells (average 2.1%) in each individual (n = 21) used IGHV1-46 (Figure 3D). Only six donors met our criteria for VL gene analysis, but at least 7.05% (average 11.2%) of B cells in each donor used IGKV3-20 (Figure 3D). Furthermore, a separate study that performed deep sequencing of VK genes in four individuals (Jackson et al., 2012) found that IGKV3-20 was the most common kappa gene in all donors. Collectively, these findings suggest that IGHV1-46 and IGKV3-20 are commonly used individually by B cells in healthy individuals, although their combination would have a lower probability.

Of the eight IGHV1-46/IGKV3-20 stem helix-specific mAbs, COV44-26, COV44-54, and COV44-74 belonged to the same clonal lineage, allowing us to investigate the effects of affinity maturation on binding to the stem helix. We evaluated the binding of the putative unmutated common ancestor (UCA) and intermediates of this lineage to the SARS-CoV-2 spike protein and peptide 154 from the stem helix (Figures S3B and S3C). The UCA bound well to the stem helix peptide and was able to bind to the SARS-CoV-2 spike protein, albeit more weakly than all the other members of the lineage, which bound similarly to the spike and stem helix. When comparing the sequences of these mAbs, the HCDR2 stood out as a region where the UCA was substantially different from the other members of the clonal lineage (Figure S3D). We also produced VJ germline-reverted versions of the potent IGHV1-46/IGKV3-20 mAbs COV89-22, COV30-14, and COV72-37 and compared the characteristics of the germline and mature versions of these mAbs (Figures S3E and S3F). The germline mAbs were capable of binding the SARS-CoV-2 stem helix and betacoronavirus spikes (with the exception of the COV72-37 and COV89-22 germlines with the MERS-CoV spike) but were mostly non-neutralizing. In contrast, the mature forms of the mAbs were superior in both binding and neutralization. Taken together, these results suggest that naive B cells carrying IGHV1-46/IGKV3-20 are capable of engaging the stem helix, but somatic mutations increase both binding and neutralization potency of this class of mAbs.

We conducted an alanine scan on the stem helix peptide to determine whether the IGHV1-46/IGKV3-20 mAbs preferentially

(F) Effect of mutations on COV89-22 binding in a shotgun mutagenesis assay. A residue was considered critical if mutation of this residue resulted in a reduction of binding signal for COV89-22 but not control mAb C, which targets a linear epitope not in this region (see Figure S3H).
(G) Sequence logo plot of spike protein from 28 aligned betacoronavirus isolates representing each of the 5 subgenera. Amino acid residues are colored by hydrophobicity. Numbering is based on the SARS-CoV-2 Wuhan-Hu-1 sequence. Yellow stars indicate amino acids absent in SARS-CoV-2 Wuhan-Hu-1 Spike protein but present in at least one other sequence used for the alignment. Created using Weblogo3.0.
See also Figure S3.

CellPress
OPEN ACCESS

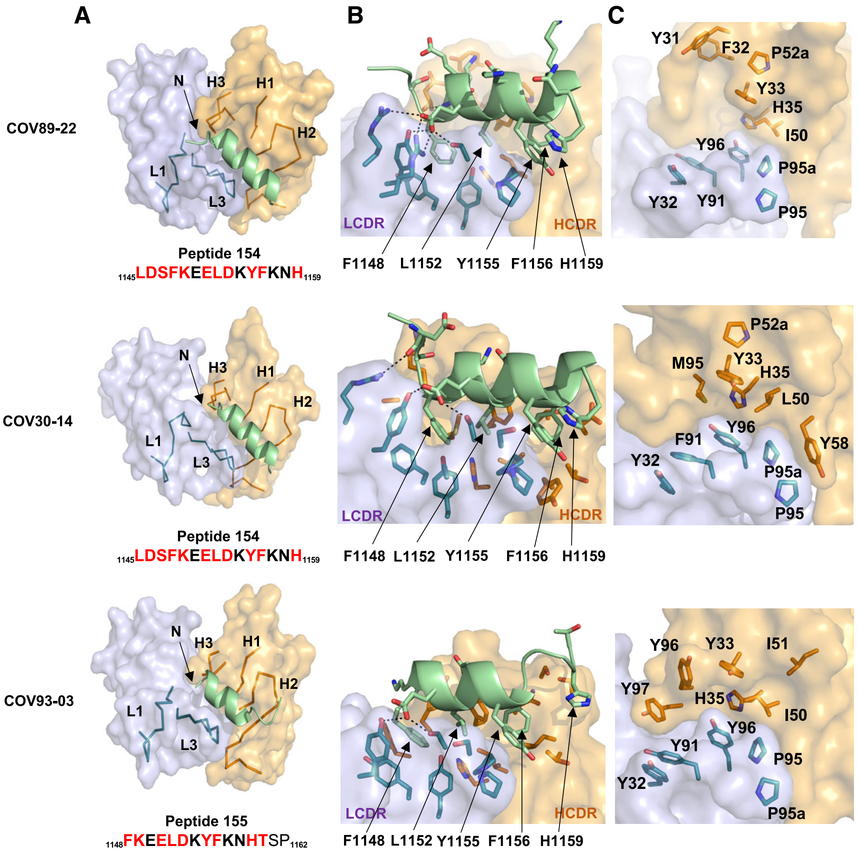

Figure 4. Crystal structure of COV89-22, COV30-14, and COV93-03 in complex with SARS-CoV-2 stem helix peptide

(A) Overall interactions of COV89-22, COV30-14, and COV93-03 with the stem helix peptide. Fabs are shown in a molecular surface and the complementarity-determining regions (CDRs) and peptide are represented as backbone lines and ribbons, respectively. Orange and light blue represent the heavy and light chains of the Fabs. Peptides are shown in green. H1, H2, H3, L1, and L3 represent CDRs in the heavy (H) and light (L) chains. The resolution of the three crystal structures are 1.6, 1.5, and 1.75 Å. Peptide residues observed in the electron density maps are in bold and residues involved in interaction with antibody (BSA > 0 Å^2) are in red.

(B) Molecular details of COV89-22, COV30-14, and COV93-03 and S2 stem helix peptide interaction. Arrows indicate hydrophobic and aromatic residues of the peptides involved in interaction with COV89-22, COV30-14, and COV93-03.

(C) Hydrophobic and aromatic residues in the binding pocket of COV89-22, COV30-14, and COV93-03. Fab residues are in Kabat numbering. See also Figures S4–S6 and Table S1.

formed contacts with a distinct set of amino acids from the other mAbs targeting this site (Figure 3E). We also used the spike shotgun mutagenesis assay to further examine the binding profile of COV89-22, a high-affinity binder and the most potent mAb in our panel (Figures 3F, S3G, and S3H), since it was less susceptible to mutations in the context of the stem helix peptide. There was no clear difference between the binding profiles of COV44-74 and COV49-51, which use IGKV3-20, and COV44-37 and COV44-56, which use IGKV2-28, suggesting that the light chains were more permissive for specificity of these mAbs toward the stem helix. Overall, residues F1148, L1152, and F1156 were important for the majority of IGHV1-46 mAbs, whereas the sole non-IGHV1-46 mAb, COV77-09, only required F1156. F1148 is conserved in all betacoronavirus sequences examined, whereas L1152 and F1156 are conserved in >80% of the sequences (and with similar amino acid types as mutations) (Figures 2D and 3G). When we examined SARS-CoV-2 spike sequences from the GISAID database (https://gisaid.org/) (Elbe and Buckland-Merrett, 2017) for mutation frequencies at these positions, we found that mutations at F1148, L1152, and F1156 were only present in 0.0002%, 0.0004%, and 0.002% of all sequences, respectively. To determine the effects of mutations at these positions on virus fitness, we produced pseudoviruses carrying single F1148A, L1152A, or F1156A mutations, or a triple F1148A/L1152A/F1156A mutation, in parallel with wild-type (WT) pseudovirus produced at the same time using the same protocol. We compared the infectivity of each undiluted pseudovirus preparation and observed a clear reduction in the infectivity of all mutants (Figure S3I). To determine if this was due to a defect in pseudovirus production (e.g., due to spike misfolding) or the infective capacity of intact virions (or both), we quantified the p24 antigen concentration of each preparation. There was a clear reduction in p24 concentration for all mutants (Figure S3J), and when the infectivity was normalized based on this count, only F1148A and the triple mutant showed substantially reduced function (Figure S3K). Collectively, the data indicate that a mutation at each of the three positions (F1148, L1152, and F1156) impairs virus production, with F1148A further reducing the infectivity of the virions that are produced.

Crystal structure of three IGHV1-46 stem antibodies in complex with the stem helix peptide

To decipher how COV89-22, COV30-14, and COV93-03 interact with the S2 stem helix in neutralizing SARS-CoV-2, the Fabs of these three antibodies were complexed with the 15-mer peptides 154 or 155, which cover the stem helix region (Figures 2A and 4). The crystal structures of the COV89-22/COV30-14/COV93-03-peptide complexes were determined at 1.6, 1.5, and 1.75-Å resolution, respectively (Figures 4 and S4; Table S1). All residues of peptide 154 and thirteen of fifteen residues of peptide 155 were visible in the electron density maps (Figure S4A). Eleven residues of both peptides had a buried surface area (BSA) > 0 Å^2 in the interface with antibody (Figure S5A). These antibodies share the same IGHV and IGKV germlines (IGHV1-46/IGKV3-20) as another anti-stem helix antibody S2P6 (Pinto et al., 2021). They contain 15/12/12 a.a. somatic mutations in the heavy chain and 8/11/5 a.a. in the light chain variable regions (VH/VL) of COV89-22/COV30-14/COV93-03,

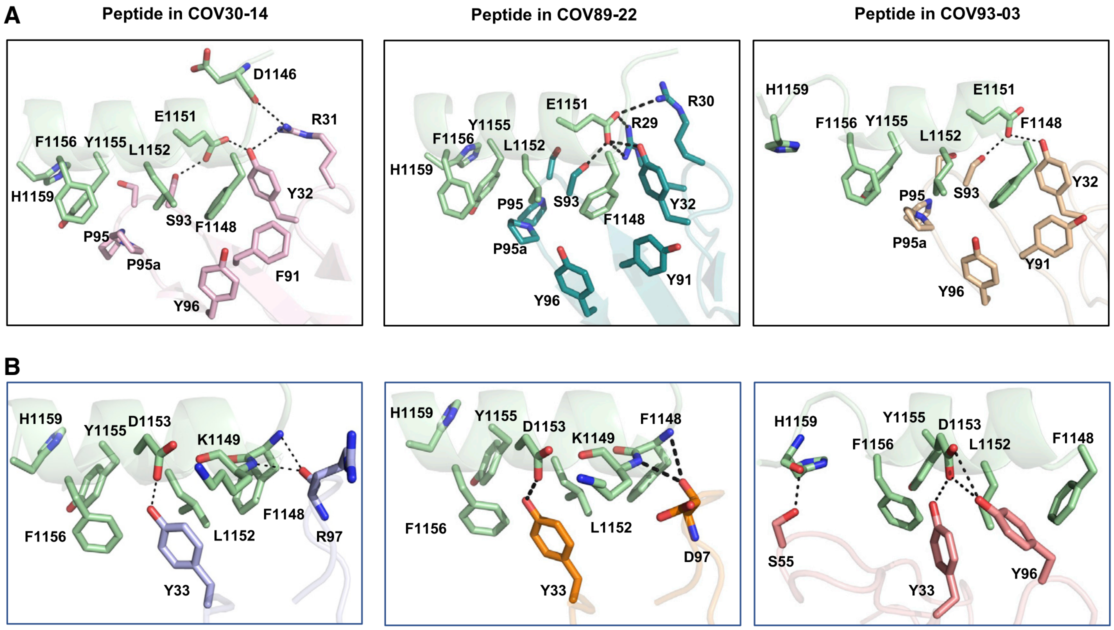

Figure 5. Comparison of interactions of COV30-14, COV89-22, and COV93-03 with SARS-CoV-2 stem helix peptide

(A and B) Similarity and differences in the interactions of (A) light chain and (B) heavy chain among COV30-14, COV89-22, and COV93-03. Fabs and peptide are shown in backbone cartoons with interacting side chains in sticks. Stem helix peptides in each complex are shown in green. Light and heavy chain in COV30-14-Fab-peptide complex are presented in pink and lavender, respectively. Teal and orange are used in COV89-22-Fab-peptide complex. Wheat and light red are used in COV93-03-Fab-peptide complex. H-bonds and salt bridges are indicated with black dashes. Side chains involved in interactions are shown as sticks. Fab residues are in Kabat numbering.

See also Figures S4–S6 and Table S1.

respectively (Figures S6A and S6B). These three antibodies contact the stem helix peptide via CDR1, CDR2, and CDR3 in the heavy chain and CDR1 and CDR3 in the light chain (Figure 4A). The BSA of each residue of the stem helix peptide exhibited a similar distribution among COV89-22, COV30-14, and COV93-03 (Figure S5A). Furthermore, the molecular surface contact area reveal that they share very similar contact patterns for the main chain and side chain of the peptides among COV89-22/COV30-14/COV93-03 (Figure S5B). Among the three Fab-peptide complexes, F1148, L1152, Y1155, F1156, and H1159 of the peptide make hydrophobic interactions with a largely hydrophobic groove in the antibody composed of common residues from the heavy and light chains (Figures 4B, 4C, and S5C). The aromatic residues, Y/H91-Y96 motif in LCDR3 and Y32 in LCDR1, create a hydrophobic cavity to accommodate F1148, L1152, and Y1155, consistent with the substantial loss of binding with Ala mutations in the spike protein (Figures 3E and 3F). Furthermore, the **RR**N**Y** residues (29–32) of LCDR1 along with S93 in LCDR3 in COV89-22 form a network of H-bonds and salt bridges with E1151 of the stem helix peptide (Figure 5A) that accounts for a decrease in binding to E1151A in the spike protein (Figure 3F). The equivalent TG**RY** and TSN**Y** residues of LCDR1 in COV30-14 and COV93-03 contribute H-bonds but no direct salt bridges with stem helix peptide (Figure 5A). In addition, D1153 of the stem helix peptide hydrogen bonds with Y33 and also forms backbone-backbone interactions between residues 1148 and 1149 with residue 97 in HCDR1 and HCDR3 in COV89-22 and COV30-14 (Figure 5B). In COV93-03, the Y96 sidechain replaces the residue 97 interaction but here contributes two H-bonds with D1153. HCDR3 Y96 also enhances hydrophobic and aromatic interactions among Y33, L1152, Y1155, and F1156. These findings suggest that all known IGHV1-46/IGKV3-20 antibodies mainly target the region F1148 to F1156 of the SARS-CoV-2 stem helix with a highly similar binding mode, and the key residues are consistent with those identified by the Ala scanning of the spike protein. Notably, IGHV1-46 encodes residues that contact the stem helix including the CDR2 I50, whereas IGKV3-20 (and the closely related IGKV3D-20) encodes the YGSSP motif, which includes key contact residues, consistent with the frequent use of these genes by stem helix-specific mAbs (Figure S6C). In complex with the antibodies here, the monomeric stem helix peptide forms a helix as observed in both pre-fusion and post-fusion states of the SARS-CoV-2 spike (Figures S4B and S4C). However, antibody binding to this region would clash with the three-helix bundle in the stem region in the pre-fusion state and in the post-fusion state, which suggests that binding to the spike requires a conformational change or increased dynamics from its pre-fusion form to a more open state, perhaps along a trajectory toward its post-fusion form.

Response to stem helix following vaccination and infection

To investigate antibody responses to the stem helix after COVID-19 vaccination or SARS-CoV-2 infection, we screened polyclonal IgG isolated from serum or plasma from the following donors for binding to peptide 154 from the stem helix region: individuals vaccinated with mRNA-1273 (Moderna)

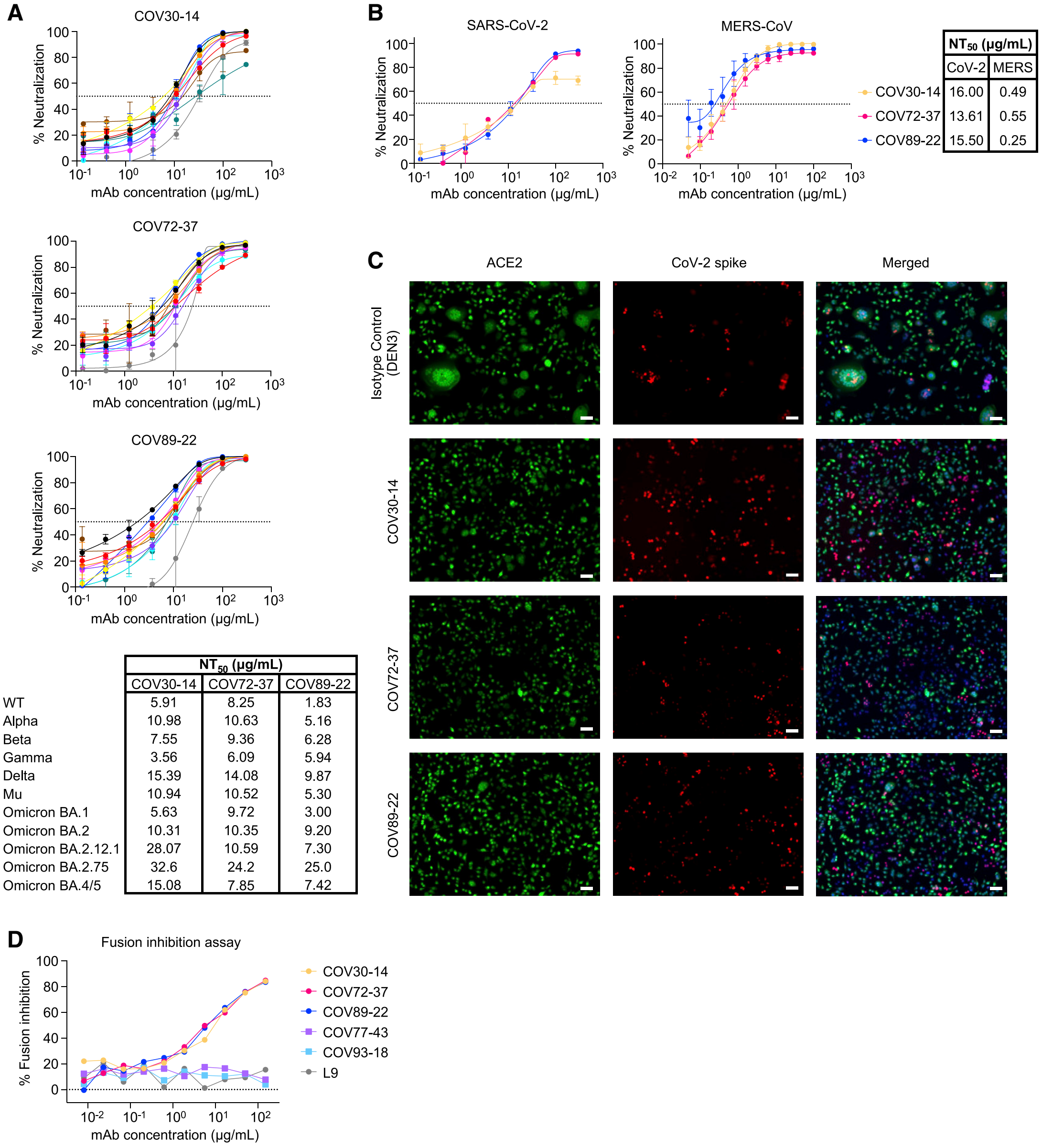

	NT50 (μg/mL)		
	COV30-14	COV72-37	COV89-22
WT	5.91	8.25	1.83
Alpha	10.98	10.63	5.16
Beta	7.55	9.36	6.28
Gamma	3.56	6.09	5.94
Delta	15.39	14.08	9.87
Mu	10.94	10.52	5.30
Omicron BA.1	5.63	9.72	3.00
Omicron BA.2	10.31	10.35	9.20
Omicron BA.2.12.1	28.07	10.59	7.30
Omicron BA.2.75	32.6	24.2	25.0
Omicron BA.4/5	15.08	7.85	7.42

	NT50 (μg/mL)	
	CoV-2	MERS
COV30-14	16.00	0.49
COV72-37	13.61	0.55
COV89-22	15.50	0.25

Figure 6. COV30-14, COV72-37, and COV89-22 neutralize SARS-CoV-2 variants of concern and inhibit SARS-CoV-2 spike-mediated fusion
(A) Neutralization of SARS-CoV-2 variants of concern (pseudovirus) by COV30-14, COV72-37, and COV89-22. The dotted line indicates 50% neutralization. Error bars show mean ± SD.
(B) Neutralization of authentic SARS-CoV-2 Wuhan-Hu-1 and MERS-CoV by COV30-14, COV72-37, and COV89-22. The dotted line indicates 50% neutralization. Error bars show mean ± SD.
(C) Representative images of syncytia formation in co-cultures of SARS-CoV-2 spike expressing HeLa cells (RFP) and ACE2 receptor expressing HeLa cells (GFP) counter-stained with Hoechst (blue). Anti-dengue NS1 human IgG1 mAb DEN3 was included as an isotype control. Scale bars, 100 μm.
(D) Fusion inhibition of stem helix-specific mAbs (COV30-14, COV72-37, and COV89-22) and K814+-specific mAbs (COV77-43 and COV93-18) in a quantitative assay. The malaria mAb L9 (Wang et al., 2020) was used as a negative control.
See also Table S2.

(Figure S7A), unvaccinated donors recovering from a recent SARS-CoV-2 infection, and unvaccinated COVID-19-naive donors. COVID-19-naive individuals had negligible antibodies to this peptide, indicating a minimal contribution from previous infections by seasonal betacoronaviruses such as HCoV-HKU1 and HCoV-OC43 (Figure S7B). There was an increase in the level of stem helix-specific antibodies after the second vaccination ($p < 0.001$), but this rapidly declined and was not restored by the booster dose (Figure S7B). As a group, the convalescent individuals had higher responses than the naive donors ($p = 0.0049$) but did not have higher responses than the vaccinated individuals ($p = 1$). Overall, vaccination with mRNA-1273 and natural infection did not induce high levels of antibodies against the stem helix region.

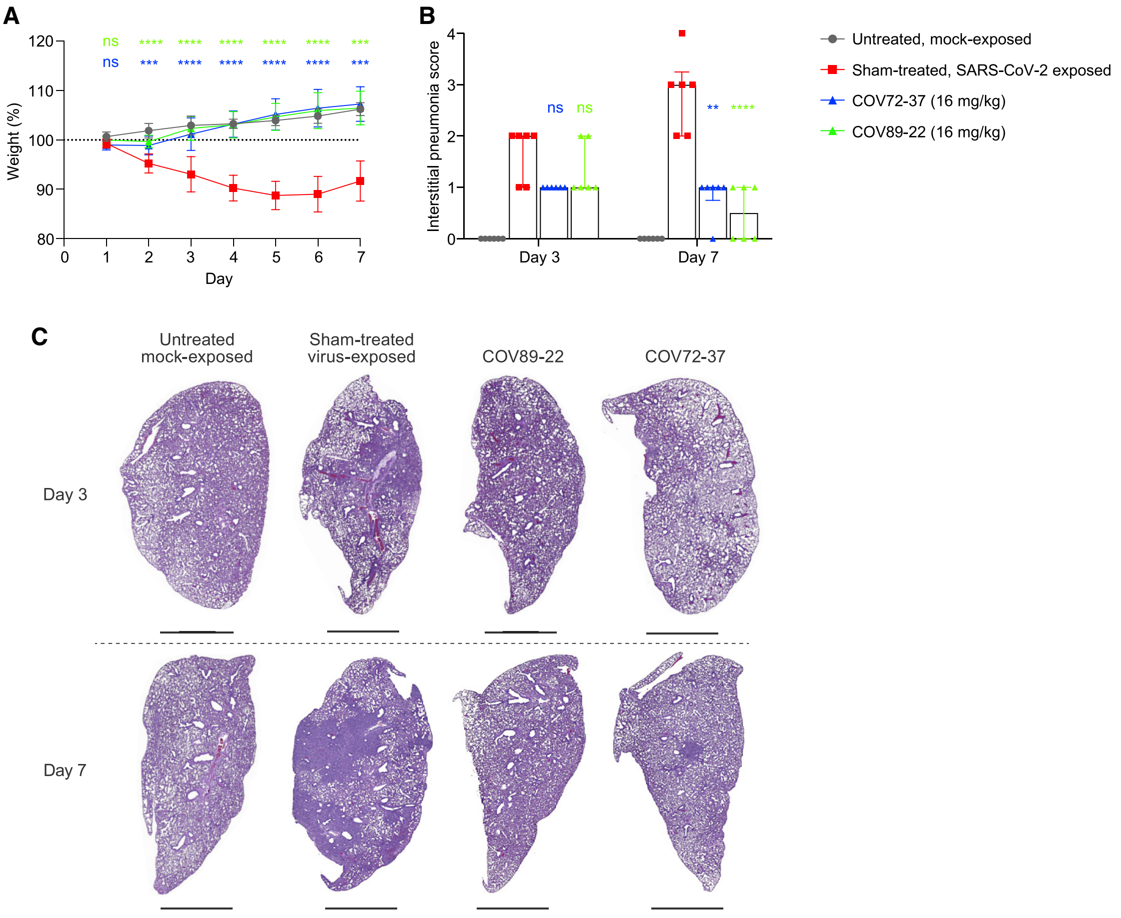

Figure 7. COV72-37 and COV89-22 limit SARS-CoV-2-mediated disease in Syrian hamsters

(A and B) Clinical outcomes of SARS-CoV-2 exposed Syrian hamsters after prophylaxis with stem helix mAbs. (A) Weight change was assessed using a mixed-effects repeated measures model with Dunnett's post-test multiple comparison (n = 12 animals from days 0–3 and n = 6 animals from days 4–7), and error bars represent mean ± SD. (B) Pneumonia pathology distribution scores on days 3 and 7 were analyzed by a Kruskal-Wallis test with Dunn's post-test multiple comparison (n = 6–12 animals per condition), between the mAb-treated and mock-treated groups on each day. Bars show median ± interquartile range. * $p < 0.05$, ** $p < 0.01$, *** $p < 0.001$, **** $p < 0.0001$ and ns, not significant.

(C) Images of sagittal sections of the left lung lobe from untreated Syrian hamsters and those administered COV89-22 and COV72-37. Scale bars, 4 mm.

Stem helix-specific mAbs neutralize SARS-CoV-2 variants of concern and inhibit fusion

We tested three of the most potent stem helix-specific mAbs, COV89-22, COV30-14, and COV72-37, for their ability to neutralize SARS-CoV-2 variants of concern. The three mAbs neutralized all variants tested including Omicron BA.4/5 (Figure 6A), which is consistent with the identical sequence of this region in all variants of concern (Figure 2D). We also confirmed that these mAbs neutralized authentic SARS-CoV-2 and MERS-CoV, as well as a panel of betacoronaviruses in a second pseudovirus assay (Figure 6B; Table S2). We then proceeded to test if these mAbs inhibit fusion of cells expressing SARS-CoV-2 spike protein and cells expressing ACE2, which is a potential mechanism of action of stem helix-specific mAbs (Li et al., 2022; Pinto et al., 2021; Wang et al., 2021). We found that COV89-22, COV30-14, and COV72-37 inhibited fusion in both an imaging-based and quantitative assay, wherein fusion results in the release of an enzyme that cleaves a chromogenic substrate (Figures 6C and 6D).

COV89-22 and COV72-37 limit disease in SARS-CoV-2-infected Syrian hamsters

We tested COV89-22 and COV72-37 for the ability to limit disease in the Syrian hamster model of SARS-CoV-2 infection (Figure 7). To allow for optimal Fc function, we converted the Fc regions of the two mAbs to hamster IgG2. Each mAb was administered intraperitoneally at a 16-mg/kg dose, followed by intranasal infection with 10^5 plaque-forming units (PFU) of SARS-CoV-2 one day later. Disease progression in the hamsters (n = 12 per group) was monitored through daily assessment of changes in body weight, as well as histopathology measurements on days 3 and 7. As expected from previous studies, untreated hamsters lost around 10% of body weight through day 6 post-infection (Figure 7A) (Cho et al., 2021). In contrast, hamsters treated with COV89-22 and COV72-37 maintained body weight similar to the uninfected controls throughout the study ($p < 0.001$ relative to untreated hamsters from days 2–7 for both mAbs). Most hamsters in the COV89-22 and COV72-37 groups showed only mild signs of interstitial pneumonia

based on histopathological examination of lung tissue (median score 1 or less), consistent with the body weight data (Figures 7B and 7C). Hamsters treated with COV89-22 showed a reduced pathology score from days 3 to 7, whereas the control group worsened during this period (Figure 7B). Collectively, these findings suggest that COV89-22 and COV72-37 are effective in limiting disease in this model of SARS-CoV-2 infection.

DISCUSSION

In this study, we identify convergent IGHV1-46/IGKV3-20 mAbs from several individuals that target the S2 stem helix for broad neutralization of betacoronaviruses. Betacoronavirus-neutralizing mAbs targeting this region have previously been identified, including the IGHV1-46/IGKV3-20 mAb S2P6 (Pinto et al., 2021), affirming the importance of this region as a target site of neutralizing antibodies (Li et al., 2022; Pinto et al., 2021; Sauer et al., 2021; Wang et al., 2021; Zhou et al., 2022a, 2022b). The favored use of specific V genes across multiple individuals has previously been observed for mAbs against other infectious disease targets such as the influenza hemagglutinin stem (IGHV1-69) (Chen et al., 2019), *Plasmodium falciparum* circumsporozoite protein (IGHV3-30/IGHV3-33) (Tan et al., 2018; Murugan et al., 2018), HIV-1 gp120 CD4 binding site (IGHV1-2/IGHV1-46) (Scheid et al., 2011; Wu et al., 2011), and SARS-CoV-2 RBD (IGHV3-53/IGHV3-66) (Barnes et al., 2020; Yuan et al., 2020a). Here, the IGHV1-46/IGKV3-20 combination is relatively uncommon in the wider mAb panel as it is not used by any of the K814+-specific mAbs, which constitute the majority of the mAbs described in this study. However, IGHV1-46/IGKV3-20 is used very frequently (72.7%) by mAbs targeting the stem helix, suggesting positive selection due to favorable binding to the stem helix (Zhou et al., 2022a). Accordingly, we found that VJ germline-reverted versions of these mAbs are capable of binding to the stem helix, suggesting that naive B cells using IGHV1-46/IGKV3-20 already have the ability to target this epitope. Nevertheless, both binding and neutralization are improved with somatic mutations, highlighting the importance of the germinal center reaction in enhancing the antibody response to this site.

This study provides information that could be useful for the design of next-generation coronavirus vaccines. The continuous emergence of new SARS-CoV-2 variants of concern that evade neutralizing antibody responses has provided strong motivation to develop vaccines that target more conserved regions of the spike protein. The S2 subunit, which is more conserved than S1, is currently being explored as a candidate for this purpose (Shah et al., 2021). However, these data suggest that the K814+ site in S2 is immunodominant and triggers broadly reactive but not broadly neutralizing antibodies. In contrast, the stem helix elicits fewer antibodies, perhaps due to limited accessibility in the pre-fusion spike but is a better target for eliciting neutralizing antibodies. Of a panel of 55 broadly reactive mAbs toward betacoronaviruses, the 11 mAbs that targeted the stem helix were also the 11 most potent mAbs based on the average NT_{50} value against a panel of four betacoronaviruses. Therefore, a targeted construct that focuses the immune response on the stem helix and avoids the immunodominant K814+ site or an S2 construct that masks this site may be promising for design of a broad betacoronavirus vaccine. Moreover, the crystal structure and mutagenesis data show the precise binding mode of potent stem helix-specific mAbs and identify key stem helix residues that must be included in the vaccine construct to elicit the desired antibody response. The conserved mode of interaction of the IGHV1-46/IGKV3-20 mAbs with the stem helix can serve as a template for the design of germline-targeting immunogens that aim to activate these B cell lineages.

The major drawback of mAbs targeting the stem helix is their lower *in vitro* neutralization potency relative to the RBD-specific mAbs that have been developed as clinical products (Dougan et al., 2021; Gupta et al., 2021; Weinreich et al., 2021; Takashita et al., 2022a). However, the stem helix-specific mAbs are more likely to retain function against new SARS-CoV-2 variants than mAbs targeting the RBD, which has shown the ability to accumulate diverse mutations without substantial or any loss of binding to ACE2 (Starr et al., 2020). Furthermore, *in vitro* potency does not always reflect efficacy in humans, as other factors such as Fc activity also contribute to protection (Bartsch et al., 2021). For instance, the therapeutic mAb sotrovimab has lower *in vitro* potency than most other therapeutic mAbs in the clinic but showed similar efficacy in preventing progression to severe COVID-19 disease in humans (Dougan et al., 2021; Gupta et al., 2021; Weinreich et al., 2021; Takashita et al., 2022a). The stem helix-specific mAbs described here, in particular COV89-22, were effective in preventing disease mediated by SARS-CoV-2 in a hamster model. Therefore, stem helix-specific mAbs and vaccine constructs should be further explored as countermeasures that could be immediately utilized for protection from future SARS-CoV-2 variants or novel betacoronaviruses.

Limitations of the study

As mentioned above, the stem helix-specific mAbs described here have lower potency than potent RBD-specific mAbs, which have NT_{50} values in the ng/mL range. These mAbs will have to be further characterized to determine whether they are potent enough to be used to prevent COVID-19 or reduce the risk of progression to severe disease in humans. Furthermore, we only evaluated the *in vivo* efficacy of these mAbs against SARS-CoV-2, and it is unclear if they also function *in vivo* against other betacoronaviruses, although we note that previously described mAbs with similar specificity showed *in vivo* function against MERS-CoV (Wang et al., 2021; Zhou et al., 2022a). Although these data are useful for vaccine design, we have not performed vaccination experiments in this study and thus cannot draw any definitive conclusions with regard to the efficacy of stem helix-based vaccines. Whether a stem helix-based vaccine can elicit sufficient antibody titers to neutralize betacoronaviruses in humans remains to be investigated. Although the stem helix is well conserved in betacoronaviruses and has an identical sequence in all SARS-CoV-2 variants of concern, it cannot be guaranteed that a new variant of concern with mutations in this region will not emerge in the future. This will have to be closely monitored.

STAR★METHODS

Detailed methods are provided in the online version of this paper and include the following:

- KEY RESOURCES TABLE

- RESOURCE AVAILABILITY
  - Lead contact
  - Materials availability
  - Data and code availability
- EXPERIMENTAL MODEL AND SUBJECT DETAILS
  - Study cohort
  - Cell culture
  - Viruses
  - Hamster model
- METHOD DETAILS
  - Coronavirus spike proteins
  - Generation of multiplexed CoV antigen beads
  - Sequence alignment of coronaviruses
  - Memory B cell isolation from PBMCs
  - Optofluidic-based isolation of B cells
  - mAb sequence analysis and expression
  - HLA typing of donor cDNA
  - mAb binding to coronavirus antigens
  - V gene usage survey
  - Epitope binning by SPR
  - SARS-CoV-2 S2 binding kinetics
  - SARS-CoV-2 S2 peptide mapping
  - Fab expression for crystallization
  - Crystallization and structural determination
  - Microscopy-based fusion inhibition assay
  - Quantitative fusion inhibition assay
  - Shotgun mutagenesis epitope mapping
  - Authentic HCoV-OC43-GFP neutralization assay
  - Pseudovirus neutralization assay (Assay$_{NIH}$)
  - Pseudovirus neutralization assay (Assay$_{Scripps}$)
  - Spike mutant generation and infectivity assay
  - Authentic SARS-CoV-2 neutralization assay
  - Authentic MERS-CoV neutralization assay
  - Hamsterization of human monoclonal antibodies
  - Syrian hamster efficacy studies
  - Vaccinee and convalescent donor IgG binding
- QUANTIFICATION AND STATISTICAL ANALYSIS

SUPPLEMENTAL INFORMATION

Supplemental information can be found online at https://doi.org/10.1016/j.chom.2022.10.010.

ACKNOWLEDGMENTS

We thank the blood sample donors at the New York Blood Center; Sandhya Bangaru, Gabriel Ozorowski, Alba Torrents de la Peña, and Andrew Ward for providing HCoV-OC43 and MERS-CoV spike; Henry Tien for technical support with automated crystalization; Jeanne Matteson for contributions to mammalian cell culture; Robyn Stanfield for technical assistance; Gavin Wright and Nicole Muller-Sienerth (Wright lab, University of York) for providing recombinant CD4; Melanie Cohen and Julie Laux for assistance with cell sorting; Walter Hardy for assistance in screening the Ala scan library; the staff of Stanford Synchrotron Radiation Laboratory (SSRL) Beamline 12–1 and the Advanced Photon Source (APS) Beamline 23-ID-B for assistance. We thank Nick Vaughan, Kurt Cooper, Becky Reeder, Marisa St Claire, Kyra Hadley, Amanda Hischak, Randy Hart, and Nejra Isic for assistance with hamster experiments. We thank Jiro Wada for assistance with graphics for histopathology lung sections. We gratefully acknowledge all data contributors, i.e., the authors and their Originating laboratories responsible for obtaining the specimens, and their Submitting laboratories for generating the genetic sequence and metadata and sharing via the GISAID Initiative. This work was supported by the Division of Intramural Research and the Vaccine Research Center, National Institute of Allergy and Infectious Diseases (NIAID), National Institutes of Health (NIH) (I.D., S.M., J.W.Y., P.D.C., M.R.H., J.R.M., and J.T.). This project has been funded in whole or in part with Federal funds from the National Institute of Allergy and Infectious Diseases (NIAID), National Institutes of Health (NIH), U.S. Department of Health and Human Services (DHHS) under contract no. HHSN272201800013C. NIH grant R01AI132317 (D.N. and L.P.), HHSN contract 75N93019C00073 (J.K.W., E.D., and B.J.D.), and the Bill and Melinda Gates Foundation grant INV-004923 (I.A.W.). This research used resources of the SSRL, SLAC National Accelerator Laboratory, supported by the U.S. Department of Energy, Office of Science, Office of Basic Energy Sciences under contract DE-AC02–76SF00515. The SSRL Structural Molecular Biology Program is supported by the DOE Office of Biological and Environmental Research and the National Institutes of Health—National Institute of General Medical Sciences (including P41GM103393) and the Advanced Photon Source, a U.S. Department of Energy (DOE), Office of Science User Facility, operated for the DOE Office of Science by Argonne National Laboratory under contract DE-AC02-06CH11357. Extraordinary facility operations were supported in part by the DOE Office of Science through the National Virtual Biotechnology Laboratory, a consortium of DOE national laboratories focused on the response to COVID-19, with funding provided by the Coronavirus CARES Act.

AUTHOR CONTRIBUTIONS

Conceptualization, J.T. and C.D.; methodology, C.D., L. Peng, T.-H.L., C.T., C.-C.D.L., Y.C., L.W., I.K., Z.H., R.S.W., A.P., E.D., D.E.G., I.D., and S.M.; formal analysis, C.D., L. Peng, T.-H.L., C.T., C.-C.D.L., Y.C., L.W., L. Purser, A.J.R.C., C.-W.P., J.K.W., M.Y., M.M., J.S., and E.D.; data curation, M.P.; investigation, C.D., L. Peng, T.-H.L., C.T., C.-C.D.L., L.W., L. Purser, C.-W.P., J.K.W., M.Z., M.P., D.M., A.J.R.C., S.D., E.K., L.H., D.P., R.B., S.L., M.M., Y.Z., E.S.Y., M.C., and K.L.; resources, R.S.W. and S.M.; writing—original draft, C.D., T.-H.L., C.T., C.-C.D.L., I.A.W., and J.T.; writing—review & editing, all authors; visualization, C.D., L. Peng, T.-H.L., C.T., C.-C.D.L., Y.C., L.W., J.K.W., E.D., and J.T.; supervision, D.E.G., E.D., S.M., J.W.Y., C.S., P.D.C., J.R.M., M.R.H., D.N., I.A.W., and J.T.; funding acquisition, B.J.D., C.S., P.D.C., J.R.M., M.R.H., D.N., I.A.W., and J.T.

DECLARATION OF INTERESTS

J.T. and C.D. are co-inventors on a provisional patent (US Patent application no. 63/308,898) filed on the mAbs described in this study. J.K.W., E.D., and B.J.D. are employees of Integral Molecular; B.J.D. is a shareholder in this company. Y.C., S.D., E.K., L.H., D.L.P., R.B., S.L., and M.R.H. performed this work as employees of Laulima Government Solutions, LLC. M.M. performed this work as an employee of Tunnell Government Services, Inc., a subcontractor to Laulima Government Solutions, LLC. The content of this publication does not necessarily reflect the views or policies of the DHHS or of the institutions and companies with which the authors are affiliated. All other authors declare no competing interests.

Received: July 6, 2022
Revised: September 4, 2022
Accepted: October 13, 2022
Published: November 7, 2022

REFERENCES

Adams, P.D., Afonine, P.V., Bunkóczi, G., Chen, V.B., Davis, I.W., Echols, N., Headd, J.J., Hung, L.W., Kapral, G.J., Grosse-Kunstleve, R.W., et al. (2010). PHENIX: a comprehensive Python-based system for macromolecular structure solution. Acta Crystallogr. D Biol. Crystallogr. *66*, 213–221. https://doi.org/10.1107/S0907444909052925.

Bangaru, S., Antanasijevic, A., Kose, N., Sewall, L.M., Jackson, A.M., Suryadevara, N., Zhan, X., Torres, J.L., Copps, J., de la Peña, A.T., et al. (2022). Structural mapping of antibody landscapes to human betacoronavirus spike proteins. Sci. Adv. *8*, eabn2911. https://doi.org/10.1126/sciadv.abn2911.

CellPress
OPEN ACCESS

Barnes, C.O., Jette, C.A., Abernathy, M.E., Dam, K.A., Esswein, S.R., Gristick, H.B., Malyutin, A.G., Sharaf, N.G., Huey-Tubman, K.E., Lee, Y.E., et al. (2020). SARS-CoV-2 neutralizing antibody structures inform therapeutic strategies. Nature *588*, 682–687. https://doi.org/10.1038/s41586-020-2852-1.

Barouch, D.H., Yang, Z.Y., Kong, W.P., Korioth-Schmitz, B., Sumida, S.M., Truitt, D.M., Kishko, M.G., Arthur, J.C., Miura, A., Mascola, J.R., et al. (2005). A human T-cell leukemia virus type 1 regulatory element enhances the immunogenicity of human immunodeficiency virus type 1 DNA vaccines in mice and nonhuman primates. J. Virol. *79*, 8828–8834. https://doi.org/10.1128/JVI.79.14.8828-8834.2005.

Bartsch, Y.C., Wang, C., Zohar, T., Fischinger, S., Atyeo, C., Burke, J.S., Kang, J., Edlow, A.G., Fasano, A., Baden, L.R., et al. (2021). Humoral signatures of protective and pathological SARS-CoV-2 infection in children. Nat. Med. *27*, 454–462. https://doi.org/10.1038/s41591-021-01263-3.

Briney, B., Inderbitzin, A., Joyce, C., and Burton, D.R. (2019). Commonality despite exceptional diversity in the baseline human antibody repertoire. Nature *566*, 393–397. https://doi.org/10.1038/s41586-019-0879-y.

Chen, V.B., Arendall, W.B., 3rd, Headd, J.J., Keedy, D.A., Immormino, R.M., Kapral, G.J., Murray, L.W., Richardson, J.S., and Richardson, D.C. (2010). MolProbity: all-atom structure validation for macromolecular crystallography. Acta Crystallogr. D Biol. Crystallogr. *66*, 12–21. https://doi.org/10.1107/S0907444909042073.

Chen, F., Tzarum, N., Wilson, I.A., and Law, M. (2019). VH1-69 antiviral broadly neutralizing antibodies: genetics, structures, and relevance to rational vaccine design. Curr. Opin. Virol. *34*, 149–159. https://doi.org/10.1016/j.coviro.2019.02.004.

Chen, E.C., Gilchuk, P., Zost, S.J., Suryadevara, N., Winkler, E.S., Cabel, C.R., Binshtein, E., Chen, R.E., Sutton, R.E., Rodriguez, J., et al. (2021). Convergent antibody responses to the SARS-CoV-2 spike protein in convalescent and vaccinated individuals. Cell Rep. *36*, 109604. https://doi.org/10.1016/j.celrep.2021.109604.

Cho, H., Gonzales-Wartz, K.K., Huang, D., Yuan, M., Peterson, M., Liang, J., Beutler, N., Torres, J.L., Cong, Y., Postnikova, E., et al. (2021). Bispecific antibodies targeting distinct regions of the spike protein potently neutralize SARS-CoV-2 variants of concern. Sci. Transl. Med. *13*, eabj5413. https://doi.org/10.1126/scitranslmed.abj5413.

Christley, S., Aguiar, A., Blanck, G., Breden, F., Bukhari, S.A.C., Busse, C.E., Jaglale, J., Harikrishnan, S.L., Laserson, U., Peters, B., et al. (2020). The ADC API: a web API for the programmatic query of the AIRR data commons. Front. Big Data *3*, 22. https://doi.org/10.3389/fdata.2020.00022.

Connolly, M.L. (1983). Analytical molecular surface calculation. J. Appl. Crystallogr. *16*, 548–558. https://doi.org/10.1107/S0021889883010985.

Corrie, B.D., Marthandan, N., Zimonja, B., Jaglale, J., Zhou, Y., Barr, E., Knoetze, N., Breden, F.M.W., Christley, S., Scott, J.K., et al. (2018). iReceptor: A platform for querying and analyzing antibody/B-cell and T-cell receptor repertoire data across federated repositories. Immunol. Rev. *284*, 24–41. https://doi.org/10.1111/imr.12666.

Covés-Datson, E.M., Dyall, J., DeWald, L.E., King, S.R., Dube, D., Legendre, M., Nelson, E., Drews, K.C., Gross, R., Gerhardt, D.M., et al. (2019). Inhibition of Ebola virus by a molecularly engineered banana lectin. PLoS Negl. Trop. Dis. *13*, e0007595. https://doi.org/10.1371/journal.pntd.0007595.

Crooks, G.E., Hon, G., Chandonia, J.M., and Brenner, S.E. (2004). WebLogo: a sequence logo generator. Genome Res. *14*, 1188–1190. https://doi.org/10.1101/gr.849004.

Crosnier, C., Wanaguru, M., McDade, B., Osier, F.H., Marsh, K., Rayner, J.C., and Wright, G.J. (2013). A library of functional recombinant cell-surface and secreted *P. falciparum* merozoite proteins. Mol. Cell. Proteomics *12*, 3976–3986. https://doi.org/10.1074/mcp.O113.028357.

Dacon, C., Tucker, C., Peng, L., Lee, C.-C.D., Lin, T.-H., Yuan, M., Cong, Y., Wang, L., Purser, L., Williams, J.K., et al. (2022). Broadly neutralizing antibodies target the coronavirus fusion peptide. Science *377*, 728–735. https://doi.org/10.1126/science.abq3773.

Davidson, E., and Doranz, B.J. (2014). A high-throughput shotgun mutagenesis approach to mapping B-cell antibody epitopes. Immunology *143*, 13–20. https://doi.org/10.1111/imm.12323.

DeKosky, B.J., Kojima, T., Rodin, A., Charab, W., Ippolito, G.C., Ellington, A.D., and Georgiou, G. (2015). In-depth determination and analysis of the human paired heavy- and light-chain antibody repertoire. Nat. Med. *21*, 86–91. https://doi.org/10.1038/nm.3743.

DeKosky, B.J., Lungu, O.I., Park, D., Johnson, E.L., Charab, W., Chrysostomou, C., Kuroda, D., Ellington, A.D., Ippolito, G.C., Gray, J.J., and Georgiou, G. (2016). Large-scale sequence and structural comparisons of human naive and antigen-experienced antibody repertoires. Proc. Natl. Acad. Sci. USA *113*, E2636–E2645. https://doi.org/10.1073/pnas.1525510113.

DeWitt, W.S., Lindau, P., Snyder, T.M., Sherwood, A.M., Vignali, M., Carlson, C.S., Greenberg, P.D., Duerkopp, N., Emerson, R.O., and Robins, H.S. (2016). A public database of memory and naive B-cell receptor sequences. PLoS One *11*, e0160853. https://doi.org/10.1371/journal.pone.0160853.

Dong, E., Du, H., and Gardner, L. (2020). An interactive web-based dashboard to track COVID-19 in real time. Lancet Infect. Dis. *20*, 533–534. https://doi.org/10.1016/S1473-3099(20)30120-1.

Dougan, M., Nirula, A., Azizad, M., Mocherla, B., Gottlieb, R.L., Chen, P., Hebert, C., Perry, R., Boscia, J., Heller, B., et al. (2021). Bamlanivimab plus etesevimab in mild or moderate Covid-19. N. Engl. J. Med. *385*, 1382–1392. https://doi.org/10.1056/NEJMoa2102685.

Dreyfus, C., Laursen, N.S., Kwaks, T., Zuijdgeest, D., Khayat, R., Ekiert, D.C., Lee, J.H., Metlagel, Z., Bujny, M.V., Jongeneelen, M., et al. (2012). Highly conserved protective epitopes on influenza B viruses. Science *337*, 1343–1348. https://doi.org/10.1126/science.1222908.

Edwards, A.M., Baric, R.S., Saphire, E.O., and Ulmer, J.B. (2022). Stopping pandemics before they start: lessons learned from SARS-CoV-2. Science *375*, 1133–1139. https://doi.org/10.1126/science.abn1900.

Ekiert, D.C., Bhabha, G., Elsliger, M.A., Friesen, R.H.E., Jongeneelen, M., Throsby, M., Goudsmit, J., and Wilson, I.A. (2009). Antibody recognition of a highly conserved influenza virus epitope. Science *324*, 246–251. https://doi.org/10.1126/science.1171491.

Ekiert, D.C., Kashyap, A.K., Steel, J., Rubrum, A., Bhabha, G., Khayat, R., Lee, J.H., Dillon, M.A., O'Neil, R.E., Faynboym, A.M., et al. (2012). Cross-neutralization of influenza A viruses mediated by a single antibody loop. Nature *489*, 526–532. https://doi.org/10.1038/nature11414.

Elbe, S., and Buckland-Merrett, G. (2017). Data, disease and diplomacy: GISAID's innovative contribution to global health. Glob. Chall. *1*, 33–46. https://doi.org/10.1002/gch2.1018.

Emsley, P., Lohkamp, B., Scott, W.G., and Cowtan, K. (2010). Features and development of coot. Acta Crystallogr. D Biol. Crystallogr. *66*, 486–501. https://doi.org/10.1107/S0907444910007493.

Fung, T.S., and Liu, D.X. (2019). Human coronavirus: host-pathogen interaction. Annu. Rev. Microbiol. *73*, 529–557. https://doi.org/10.1146/annurev-micro-020518-115759.

Gu, Z., Gu, L., Eils, R., Schlesner, M., and Brors, B. (2014). Circlize implements and enhances circular visualization in R. Bioinformatics *30*, 2811–2812. https://doi.org/10.1093/bioinformatics/btu393.

Gupta, A., Gonzalez-Rojas, Y., Juarez, E., Crespo Casal, M., Moya, J., Falci, D.R., Sarkis, E., Solis, J., Zheng, H., Scott, N., et al. (2021). Early treatment for Covid-19 with SARS-CoV-2 neutralizing antibody sotrovimab. N. Engl. J. Med. *385*, 1941–1950. https://doi.org/10.1056/NEJMoa2107934.

Huang, J., Doria-Rose, N.A., Longo, N.S., Laub, L., Lin, C.L., Turk, E., Kang, B.H., Migueles, S.A., Bailer, R.T., Mascola, J.R., and Connors, M. (2013). Isolation of human monoclonal antibodies from peripheral blood B cells. Nat. Protoc. *8*, 1907–1915. https://doi.org/10.1038/nprot.2013.117.

Jackson, K.J., Wang, Y., Gaeta, B.A., Pomat, W., Siba, P., Rimmer, J., Sewell, W.A., and Collins, A.M. (2012). Divergent human populations show extensive shared IGK rearrangements in peripheral blood B cells. Immunogenetics *64*, 3–14. https://doi.org/10.1007/s00251-011-0559-z.

Kepler, T.B. (2013). Reconstructing a B-cell clonal lineage. I. Statistical inference of unobserved ancestors. F1000Res *2*, 103. https://doi.org/10.12688/f1000research.2-103.v1.

Krissinel, E., and Henrick, K. (2007). Inference of macromolecular assemblies from crystalline state. J. Mol. Biol. *372*, 774–797. https://doi.org/10.1016/j.jmb.2007.05.022.

Lefranc, M.P. (2014). Immunoglobulin and T cell receptor genes: IMGT® and the birth and rise of Immunoinformatics. Front. Immunol. *5*, 22. https://doi.org/10.3389/fimmu.2014.00022.

Letunic, I., and Bork, P. (2021). Interactive Tree Of Life (iTOL) v5: an online tool for phylogenetic tree display and annotation. Nucleic Acids Res. *49*, W293–W296. https://doi.org/10.1093/nar/gkab301.

Li, F. (2015). Receptor recognition mechanisms of coronaviruses: a decade of structural studies. J. Virol. *89*, 1954–1964. https://doi.org/10.1128/JVI.02615-14.

Li, F. (2016). Structure, function, and evolution of coronavirus spike proteins. Annu. Rev. Virol. *3*, 237–261. https://doi.org/10.1146/annurev-virology-110615-042301.

Li, W., Chen, Y., Prévost, J., Ullah, I., Lu, M., Gong, S.Y., Tauzin, A., Gasser, R., Vézina, D., Anand, S.P., et al. (2022). Structural basis and mode of action for two broadly neutralizing antibodies against SARS-CoV-2 emerging variants of concern. Cell Rep. *38*, 110210. https://doi.org/10.1016/j.celrep.2021.110210.

Lv, H., Tsang, O.T., So, R.T.Y., Wang, Y., Yuan, M., Liu, H., Yip, G.K., Teo, Q.W., Lin, Y., Liang, W., et al. (2021). Homologous and heterologous serological response to the N-terminal domain of SARS-CoV-2 in humans and mice. Eur. J. Immunol. *51*, 2296–2305. https://doi.org/10.1002/eji.202149234.

McCoy, A.J., Grosse-Kunstleve, R.W., Adams, P.D., Winn, M.D., Storoni, L.C., and Read, R.J. (2007). Phaser crystallographic software. J. Appl. Crystallogr. *40*, 658–674. https://doi.org/10.1107/S0021889807021206.

Moir, S., Lapointe, R., Malaspina, A., Ostrowski, M., Cole, C.E., Chun, T.W., Adelsberger, J., Baseler, M., Hwu, P., and Fauci, A.S. (1999). CD40-mediated induction of CD4 and CXCR4 on B lymphocytes correlates with restricted susceptibility to human immunodeficiency virus type 1 infection: potential role of B lymphocytes as a viral reservoir. J. Virol. *73*, 7972–7980. https://doi.org/10.1128/JVI.73.10.7972-7980.1999.

Murugan, R., Buchauer, L., Triller, G., Kreschel, C., Costa, G., Pidelaserra Martí, G., Imkeller, K., Busse, C.E., Chakravarty, S., Sim, B.K.L., et al. (2018). Clonal selection drives protective memory B cell responses in controlled human malaria infection. Sci. Immunol. *3*, eaap8029. https://doi.org/10.1126/sciimmunol.aap8029.

Naldini, L., Blömer, U., Gage, F.H., Trono, D., and Verma, I.M. (1996). Efficient transfer, integration, and sustained long-term expression of the transgene in adult rat brains injected with a lentiviral vector. Proc. Natl. Acad. Sci. USA *93*, 11382–11388. https://doi.org/10.1073/pnas.93.21.11382.

Otwinowski, Z., and Minor, W. (1997). Processing of X-ray diffraction data collected in oscillation mode. Methods Enzymol. *276*, 307–326. https://doi.org/10.1016/S0076-6879(97)76066-X.

Peck, K.M., Burch, C.L., Heise, M.T., and Baric, R.S. (2015). Coronavirus host range expansion and Middle East Respiratory Syndrome coronavirus emergence: biochemical mechanisms and evolutionary perspectives. Annu. Rev. Virol. *2*, 95–117. https://doi.org/10.1146/annurev-virology-100114-055029.

Pinto, D., Sauer, M.M., Czudnochowski, N., Low, J.S., Tortorici, M.A., Housley, M.P., Noack, J., Walls, A.C., Bowen, J.E., Guarino, B., et al. (2021). Broad betacoronavirus neutralization by a stem helix-specific human antibody. Science *373*, 1109–1116. https://doi.org/10.1126/science.abj3321.

Rogers, T.F., Zhao, F., Huang, D., Beutler, N., Burns, A., He, W.T., Limbo, O., Smith, C., Song, G., Woehl, J., et al. (2020). Isolation of potent SARS-CoV-2 neutralizing antibodies and protection from disease in a small animal model. Science *369*, 956–963. https://doi.org/10.1126/science.abc7520.

Sauer, M.M., Tortorici, M.A., Park, Y.J., Walls, A.C., Homad, L., Acton, O.J., Bowen, J.E., Wang, C., Xiong, X., de van der Schueren, W., et al. (2021). Structural basis for broad coronavirus neutralization. Nat. Struct. Mol. Biol. *28*, 478–486. https://doi.org/10.1038/s41594-021-00596-4.

Scheid, J.F., Mouquet, H., Ueberheide, B., Diskin, R., Klein, F., Oliveira, T.Y., Pietzsch, J., Fenyo, D., Abadir, A., Velinzon, K., et al. (2011). Sequence and structural convergence of broad and potent HIV antibodies that mimic CD4 binding. Science *333*, 1633–1637. https://doi.org/10.1126/science.1207227.

Schindelin, J., Arganda-Carreras, I., Frise, E., Kaynig, V., Longair, M., Pietzsch, T., Preibisch, S., Rueden, C., Saalfeld, S., Schmid, B., et al. (2012). Fiji: an open-source platform for biological-image analysis. Nat. Methods *9*, 676–682. https://doi.org/10.1038/nmeth.2019.

Schneider, T.D., and Stephens, R.M. (1990). Sequence logos: a new way to display consensus sequences. Nucl. Acids Res. *18*, 6097–6100. https://doi.org/10.1093/nar/18.20.6097.

Shah, P., Canziani, G.A., Carter, E.P., and Chaiken, I. (2021). The case for S2: the potential benefits of the S2 subunit of the SARS-CoV-2 spike protein as an immunogen in fighting the COVID-19 pandemic. Front. Immunol. *12*, 637651. https://doi.org/10.3389/fimmu.2021.637651.

Starr, T.N., Greaney, A.J., Hilton, S.K., Ellis, D., Crawford, K.H.D., Dingens, A.S., Navarro, M.J., Bowen, J.E., Tortorici, M.A., Walls, A.C., et al. (2020). Deep mutational scanning of SARS-CoV-2 receptor binding domain reveals constraints on folding and ACE2 binding. Cell *182*, 1295–1310.e20. https://doi.org/10.1016/j.cell.2020.08.012.

Takashita, E., Kinoshita, N., Yamayoshi, S., Sakai-Tagawa, Y., Fujisaki, S., Ito, M., Iwatsuki-Horimoto, K., Chiba, S., Halfmann, P., Nagai, H., et al. (2022a). Efficacy of antibodies and antiviral drugs against Covid-19 Omicron variant. N. Engl. J. Med. *386*, 995–998. https://doi.org/10.1056/NEJMc2119407.

Takashita, E., Yamayoshi, S., Simon, V., van Bakel, H., Sordillo, E.M., Pekosz, A., Fukushi, S., Suzuki, T., Maeda, K., Halfmann, P., et al. (2022b). Efficacy of antibodies and antiviral drugs against Omicron BA.2.12.1, BA.4, and BA.5 subvariants. N. Engl. J. Med. *387*, 468–470. https://doi.org/10.1056/NEJMc2207519.

Tan, J., Sack, B.K., Oyen, D., Zenklusen, I., Piccoli, L., Barbieri, S., Foglierini, M., Fregni, C.S., Marcandalli, J., Jongo, S., et al. (2018). A public antibody lineage that potently inhibits malaria infection through dual binding to the circumsporozoite protein. Nat. Med. *24*, 401–407. https://doi.org/10.1038/nm.4513.

Tiller, T., Meffre, E., Yurasov, S., Tsuiji, M., Nussenzweig, M.C., and Wardemann, H. (2008). Efficient generation of monoclonal antibodies from single human B cells by single cell RT-PCR and expression vector cloning. J. Immunol. Methods *329*, 112–124. https://doi.org/10.1016/j.jim.2007.09.017.

Tipton, C.M., Fucile, C.F., Darce, J., Chida, A., Ichikawa, T., Gregoretti, I., Schieferl, S., Hom, J., Jenks, S., Feldman, R.J., et al. (2015). Diversity, cellular origin and autoreactivity of antibody-secreting cell population expansions in acute systemic lupus erythematosus. Nat. Immunol. *16*, 755–765. https://doi.org/10.1038/ni.3175.

V'Kovski, P., Kratzel, A., Steiner, S., Stalder, H., and Thiel, V. (2021). Coronavirus biology and replication: implications for SARS-CoV-2. Nat. Rev. Microbiol. *19*, 155–170. https://doi.org/10.1038/s41579-020-00468-6.

Wang, L., Shi, W., Joyce, M.G., Modjarrad, K., Zhang, Y., Leung, K., Lees, C.R., Zhou, T., Yassine, H.M., Kanekiyo, M., et al. (2015). Evaluation of candidate vaccine approaches for MERS-CoV. Nat. Commun. *6*, 7712. https://doi.org/10.1038/ncomms8712.

Wang, L.T., Pereira, L.S., Flores-Garcia, Y., O'Connor, J., Flynn, B.J., Schön, A., Hurlburt, N.K., Dillon, M., Yang, A.S.P., Fabra-García, A., et al. (2020). A potent anti-malarial human monoclonal antibody targets circumsporozoite protein minor repeats and neutralizes sporozoites in the liver. Immunity *53*, 733–744.e8. https://doi.org/10.1016/j.immuni.2020.08.014.

Wang, C., van Haperen, R., Gutiérrez-Álvarez, J., Li, W., Okba, N.M.A., Albulescu, I., Widjaja, I., van Dieren, B., Fernandez-Delgado, R., Sola, I., et al. (2021). A conserved immunogenic and vulnerable site on the coronavirus spike protein delineated by cross-reactive monoclonal antibodies. Nat. Commun. *12*, 1715. https://doi.org/10.1038/s41467-021-21968-w.

Wei, C.J., Boyington, J.C., Dai, K., Houser, K.V., Pearce, M.B., Kong, W.P., Yang, Z.Y., Tumpey, T.M., and Nabel, G.J. (2010). Cross-neutralization of 1918 and 2009 influenza viruses: role of glycans in viral evolution and vaccine design. Sci. Transl. Med. *2*, 24ra21. https://doi.org/10.1126/scitranslmed.3000799.

Weinreich, D.M., Sivapalasingam, S., Norton, T., Ali, S., Gao, H., Bhore, R., Xiao, J., Hooper, A.T., Hamilton, J.D., Musser, B.J., et al. (2021).

CellPress
OPEN ACCESS

REGEN-COV antibody combination and outcomes in outpatients with Covid-19. N. Engl. J. Med. *385*, e81. https://doi.org/10.1056/NEJMoa2108163.

Wu, X., Zhou, T., Zhu, J., Zhang, B., Georgiev, I., Wang, C., Chen, X., Longo, N.S., Louder, M., McKee, K., et al. (2011). Focused evolution of HIV-1 neutralizing antibodies revealed by structures and deep sequencing. Science *333*, 1593–1602. https://doi.org/10.1126/science.1207532.

Yamasoba, D., Kosugi, Y., Kimura, I., Fujita, S., Uriu, K., Ito, J., and Sato, K.; Genotype to Phenotype Japan (G2P-Japan) Consortium (2022). Neutralisation sensitivity of SARS-CoV-2 omicron subvariants to therapeutic monoclonal antibodies. Lancet Infect. Dis. *22*, 942–943. https://doi.org/10.1016/S1473-3099(22)00365-6.

Yuan, M., Liu, H., Wu, N.C., Lee, C.D., Zhu, X., Zhao, F., Huang, D., Yu, W., Hua, Y., Tien, H., et al. (2020a). Structural basis of a shared antibody response to SARS-CoV-2. Science *369*, 1119–1123. https://doi.org/10.1126/science.abd2321.

Yuan, M., Wu, N.C., Zhu, X., Lee, C.D., So, R.T.Y., Lv, H., Mok, C.K.P., and Wilson, I.A. (2020b). A highly conserved cryptic epitope in the receptor binding domains of SARS-CoV-2 and SARS-CoV. Science *368*, 630–633. https://doi.org/10.1126/science.abb7269.

Zhou, P., Song, G., He, W.T., Beutler, N., Tse, L.V., Martinez, D.R., Schäfer, A., Anzanello, F., Yong, P., Peng, L., et al. (2022a). Broadly neutralizing anti-S2 antibodies protect against all three human betacoronaviruses that cause severe disease. bioRxiv. 2022.2003.2004.479488. https://doi.org/10.1101/2022.03.04.479488.

Zhou, P., Yuan, M., Song, G., Beutler, N., Shaabani, N., Huang, D., He, W.T., Zhu, X., Callaghan, S., Yong, P., et al. (2022b). A human antibody reveals a conserved site on beta-coronavirus spike proteins and confers protection against SARS-CoV-2 infection. Sci. Transl. Med. *14*, eabi9215. https://doi.org/10.1126/scitranslmed.abi9215.

Zhou, T., Wang, L., Misasi, J., Pegu, A., Zhang, Y., Harris, D.R., Olia, A.S., Talana, C.A., Yang, E.S., Chen, M., et al. (2022c). Structural basis for potent antibody neutralization of SARS-CoV-2 variants including B.1.1.529. Science *376*, eabn8897. https://doi.org/10.1126/science.abn8897.

STAR★METHODS

KEY RESOURCES TABLE

REAGENT OR RESOURCE	SOURCE	IDENTIFIER
**Antibodies**		
CD14-BV510	Biolegend	Cat. # 301842; RRID: AB_2561946
CD3-BV510	Biolegend	Cat. # 317332; RRID: AB_2561943
CD56-BV510	Biolegend	Cat. # 318340; RRID: AB_2561944
CD19-ECD	Beckman Coulter	Cat. # IM2708U; RRID: AB_130854
CD21-BV711	BD	Cat. # 563163; RRID: AB_2738040
IgA-Alexa Fluor 647	Jackson Immunoresearch	Cat. # 109-606-011; RRID: AB_2337895
IgD-PE-Cy7	BD	Cat. # 561314; RRID: AB_10642457
IgM-PerCP-Cy5.5	BD	Cat. # 561285; RRID: AB_10611998
CD27-Alexa Fluor 488	Biolegend	Cat. # 393204; RRID: AB_2750089
CD38-APC-Cy7	Biolegend	Cat. # 303534; RRID: AB_2561605
goat anti-human IgG-Alexa Fluor 647	Jackson Immunoresearch	Cat. # 109-606-170; RRID: AB_2337902
goat anti-human IgA-Cy3	Jackson Immunoresearch	Cat. # 109-166-011; RRID: AB_2337733
Alexa-Fluor-488-conjugated anti-IgG	Jackson Immunoresearch	Cat. # 109-545-003; RRID: AB_2337831
CC6.29	Rogers et al. (2020)	N/A
CC6.33	Rogers et al. (2020)	N/A
L25-dP06E11	Rogers et al. (2020)	N/A
CC12.23	Rogers et al. (2020)	N/A
CC12.25	Rogers et al. (2020)	N/A
anti-MERS-CoV spike primary antibody	Sino Biological	Cat. # 40069-R723; RRID: AB_2860455
goat anti-rabbit antibody Alexa Fluor 647	Life Technologies	Cat. # A21245; RRID: AB_2535813
Anti-His biotin	Invitrogen	Cat. # MA1-21315-BTIN; RRID: AB_2536983
L9	Wang et al. (2020)	N/A
**Virus strains**		
SARS-CoV-2	Centers for Disease Control and Prevention (CDC)	GenBank: MT952134
MERS-CoV	Armed Forces Health Surveillance Center	GenBank: KC776174
GFP-expressing HCoV-OC43 virus	Yewdell lab, NIAID	N/A
**Biological samples**		
COVID-19 convalescent human blood and plasma samples	New York Blood Center	N/A
Plasma/serum samples from mRNA-1273 vaccine (Moderna) recipients	NIH Clinical Research Center	N/A
**Chemicals, peptides and recombinant proteins**		
HCoV-NL63 spike	Sino Biological	Cat. # 40604-V08B-B
HCoV-229E spike	Sino Biological	Cat. # 40605-V08B-B
HCoV-HKU1 spike	Sino Biological	Cat. # 40606-V08B
HCoV-OC43 spike	Bangaru et al. (2022)	N/A
MERS-CoV spike	Bangaru et al. (2022)	N/A
NL63-CoV spike	Sino Biological	Cat. # 40604-V08B-B
229E-CoV spike	Sino Biological	Cat. # 40605-V08B-B
HKU1-CoV spike	Sino Biological	Cat. # 40606-V08B
SARS-CoV-2 S2 subunit	R&D Systems	Cat. # 10594-CV-100
Protein G Elution Buffer	Thermo Scientific	Cat. # 21004
IL21	Gibco	Cat. # PHC0211

(*Continued on next page*)

CellPress
OPEN ACCESS

Continued

REAGENT OR RESOURCE	SOURCE	IDENTIFIER
R848	Invivogen	Cat. # tlrl-r848
Mycozap	Lonza	Cat. # VZA-2021
Gentamicin	Quality Biological	Cat. # 120-098-661
GlutaMax	Gibco	Cat. # 11965-092
Normocin	Invivogen	Cat. # ant-nr-1
Puromycin	Invivogen	Cat. # ant-pr-1
Hygromycin B Gold	Invivogen	Cat. # ant-hg-1
Zeocin	Invivogen	Cat. # ant-zn-05
Penicillin/Streptomycin	Invitrogen	Cat. # 15140122
A/Solomon Islands/03/2006 (H1N1) hemagglutinin ectodomain	Ekiert et al. (2012)	N/A
CD4	Crosnier et al. (2013)	N/A
2.5 μm streptavidin beads, Yellow, Odd # peaks	Spherotech	Cat. # SVFA-2552-6K
2.5 μm streptavidin beads, Yellow, Even # peaks	Spherotech	Cat. # SVFB-2552-6K
2.5 μm streptavidin beads, Pink, Odd # peaks	Spherotech	Cat. # SVFA-2558-6K
2.5 μm streptavidin beads, Pink, Even # peaks	Spherotech	Cat. # SVFB-2558-6K
7 μm streptavidin beads	Spherotech	Cat. # SVP-60-5
Lipofectamine 2000	ThermoFisher Scientific	Cat. # 11668019
Lipofectamine 3000 transfection reagent	ThermoFisher Scientific	Cat. # L3000-001
Lyophilized 15-mer peptides	JPT Peptide Technologies	N/A
Horseradish peroxidase	Invitrogen	Cat. # A18817
POD substrate	Roche	Cat. # 11582950001
Dynabeads mRNA DIRECT lysis buffer	Life Technologies	Cat. # 61011
HyClone insect cell culture medium	GE Healthcare	Cat. # SH30280.03
PEIMax	Polysciences	Cat. # 24765-1
QUANTI-Blue Solution	Invivogen	Cat. # rep-qbs
**Critical commercial assays**		
Bac-to-Bac system	Life Technologies	Cat. # 10359016
Bright-Glo Luciferase Assay System	Promega	Cat. # E2620
ScisGo®-HLA-v6 kit	Scisco Genetics Inc.	Cat. # HLA-24S-v6
Pierce Fab Preparation kit	ThermoFisher Scientific	Cat. # 44985
ExpiCHO expression system	ThermoFisher Scientific	Cat. # A29133
Expi293F expression system	ThermoFisher Scientific	Cat. # A14635
ExpiFectamine 293 Transfection Kit	ThermoFisher Scientific	Cat. # A14524
**Deposited data**		
Mouse Hepatitis Virus	UniProt	UniProt: P11224
HCoV-OC43	UniProt	UniProt: P36334
HCoV-HKU1 N5	UniProt	UniProt: Q0ZME7
BatCoV-HKU3	UniProt	UniProt: Q3LZX1
BatCoV-RaTG13	GenBank	GenBank: QHR63300
BatCoV-Rs4231	GenBank	GenBank: ATO98157
BatCoV-Rs3367	GenBank	GenBank: AGZ48818
BatCoV-WIV1	GenBank	GenBank: AGZ48831
Civet-SARS-CoV-007/004	GenBank	GenBank: AAU04646
Pangolin-CoV-GX-P2V	GenBank	GenBank: QIQ54048
SARS-CoV-1-Tor2	GenBank	GenBank: AAP41037
SARS-CoV-1-Urbani	GenBank	GenBank: AAP13441
SARS-CoV-2 Wuhan-Hu-1	GenBank	GenBank: YP_009724390
B.1.1.7	GenBank	GenBank: QWE88920
B.1.351	GenBank	GenBank: QRN78347

(*Continued on next page*)

Continued

REAGENT OR RESOURCE	SOURCE	IDENTIFIER
P.1	GenBank	GenBank: QVE55289
B.1.617.2	GenBank	GenBank: QWK65230
BA.1	GenBank	GenBank: UFO69279
BA.2	GenBank	GenBank: UJE45220
BA.2.75	GenBank	GenBank: UTM82166.1
BA.5	GenBank	GenBank: UOZ45804.1
Bat-CoV-HKU4	UniProt	UniProt: A3EX94
BatCoV-HKU5	GenBank	GenBank: YP_001039962.1
MERS-EMC/2012	GenBank	GenBank: YP_009047204
BatCoV-GCCDC1	GenBank	GenBank: YP_009273005.1
BtRt-BetaCoV/GX2018	GenBank	GenBank: QJX58383.1
BatCoV-HKU9	GenBank	GenBank: ABN10911
Bat Hp-betacoronavirus/Zhejiang2013	GenBank	GenBank: YP_009072440
SARS-CoV-2	GenBank	GenBank: QHD43416.1
SARS-CoV	GenBank	GenBank: AAP13441.1
MERS-CoV	GenBank	GenBank: AFS88936
HCoV-NL63	GenBank	GenBank: Q6Q1S2.1
MesAur1.0 genome assembly	GenBank	GenBank: GCA_00349664.1
Stem helix-specific mAbs	GenBank	GenBank: OP377774-OP377795
COV30-14 crystal structure	PDB	PDB: 8DTR
COV89-22 crystal structure	PDB	PDB: 8DTX
COV93-03 crystal structure	PDB	PDB: 8DTT
Experimental models: cell lines		
Sf9 cells	ATCC	Cat. # CRL-1711; RRID: CVCL_0549
High Five cells	ThermoFisher Scientific	Cat. # B85502; RRID: CVCL_C190
FreeStyle 293-F cells	ThermoFisher Scientific	Cat. # R79007; RRID: CVCL_D603
Irradiated 3T3-CD40L cells	Huang et al. (2013)	N/A
HeLa cells	ATCC	Cat. # CCL-2; RRID: CVCL_0030
Rhabdomyosarcoma cells	ATCC	Cat. # CCL136
VRC8400 cells	Barouch et al. (2005)	N/A
HEK-293T cells	ATCC	Cat. # CRL-11268; RRID: CVCL_1926
293 flpin-TMPRSS2-ACE2 cells	Dr. Adrian Creanga, VRC/NIH; Zhou et al. (2022c)	N/A
Huh7.5 cells	Dr. Deborah Taylor, US FDA; Wang et al. (2015)	N/A
Expi293 cells	Gibco	Cat. # A14527; RRID: CVCL_D615
Vero E6 cells	Expasy CVCL_XD71	Cat. # BEI NR-596; RRID: CVCL_XD71
HEK-Blue hACE2-TMPRSS2 cells	Invivogen	Cat. # hkb-hace2tpsa
293-hMyD88 cells	Invivogen	Cat. # 293-hmyd
Oligonucleotides		
PCR primers for amplification of antibody heavy, kappa, and lambda genes	Wang et al. (2020)	GenBank: MT811859 – MT811914
PCR primers for generation of virus F1148, L1152 and F1156 mutants	Table S3 (this study)	N/A
Experimental models: organisms		
Golden Syrian Hamsters	Envigo (Indianapolis, IN USA)	N/A
Recombinant DNA		
pCMV-dR82 dvpr ($Assay_{Scripps}$)	Addgene	Cat. # 8455; RRID: Addgene_8455
pBOBI-FLuc ($Assay_{Scripps}$)	Addgene	Cat. # 170674; RRID: Addgene_170674
SARS-CoV ($Assay_{Scripps}$)	Addgene	Cat. # 170447; RRID: Addgene_170447

(*Continued on next page*)

Continued

REAGENT OR RESOURCE	SOURCE	IDENTIFIER
SARS-CoV-2 ($Assay_{Scripps}$)	Addgene	Cat. # 170442; RRID: Addgene_170442
MERS-CoV ($Assay_{Scripps}$)	Addgene	Cat. # 170448; RRID: Addgene_170448
HCoV-NL63 ($Assay_{Scripps}$)	Addgene	Cat. # 172666; RRID: Addgene_172666
SARS-CoV-2 Alpha ($Assay_{Scripps}$)	Addgene	Cat. # 170451; RRID: Addgene_170451
SARS-CoV-2 Beta ($Assay_{Scripps}$)	Addgene	Cat. # 170449; RRID: Addgene_170449
SARS-CoV-2 Gamma ($Assay_{Scripps}$)	Addgene	Cat. # 170450; RRID: Addgene_170450
SARS-CoV-2 Delta ($Assay_{Scripps}$)	Addgene	Cat. # 172320; RRID: Addgene_172320
SARS-CoV-2 BA.1 ($Assay_{Scripps}$)	Addgene	Cat. # 180375; RRID: Addgene_180375
SARS-CoV-2 BA.2 ($Assay_{Scripps}$)	Addgene	Cat. # 183700; RRID: Addgene_183700
SARS-CoV-2 BA.2.12.1 ($Assay_{Scripps}$)	Addgene	Cat. # 186809
SARS-CoV-2 BA.4/5 ($Assay_{Scripps}$)	Addgene	Cat. # 186810
SARS-CoV-2 BA.2.75 ($Assay_{Scripps}$)	Addgene	Cat. # 190674
pFastBac-SARS-CoV-2-RBD	Yuan et al. (2020b)	N/A
pFastBac-SI06-HA	Ekiert et al. (2012)	N/A
pHR' CMV-Luc	Naldini et al. (1996)	N/A
TMPRSS2 plasmid	Wei et al. (2010)	N/A
**Software and algorithms**		
MAFFT v7 server	https://mafft.cbrc.jp/alignment/server/	N/A
Weblogo 3.0 server	https://weblogo.threeplusone.com/	N/A
Chimera X	https://www.rbvi.ucsf.edu/chimerax/	N/A
Geneious Prime	https://www.geneious.com	Version 2021.0.3
Cloanalyst	Kepler (2013)	N/A
Interactive Tree of Life	https://itol.embl.de/	N/A
Sciscloud	Scisco Genetics Inc.	N/A
FlowJo	BD	Version 10.8.1
GraphPad Prism	Graphpad	Version 9.3.1
iReceptor database	https://gateway.ireceptor.org/home	N/A
Epitope Software	Carterra	N/A
Kinetics Software	Carterra	N/A
International Immunogenetics Information System database (IMGT)	https://www.imgt.org/	N/A
HKL2000	Otwinowski and Minor (1997)	N/A
Phaser	McCoy et al. (2007)	N/A
Repertoire Builder	https://sysimm.org/rep_builder/	N/A
Coot	Emsley et al. (2010)	N/A
PHENIX	Adams et al. (2010)	N/A
PISA	Krissinel and Henrick (2007)	N/A
Molecular Surface package	Connolly (1983)	N/A
Fiji ImageJ	Schindelin et al. (2012)	N/A
GISAID	https://gisaid.org/publish/	N/A
**Other**		
iQue Screener Plus	Intellicyt	N/A
BD FACSAria IIIu	Becton Dickinson	N/A
Beacon analyzer	Berkeley Lights	N/A
MiSeq	Illumina, San Diego, CA	N/A
LSA	Carterra	N/A
CrystalMation system	Rigaku	N/A
BZ-X fluorescence microscope	KEYENCE	N/A
Microbeta	PerkinElmer	N/A
Operetta imaging system	PerkinElmer	N/A
Enspire multi-mode plate reader	PerkinElmer	N/A

RESOURCE AVAILABILITY

Lead contact

Further information and requests for resources and reagents should be directed to and will be fulfilled by the lead contact, Joshua Tan (tanj4@nih.gov).

Materials availability

Antibodies described in this manuscript are available through a Materials Transfer Agreement (MTA) with the National Institute of Allergy and Infectious Diseases. Plasmids generated in this study have been deposited to Addgene.

Data and code availability

- Crystal structures have been deposited into the Protein Data Bank (PDB: 8DTR, 8DTT, 8DTX for COV30-14, COV93-03 and COV89-22, respectively). Antibody sequences have been deposited in GenBank (accession numbers OP377774-OP377795).
- This paper does not report original code.
- Any additional information required to reanalyze the data reported in this paper is available from the lead contact upon request.

EXPERIMENTAL MODEL AND SUBJECT DETAILS

Study cohort

Anonymized samples of whole blood and plasma from COVID-19 convalescent patients were obtained from a previously described cohort (Cho et al., 2021). Inclusion criteria included an age of 18 years or above and RT-PCR confirmation of SARS-CoV-2 infection. All samples were collected at least 2 weeks after resolution of symptoms, and all donors signaled consent by signing the standard New York Blood Center (NYBC) blood donor consent form. Participants met inclusion criteria and assented to provide samples. Of these, samples from 19 were selected for inclusion in this study following analysis of plasma IgG reactivity.

Whole blood, plasma and serum samples were collected from recipients of the SARS-CoV-2 mRNA-1273 vaccine (Moderna) at the NIH Clinical Research Center in Bethesda, MD under protocols approved by the NIH Institutional Review Board, ClinicalTrials.gov identifiers: NCT00001281 and NCT05078905. Inclusion criteria for the vaccine study were age ($\geq$ 18 years), HIV status (negative), no known history of SARS-CoV-2 infection (verified by nucleocapsid antibody responses), and no previous doses of COVID-19 vaccines. 16 participants met inclusion criteria and provided written informed consent to have their blood products used for research purposes. Blood samples were collected serially at baseline (prior to receiving the initial vaccine dose), 30 d after administration of the second dose, pre-booster (third dose) baseline, and 30 d after administration of the booster. A further blood sample was also collected from 3 participants at 30 d after documented SARS-CoV-2 infection. Samples were not randomized or blinded, but were anonymized.

Cell culture

Memory B cells (MBCs) were derived from cryopreserved peripheral blood mononuclear cells (PBMCs) by flow sort and cultured in IMDM (Gibco, 31980-030) supplemented with 10% HI-FBS (Gibco, 10438-026), 100 ng/mL IL21 (Gibco, PHC0211), 0.5 μg/mL R848 (Invivogen, tlrl-r848) and 1× Mycozap (Lonza, VZA-2021). Sf9 and High Five cells were cultured in HyClone insect cell culture medium (GE Healthcare, SH30280.03). Sf9 cells were seeded and incubated at 28°C in T25 and T175 flasks for bacmid transfection and generation of baculoviruses, respectively. High Five cells were incubated at 28°C with shaking at 110 rpm for 72 h for protein expression. Irradiated 3T3-CD40L cells were generated as previously described (Moir et al., 1999; Huang et al., 2013) and cryopreserved for use in MBC cultures. FreeStyle 293-F cells were cultured in Freestyle 293 Expression media (ThermoFisher Scientific, 12338018). HeLa cells were cultured in DMEM (Lonza, 08028) supplemented with 10% FBS, 1× penicillin/streptomycin and Glutamax. Rhabdomyosarcoma cells were cultured in DMEM (Gibco, 11966) supplemented with 10% HIFBS, 4500 mg/mL glucose, 1 mM sodium pyruvate, 1 mM HEPES and 50 μg/mL gentamicin (Quality Biological, 120-098-661) and cultured in a T225cm^2 flask at 37°C and 5% CO_2. HuH7.5 cells (provided by Dr. Deborah R. Taylor, US FDA), used to propagate MERS-CoV pseudovirus for use in neutralization assays, were cultured in DMEM with 10% BSA, 2 mM glutamine and 1× penicillin/streptomycin (D10). 293 flpin-TMPRSS2-ACE2 cells (provided by Dr. Adrian Creanga, VRC/NIH) were cultured in D10 with 100 μg/mL hygromycin.

Viruses

SARS-CoV-2 WA-01 (Genbank: MT952134) was obtained from the Centers for Disease Control and Prevention (CDC). MERS-CoV (Jordan-n3/2012; Genbank: KC776174) was provided by the Armed Forces Health Surveillance Center, Division of Global Emerging Infections Surveillance and Response System. All experiments with live SARS-CoV-2 and MERS-CoV were performed in a BSL-3 facility following National Institutes of Health safety guidelines.

Hamster model

Golden Syrian hamsters were sourced from Envigo (Indianapolis, IN USA). Animals were acclimated at IRF facility for 10 d and weighed 2 d prior to study commencement. Group selection was made at 5-6 weeks, assigning groups based on weight. Equal

numbers of males and females were assigned to each of eight groups of n = 12 according to weight. Animal research was conducted under an IACUC approved protocols at the IRF in compliance with the Animal Welfare Act and other federal statutes and regulations relating to animals and experiments involving animals. The facilities where this research was conducted are fully accredited by the Association for Assessment and Accreditation of Laboratory Animal Care, International and adheres to principles stated in the Guide for the Care and Use of Laboratory Animals, National Research Council, 2011.

METHOD DETAILS

Coronavirus spike proteins

Expression of SARS-CoV-2 spike, SARS-CoV-2 RBD, SARS-CoV-2 NTD, SARS-CoV spike and SARS-CoV RBD has been described elsewhere (Cho et al., 2021; Lv et al., 2021; Yuan et al., 2020b). Briefly, the RBDs were cloned into an in-house pFastBac vector, fused with a gp67 signal peptide and an His_6 tag flanking the N- and C-terminus of the RBD. The bacmids were generated via Bac-to-Bac system (Life Technologies). The bacmid was then transfected into Sf9 cells. High (5 to 10) multiplicity of infection (MOI) of baculovirus-infected High Five cells (Life Technologies) was achieved according to the manufacturer's manual to produce RBD and spike proteins. The supernatant of the infected High Five cells was harvested around 72 h post-infection at 28°C with shaking at 110 rpm.

In addition, SARS-CoV-2 S2 subunit (R&D 10594-CV-100), HCoV-NL63 spike (Sino Biological 40604-V08B-B), HCoV-229E spike (Sino Biological 40605-V08B-B) and HCoV-HKU1 spike (Sino Biological 40606-V08B) were commercially acquired. HCoV-OC43 spike and MERS-CoV spike proteins, gifted by Prof. Andrew Ward, were synthesized as previously described (Bangaru et al., 2022). Briefly, cultures of FreeStyle 293-F cells were transfected with spike plasmid and harvested 6 d post-transfection. Complete™ His-Tag Purification Resin was used to purify spike proteins from supernatants, followed by further purification with Superose 6 increase (S6i) 10/300 column (GE Healthcare Biosciences).

The cloning, expression and purification of the recombinant HA were performed as described in previous studies (Dreyfus et al., 2012; Ekiert et al., 2009). The hemagglutinin ectodomain (11-329 of HA1 and 1-174 to HA2, in H3 numbering) from A/Solomon Islands/03/2006 (H1N1) (Ekiert et al., 2012) was linked to an N-terminal gp67 signal peptide and to a C-terminal BirA biotinylation site, thrombin cleavage site, T4 trimerization domain and 6xHis-tag of a customized pFastBac vector. Recombinant bacmid DNA was generated using the Bac-to-Bac system (Life Technologies). The bacmid was transfected into Sf9 cells using FuGENE HD (Promega) to generate baculovirus. The baculovirus was subsequently used to infect High Five cells (Life Technologies) at the MOI of 5 to 10. High Five cells were then incubated at 28°C and shaking at 110 rpm for 72 h for HA expression. The recombinant HA was purified by Ni-NTA resin followed by size exclusion chromatography, buffer exchanged into 20 mM Tris, 150 mM NaCl, pH 8.0, and concentrated for the binding assay.

Generation of multiplexed CoV antigen beads

A panel of antigens was designed consisting of recombinant SARS-CoV-2 spike, SARS-CoV-2 RBD, SARS-CoV-2 NTD, SARS-CoV spike and SARS-CoV RBD; MERS-CoV spike, HCoV-OC43 spike, H1 HA, HCoV-NL63 spike, HCoV-229E spike, HCoV-HKU1 spike, and CD4 as negative control (gifted by Prof. Gavin Wright (Crosnier et al., 2013)), all His-tagged or biotinylated. Fluorescently labelled streptavidin beads (Spherotech SVFA-2558-6K, SVFB-2558-6K, SVFA-2552-6K and SVFB-2552-6K) were incubated with 2 μg/mL anti-His biotin (Invitrogen, MA1-21315-BTIN) at room temperature for 20 min, washed with 0.5% BSA w/v in PBS, and subsequently incubated with 10 μg/mL antigens. Incubation was carried out such that each antigen was bound to beads of a distinct fluorescence intensity, resulting in a discrete fluorescence peak for each antigen. Following incubation, all beads were washed with 0.5% BSA w/v in PBS and incubated with 10 μg/mL CD4 to block any excess streptavidin sites to minimize non-specific binding to beads. Beads were then washed twice with 0.5% BSA w/v in PBS, and finally intermixed to generate multiplexed configurations.

Sequence alignment of coronaviruses

To evaluate the conservation of the primary protein structure of spike, a multiple sequence alignment was performed using the following full-length sequences: Embecovirus: Mouse Hepatitis Virus (UniProt: P11224), HCoV-OC43 (UniProt: P36334), HCoV-HKU1 N5 (UniProt: Q0ZME7), Sarbecovirus: BatCoV-HKU3 (UniProt: Q3LZX1), BatCoV-RaTG13 (GenBank: QHR63300), BatCoV-Rs4231 (GenBank: ATO98157), BatCoV-Rs3367 (GenBank: AGZ48818), BatCoV-WIV1 (GenBank: AGZ48831), Civet-SARS-CoV-007/004 (GenBank: AAU04646), Pangolin-CoV-GX-P2V (GenBank: QIQ54048), SARS-CoV-Tor2 (GenBank: AAP41037), SARS-CoV-Urbani (GenBank: AAP13441), SARS-CoV-2 Wuhan-Hu-1 (GenBank : YP_009724390), B.1.1.7 (GenBank: QWE88920), B.1.351 (GenBank: QRN78347), P.1 (GenBank: QVE55289), B.1.617.2 (GenBank: QWK65230), BA.1 (GenBank: UFO69279), BA.2 (GenBank: UJE45220), BA.2.75 (GenBank: UTM82166.1), BA.5 (GenBan: UOZ45804.1), Bat-CoV-HKU4 (UniProt: A3EX94), BatCoV-HKU5 (GenBank: YP_001039962.1), MERS-EMC/2012 (GenBank: YP_009047204), Nobecovirus: BatCoV-GCCDC1 (GenBank: YP_009273005.1), BtRt-BetaCoV/GX2018 (GenBank: QJX58383.1), BatCoV-HKU9 (GenBank: ABN10911), Hibecovius: Bat Hp-betacoronavirus/Zhejiang2013 (GenBank: YP_009072440). Sequences were aligned using the MAFFT v7 server using a BLOSUM62 scoring matrix and L-INS-I algorithm. The sequence alignment was used to generate a sequence logo plot using the Weblogo 3.0 server (Crooks et al., 2004; Schneider and Stephens, 1990) and to color conserved amino acid residues on a full-length spike protein (PDB: 7N1Q) using Chimera X.

For analysis of mutation frequencies at positions F1148, L1152 and F1156 of the SARS-CoV-2 spike protein, 5,604,512 high-quality SARS-CoV-2 spike sequences from the GISAID database (https://gisaid.org/; Complete, High Coverage options selected) were retrieved on August 24, 2022. Sequences with multiple stop codons were excluded from the mutant count.

Memory B cell isolation from PBMCs

Cryopreserved PBMCs from COVID-19 convalescent donors were thawed and stained with DAPI (BD564907), CD14-BV510 (BioLegend 301842), CD3-BV510 (BioLegend 317332), CD56-BV510 (BioLegend 318340), CD19-ECD (Beckman Coulter IM2708U), CD21-BV711 (563163), IgA-Alexa Fluor 647 (Jackson Immunoresearch 109-606-011), IgD-PE-Cy7 (BD 561314), IgM-PerCP-Cy5.5 (BD561285), CD27-Alexa Fluor 488 (BioLegend 393204) and CD38-APC-Cy7 (BioLegend 303534). Stained cells were then sorted using the BD FACSAria IIIu in a BSL3 facility. This procedure involved gating out all but live $CD19^{+}CD14^{-}CD3^{-}CD56^{-}IgM^{-}IgD^{-}$ cells, and then gating on IgA to yield purified populations of IgA-producing and IgG-producing memory B cells (MBCs).

Optofluidic-based isolation of B cells

Flow-sorted MBCs ($CD19^{+}$ IgA^{+}/IgG^{+}) were mixed with irradiated feeder cells (irr-3T3-CD40L cells). 100 μL of this cell suspension was dispensed to each well of a 384-well plate (50 MBCs, 3000 feeders per well), and cultures incubated at 37°C and 5% CO_2 for 10 d. On day 9, culture supernatants were collected and analyzed for reactivity against multiplexed CoV antigen beads by flow cytometry. From these data, culture wells of interest were specified. On day 10, MBCs from these wells of interest were pooled. These cells were washed in MACS buffer (0.5% w/v BSA in PBS with 2mM EDTA), and approximately 2.3×10^4 cells were loaded onto an OptoSelect 11k chip (Berkeley Lights). This chip was loaded into a Beacon analyzer and each individual B cell sorted into its own nanoliter-volume pen by the action of OEP light cages. 7 μm streptavidin beads (Spherotech, SVP-60-5) coated with 10 μg/mL of both MERS-CoV spike and OC43-CoV spike were incubated with 2.5 μg/mL goat anti-human IgG-Alexa Fluor 647 (Jackson Immunoresearch 109-606-170) and goat anti-human IgA-Cy3 (Jackson Immunoresearch 109-166-011). These beads were then immobilized in the channels of the OptoSelect 11k chip. Binding of secreted antibody from penned MBCs to the beads was detected in the CY5 channel (indicating IgG binding) or the TRED channel (indicating IgA binding); images from these channels were captured at 6 min intervals over a 30 min total time course. In a second step of this assay, MERS/OC43 beads were washed out of the chip and replaced with beads bound instead to SARS-CoV-2 spike that were otherwise prepared in the same manner. Antibody binding was again monitored by fluorescent image capture. This two-step procedure allowed for the identification of MBCs producing *bona fide* cross-reactive antibodies. These select MBCs were exported out of pens, again by the action of OEP light cages, and delivered directly into individual wells of a 96-well plate, where they were immediately lysed by Dynabeads mRNA DIRECT lysis buffer (Life Technologies, 61011). Plates were sealed, snap-frozen on dry ice, and placed in a -80°C freezer until required.

mAb sequence analysis and expression

RT-PCR was performed on MBC lysates to amplify heavy and light chain sequences (Cho et al., 2021; Wang et al., 2020; Tiller et al., 2008) of cross-reactive antibodies (PCR primers from Wang et al., 2020). Sequences were then resolved by Sanger Sequencing (Eurofins and Genewiz). The software Geneious Prime (Version 2021.0.3, https://www.geneious.com) was then used for analysis of VH and Vλ/Vκ genes, CDR3 sequences, and percentage of somatic mutations, with reference to the International Immunogenetics Information System database (IMGT) (Lefranc, 2014). VJ-germline sequences were obtained by reverting the V and J genes to the closest germline based on the IMGT database. The chord diagram showing the relationship between antibody and light chains was generated using the circlize package in R (Gu et al., 2014). Pairs of VH and Vλ/Vκ sequences were matched and subsequently commercially cloned into plasmids containing an IgG1 or relevant light chain backbone, and expressed as recombinant antibody (Genscript). mAbs were also expressed in-house by transient transfection of Expi293 cells (ThermoFisher Scientific, A14527) using the ExpiFectamine 293 Transfection Kit (ThermoFisher Scientific, A14524) according to manufacturer's instructions. These recombinant antibodies were purified using HiTrap Protein A columns (Cytiva/GE Healthcare Life Sciences, 17040303). Sequence alignment of CDR3 heavy and light chains was performed using MAFFT v7 server using a G-INS-I algorithm. Amino acid residues were colored according to physicochemical properties. COV44-26, COV44-54 and COV44-74 were determined to be the same lineage based on the following criteria: same heavy chain and light chain V genes, >90% identity in CDR3 amino acid sequence. Lineage analysis, including inference of the unmutated common ancestor (UCA) and putative intermediates of the COV44-26, COV44-54 and COV44-74 clonal family, was performed using Cloanalyst (Kepler, 2013). For inferred lineage members containing the ambiguous nucleotide r, the nucleotide g was used (matching the germline) to allow translation and expression as recombinant antibodies. Lineage trees were visualized using the Interactive Tree of Life (iTOL) (Letunic and Bork, 2021).

HLA typing of donor cDNA

HLA typing was necessary to identify the source of mAbs isolated from screens of donor-pooled B cells. Amplified cDNA from single cell isolates was subjected to an amplicon-based sequencing by synthesis approach using a commercially available ScisGo®-HLA-v6 kit (Scisco Genetics Inc., Seattle WA). This protocol uses a two-stage amplicon-based PCR for locus amplification and sample barcoding. Although this kit is designed for amplification from genomic DNA, a portion of kit amplicons was functional to amplify product from cDNA. Briefly, samples were sequentially subjected to two-stage PCR amplification following manufacturer's instructions, after which reactions were combined, purified, and applied to a MiSeq using Illumina Version 2 chemistry with 500-cycle,

paired-end sequencing (Illumina, San Diego, CA). Data were assembled and analyzed using specially-adapted Sciscloud® (Scisco Genetics Inc., Seattle WA) computational tools for the assembly of HLA genomic sequences derived from the ScisGo®-HLA-v6 kit. This software was made available as part of the kit. HLA class I and II genes could then be compared with typing data taken for each donor prior to sample processing, allowing for unambiguous identification of corresponding samples.

mAb binding to coronavirus antigens

Four-fold serial dilutions of recombinant mAbs in 0.5% BSA w/v in PBS, for a final dilution series of 47.7 pg/mL - 200 μg/mL, were incubated with multiplexed CoV antigen beads at room temperature for 30 min. Beads were then washed and stained with 2.5 μg/mL goat anti-human IgG Alexa Fluor 647 (Jackson Immunoresearch, 109-606-170). Samples were acquired on the iQue Screener Plus (Intellicyt) and resulting data were analysed with FlowJo (Version 10.8.1). Titration curves and AUC analyses were performed on GraphPad Prism (Version 9.3.1); values were reported after subtraction of binding values to the negative control antigen CD4. For binding of mAbs to the SARS-CoV-2 spike and stem helix peptide (peptide 154), the L9 negative control curves are the same in Figures S3B and S3E.

V gene usage survey

The iReceptor database (https://gateway.ireceptor.org/home) was surveyed to assess the frequency of circulating B cells expressing VH and VL genes of interest in healthy human donors. Study data were queried and downloaded from the AIRR Data Commons (Christley et al., 2020) using the iReceptor Gateway (Corrie et al., 2018). Only healthy donors with large datasets ($\geq 1 \times 10^6$ sequences) were included, from a total of five studies (Briney et al., 2019; DeKosky et al., 2015; DeKosky et al., 2016; DeWitt et al., 2016; Tipton et al., 2015).

Epitope binning by SPR

For epitope binning, cross-reactive mAbs were coupled to a HC30M chip (Carterra) and analysed by the Carterra LSA. The running buffer used was 0.05% BSA w/v in HEPES-buffered saline with Tween-20 and EDTA (HBSTE). Chip conditioning involved successive injections of 50 mM NaOH, 500mM NaCl and 10mM glycine pH 2, before priming with MES supplemented with 0.05% Tween. The primed chip was then activated with a 1:1 mixture of 400 mM EDC and 100 mM NHS (ThermoFisher Scientific) immediately prior to direct coupling of 10 μg/mL of mAbs in pH 4.5 acetate buffer onto discrete spots on the chip. Excess chip binding sites were blocked with 1M ethanolamine, pH 8.5. 100 nM SARS-CoV-2 S2 subunit was pre-mixed in a 1:1 ratio with 2 μM of each sandwiching antibody and the mAb-spike complexes were then injected onto the array. After each sandwiching antibody injection, the chip was regenerated by three successive injections of 10 mM glycine pH 2.0. Binning data were analyzed with Epitope Software (Carterra).

SARS-CoV-2 S2 binding kinetics

Fab fragments were prepared using the Pierce Fab Preparation kit (Thermo Fisher Scientific, 44985) following the manufacturer's protocol with slight modifications. Briefly, 250-500 μg of each cross-reactive mAb was digested using immobilized papain for 3 h at 37°C. The resulting digest was applied to Protein G Hi-Trap spin columns (Cytiva) to purify Fabs from Fc fragments and undigested mAbs. Residual reducing agent was removed using a Zeba Desalting Column 7K MWCO (ThermoScientific, 89882). Protein concentrations were determined using A280 measurements and Fab digests were confirmed using reducing and non-reducing SDS-PAGE.

For analysis of antibody binding kinetics, Fabs were coupled to an HC30M chip (0.56 μg/mL) and a three-fold dilution series of SARS-CoV-2 S2 subunit or spike protein was injected in ascending concentration without regeneration. A 10 min association and 30 min dissociation time were used. Association (k_a) and dissociation rates (k_d), as well as dissociation constants (K_D) were calculated using the Kinetics Software (Carterra).

SARS-CoV-2 S2 peptide mapping

Lyophilized 15-mer peptides that carried an N-terminal biotin tag with 12 amino acid overlap were synthesized (JPT Peptide Technologies) to span the SARS-CoV-2 S2 subunit (Ser686 - Lys1211, Accession #YP_009724390.1). Additionally, eight biotinylated peptides from H1 haemagglutinin protein were included as negative controls. 1 mg/mL peptide stocks were prepared in DMSO, then peptides were diluted to 0.1 μg/mL in 0.05% BSA w/v in HBSTE and captured onto SAD200M streptavidin-coated chips (Carterra). Cross-reactive mAbs were successively injected onto the peptide array at 10 μg/mL and regenerative binding was measured with 5 min association phase followed by a 1 min dissociation phase. Regeneration was achieved using three successive injections of 10 mM glycine pH 2.0 following each antibody injection. Data were analyzed using the Epitope Software (Carterra). To perform alanine scan experiments, the wild-type sequence $_{1142}$QPELDSFKEELDKYFKNHTS$_{1161}$ and variants with alanine substitutions at each amino acid position were synthesized with modifications described above. Biotinylated peptides were captured to a streptavidin-coated chip (Carterra) and regenerative binding was measured as described above.

Fab expression for crystallization

The variable domains of heavy chain (VH) and light chain (VL) of COV89-22, COV72-37 and COV30-14 were codon optimized (Genscript) and fused with an N-terminal secreting signal peptide and a human Fab expressing vector. The Fab was expressed by co-transfection of heavy and light chain plasmids at a 2:1 ratio (in weight) in the ExpiCHO expression system (Life Technologies) according to the Max Titer protocol in the manufacturer's manual. Supernatants were harvested, centrifuged, and purified with

CaptureSelect CH1-XL resin (Life Technologies). The eluent was purified by size exclusion chromatography (SEC) in 20 mM Tris buffer with 150 mM NaCl at pH 7.4 (TBS). Prior to crystallization trials, Fabs were concentrated to a final concentration of at least 10 mg/mL.

Crystallization and structural determination

Peptides 154 and 155 were synthesized by Genscript. The complexes of Fab of COV89-22 with peptide 154, COV30-14 with peptide 154, and COV93-03 with peptide 155 were formed by mixing Fab with a 10-fold molar ratio of peptide and incubated overnight at 4°C without additional size exclusion chromatography (SEC). The complex was subsequently adjusted to ~10 mg/mL in TBS buffer, pH 7.4. The complex was screened for crystallization on our robotic high-throughput CrystalMation system (Rigaku) at The Scripps Research Institute using the JCSG Core Suite (QIAGEN) as precipitant. Crystallization trials were setup by the vapor diffusion method in sitting drops containing 0.1 μL of protein and 0.1 μL of reservoir solution. The optimized crystallization condition for COV89-22 with peptide 154 was 0.1 M sodium citrate, pH 5.6, 20% 2-propanol, and 20% PEG4000. The optimized condition for COV30-14 with stem helix peptide was 0.2 M sodium chloride; 2 M ammonium sulfate, and 0.1 M sodium cacodylate, pH 6.5 and, for the COV93-03-peptide complex, was 0.2 M sodium chloride, 30% PEG3000, and 0.1 M Tris pH 7. Crystals were harvested on or before Day 14 and then soaked in reservoir solution with 15% (v/v) ethylene glycol as the cryoprotectant. The harvested crystals were flash-cooled and stored in liquid nitrogen until data collection. Diffraction data were collected at cryogenic temperature (100 K) at the Stanford Synchrotron Radiation Lightsource on Scripps/Stanford beamline 12-1 with a beam wavelength of 0.97946 Å for the COV89-22-peptide complex, and at beamline 23-ID-B of the Advanced Photon Source (APS) with a beam wavelength of 1.033167 Å for the COV30-14-peptide and COV93-03-peptide complexes. The diffraction data were processed with HKL2000 (Otwinowski and Minor, 1997). The complex structure was solved by molecular replacement using Phaser (McCoy et al., 2007) with the models generated by Repertoire Builder (https://sysimm.org/rep_builder/) for COV89-22. Iterative model building and refinement were carried out in Coot (Emsley et al., 2010) and PHENIX (Adams et al., 2010), respectively. Ramachandran statistics were obtained from MolProbity (Chen et al., 2010). Buried and accessible surface areas were calculated with PISA (Krissinel and Henrick, 2007). Molecular surface contact areas were computed using the Molecular Surface package (Connolly, 1983). A similar workflow was performed for COV30-14 with peptide 154 and COV93-03 with peptide 155.

Microscopy-based fusion inhibition assay

HeLa cells were transduced with lentiviral vectors encoding both (NLS)-RFP (a nuclear localization signal) and the spike protein for SARS-CoV, SARS-CoV-2 or MERS-CoV. These cells were then stained with corresponding mAbs and sorted for successfully transduced (RFP^{high}/$Spike^{high}$) cells. A separate population of HeLa-hACE2 cells were transduced with GFP-encoding lentivirus and sorted for GFP^{high}/$ACE2^{high}$ cells. Spike-expressing HeLa cells were seeded in 96 well plates (5×10^3 cells per well) overnight, and then treated for 1 h with 200 μg/mL mAbs before addition of 8×10^3 GFP^+/$ACE2^+$ HeLa cells per well. These co-cultures were maintained overnight to facilitate syncytia development. Cultures were then microscopically evaluated for syncytia. Cells were fixed in 4% PFA for 15 min, washed twice with PBS, counter-stained with 1 μg/mL Hoechst for 10 min, and washed twice more with PBS. A488, A568 and DAPI fluorescence were measured using a BZ-X fluorescence microscope (KEYENCE) and the images were processed using Fiji ImageJ (Schindelin et al., 2012).

Quantitative fusion inhibition assay

Acceptor cells were derived from HEK-293 cells and engineered to stably express TMPRSS-2 and hACE2, as well as secreted embryonic alkaline phosphatase (SEAP) under control of the NF-κb promoter (Invivogen, hkb-hace2tpsa). Donor cells were also derived from HEK-293 and engineered to stably express hMyD88 (Invivogen, 293-hmyd). Initially, donor and acceptor cells were cultured in growth media comprised of high glucose (4.5 g/L) DMEM supplemented with 4 mM L-glutamine, 100 U/mL penicillin/streptomycin, 10% HI-FBS, and 100 μg/mL Normocin. After 2 passages, acceptor cells were cultured in growth media supplemented with 0.5 μg/mL Puromycin, 100 μg/mL Zeocin, and 200 μg/mL Hygromycin B Gold while donor cells were cultured in growth media supplemented with 10 μg/mL Puromycin. Cells were cultured and incubated at 37°C, 5% CO_2 for all steps unless otherwise indicated.

For fusion inhibition, 9×10^5 donor cells were seeded into 6-well cell culture plates overnight. Transfection complexes comprising 3:1 ratio of PEIMax and SARS-CoV-2 spike plasmid or vector control was added directly to donor cells overnight. 5×10^4 donor cells transfected with SARS-CoV-2 spike or vector control were incubated for 1 hour with 3-fold serial dilutions of each antibody. 5×10^4 acceptor cells were mixed with donor cells and antibody overnight and the following day 100 μL of supernatant was removed from each well and transferred to a 96-well flat bottom plate. 100 μL/well of Quantiblue substrate, prepared as directed by the manufacturer, was added to each well and incubated for 3 hours. SEAP activity was measured using Abs_{635} using an Enspire Multimode plate reader. Absorbance measurements were background corrected by subtracting the absorbance of donor cells transfected with the vector control (N). % Inhibition was determined using the formula $100 \times (1 - (E - N) / (P - N))$; E – Abs_{635} of test mAb and P – Abs_{635} of 0 μg/mL mAb.

Shotgun mutagenesis epitope mapping

Epitope mapping was conducted as described previously (Davidson and Doranz, 2014). A shotgun mutagenesis mutation library for the S2 subunit of SARS-CoV-2 (Wuhan-Hu-1 strain) spike protein was made using a full-length spike glycoprotein construct. In brief, 513 residues between positions 689 and 1247 were individually mutated to alanine, while alanine residues in this sequence were mutated to serine. Following sequence confirmation, clones were individually arrayed in wells of a 384-well plate, transfected into

HEK-293T cells and expressed for 22 h. Cells were then fixed in 4% (v/v) paraformaldehyde (Electron Microscopy Sciences) and permeabilized for intracellular staining with 0.1% (w/v) saponin (Sigma-Aldrich) in PBS. Fixed, permeabilized cells were then treated with mAbs diluted in PBS, 10% normal goat serum (Sigma), and 0.1% saponin. Optimal mAb treatment concentration had previously been determined by immunofluorescence titration curves against cells expressing wild-type spike protein. Primary mAbs were detected using 3.75 μg/mL of Alexa-Fluor-488-conjugated anti-IgG (Jackson ImmunoResearch) in 10% normal goat serum with 0.1% saponin. Stained cells were then washed three times with PBS/0.1% saponin and two times with PBS, and analyzed by high-throughput flow cytometry (Intellicyt iQue, Sartorius). Wild-type spike protein-transfected cells and mock-transfected cells were both used as controls, and antibody binding to each mutant spike clone was calculated by subtraction of the signal for mock-transfected cells and normalization to the signal from wild-type spike-transfected controls. Residue mutations were deemed critical to the mAb epitope if they did not support reactivity of the test mAb but did support the reactivity of control SARS-CoV-2 antibodies (as described in the text), in order to exclude locally misfolded spike mutants or those with expression defects.

Authentic HCoV-OC43-GFP neutralization assay

Rhabdomyosarcoma (RD) cells (ATCC CCL136) were suspended in DMEM (Gibco, 11966) supplemented with, 10% HIFBS, 4500 mg/mL glucose, 1 mM sodium pyruvate, 1 mM HEPES and 50 μg/mL gentamicin (Quality Biological, 120-098-661), and cultured in a T225cm^2 flask at 37°C and 5% CO_2 for 24 h to achieve 90% confluency. For virus propagation, cells were washed with PBS, resuspended in FBS-free glucose-high DMEM, supplemented with 1× GlutaMax (Gibco, 11965-092) and sodium pyruvate, and infected with GFP-expressing HCoV-OC43 at an MOI of 0.01. Total assay volume was 10 mL, and infections were carried out at 35°C for 1 h, with gentle rocking at 10 min intervals. Following this duration, the 10 mL infection mix was replaced with 35 mL glucose-high DMEM, supplemented with 1× Glutamax, 1× nonessential amino acids (Gibco, 12491-015), 2% HIFBS, 15 mM HEPES and 50 μg/mL gentamicin. The resulting culture was maintained at 35°C and 5% CO_2. At 3 – 4 d post-infection, cultures were centrifuged at 234 × g for 30 min at 4°C, and virus-containing supernatants collected and stored at -80°C. $TCID_{75}$ (the volume of virus required for 75% infection) of RD cell cultures was determined by endpoint dilution.

For neutralization assays, RD cells were seeded at 5 × 10^4 cells per well in 96-well round-bottomed plates and rested at 37°C. Media formulations of serially diluted mAbs and OC43-GFP virus were incubated for 1 h at 37°C, after which media in wells of RD cells was aspirated and replaced with 60 μL of the mAb/virus mix. RD cells were subsequently incubated for 24 h at 37°C. Each mAb dilution was plated in duplicate. Untreated uninfected cells were plated as negative controls, and untreated infected cells plated as positive controls (Min_{GFP} and Max_{GFP}, respectively). GFP expression was measured as before and % neutralization calculated as $100 \times (1 - (GFP - Min_{GFP}) / (Max_{GFP} - Min_{GFP}))$.

Pseudovirus neutralization assay ($Assay_{NIH}$)

Codon-optimized cDNA encoding spike protein from a panel of coronaviruses (SARS-CoV-2 (GenBank: QHD43416.1), SARS-CoV (Urbani; GenBank: AAP13441.1), MERS-CoV (EMC; GenBank: AFS88936) and HCoV-NL63 (GenBank: Q6Q1S2.1)) were synthesized (Genscript) and cloned into a mammalian expression vector (VRC8400) (Barouch et al., 2005). These constructs were confirmed by sequencing. To generate pseudovirions expressing coronavirus spike proteins, HEK-293T cells were transfected with the packaging plasmid pCMVdR8.2, transducing plasmid pHR' CMV-Luc, a TMPRSS2 plasmid (Wei et al., 2010) and coronavirus spike plasmid using the Lipofectamine 3000 transfection reagent (ThermoFisher Scientific, Asheville, NC, L3000-001) (Naldini et al., 1996). For neutralization assays, pseudovirus bearing spike proteins from SARS-CoV-2, SARS-CoV and HCoV-NL63 was incubated with 293 flpin-TMPRSS2-ACE2 cells (Zhou et al., 2022c), while HuH7.5 cells were used for MERS-CoV pseudovirus (Wang et al., 2015). 7.5 × 10^4 cells were plated per well of a 96-well white/black Isoplate (PerkinElmer, Waltham, MA) and incubated overnight before infection with pseudovirus. Serial dilutions of cross-reactive mAbs were mixed with pseudovirus and incubated at 37°C for 45 min. mAb-pseudovirus complexes were then added to cells in triplicate. Cultures were incubated for 2 h, at which point wells were refreshed with fresh media and cultures maintained for a further 72 h, before cells were lysed and luciferase activity measured with Microbeta (Perkin Elmer).

Pseudovirus neutralization assay ($Assay_{Scripps}$)

Production of lentiviral based pseudoviruses was performed as described previously (Rogers et al., 2020). HEK-293T cells were co-transfected with 2.5 μg 2nd generation lentivirus backbone plasmid pCMV-dR8.2 dvpr (Addgene #8455), 2 μg pBOBI-FLuc (Addgene #170674) and 1 μg truncated coronavirus spike expressing plasmids (SARS: Addgene #170447; SARS2 #170442; MERS #170448; NL63 #172666; alpha variant #170451; beta #170449; gamma #170450; delta #172320; BA.1 #180375; BA.2 #183700; BA.2.12.1 #186809; BA.2.75 #190674; BA.4/5 #186810) using Lipofectamine 2000 (ThermoFisher Scientific, 11668019). Media was refreshed at 12-16 h post transfection. At 48 h and 72 h post transfection supernatants were collected, centrifuged at 1,500 × g for 10 min, and the viral titers measured by luciferase activity in relative light units (RLU) (Bright-Glo Luciferase Assay System, Promega, E2620). Supernatants were stored at -80°C until ready for use. Pseudotyped viral neutralization assays were based on previously described methods (Rogers et al., 2020). Serial dilutions of cross-reactive mAbs were mixed with pseudovirus supernatants and incubated at 37°C for 1 h. HeLa-hACE2 cells, suspended in a 30 μg/mL Dextran media, were then added to mAb-pseudovirus complexes, at a density of 5 × 10^3 cells per well. Cultures were incubated for 42-48 h before cells were lysed and luciferase activity measured. The 50% neutralization titer (NT_{50}) for each antibody was calculated using the dose-response-inhibition model with 5-parameter Hill slope equation in GraphPad Prism version 9.3.1.

Spike mutant generation and infectivity assay

F1148A, L1152A, F1156A and F1148A/L1152A/F1156A plasmids were constructed based on WT pcDNA3.3_CoV2_D18 (Addgene #170442). The vector plasmid was digested with BbvCI and XhoI (NEB), and insertion fragments were PCR amplified with the primers as listed in Table S3.

The fragments were gel separated, recycled, and ligated by NEBuilder HiFi DNA Assembly. Mutant plasmids from single colonies were miniprepped and sequenced to ensure correctness.

The lenti-based pseudotyped viruses were packed as previously described. WT, 1148A, 1152A, 1156A and F1148A/L1152A/F1156A pseudovirus were produced side-by-side in one batch of HEK293T and the supernatants were collected and frozen in -80°C for later use. Viral particle titers were measured by Lenti-X p24 Rapid Titer Kit (Takara, 632200) following manufacturer's instructions. The relative infectivity (RI) of WT and mutant pseudoviruses were calculated by the following equation: (assuming WT=1)

$$RI = \left[RLU^{mut}/n\left(p24^{mut}\right)\right] \div \left[RLU^{wt}/n\left(p24^{wt}\right)\right]$$

Authentic SARS-CoV-2 neutralization assay

On the day before infection, Vero E6 cells (Expasy CVCL_XD71) were resuspended in complete DMEM medium with 10% heat-inactivated serum, 1% GlutaMAX, 1% pen/strep and added to 96-well half-area plates at a density of 10,000 cells per well. A serial dilution of antibodies was performed and the antibodies were mixed 1:1 with SARS-CoV-2 (final concentration of 1,000 plaque forming units/well), incubated for 30 min, and added to the Vero cells. The infection was allowed to proceed at 37°C for 24 h. After this period, the supernatant was removed and disposed of appropriately. The Vero cells were fixed with 4% paraformaldehyde for 1 h and washed 3 times with PBS. Plates containing the cells were kept at 4°C or shaken for 30 min with 100 μL/well of permeabilization buffer (PBS with 1% Triton-X). Next, the buffer was discarded and 100 μL of 3% BSA was added to each well. The plates were then incubated for 2 h at RT. An antibody mixture including equal amounts of CC6.29, CC6.33, L25-dP06E11, CC12.23 and CC12.25 was diluted in PBS with 1% BSA to a concentration of 2 μg/mL. Next, 50 μL of this antibody cocktail was added the plates to stain SARS-CoV-2. The plates were incubated for 1 h at RT, following which the plates were washed 3 times with PBST (PBS + 0.05% Tween-20). Goat anti-human IgG conjugated to horseradish peroxidase (HRP) (50 μL of 1 ug/mL) (Invitrogen, A18817) was added to the plates, which was then incubated at RT for 1 h. The plates were washed with PBST at least 5 times and pressed on tissue paper to absorb remaining moisture. 50 μL of POD substrate was added to each well (Roche, 11582950001) according to the manufacturer's protocol and chemiluminescence intensity was measured in a plate reader. The percentage of neutralization was determined as % Neutralization = (1 - (Read – NC) ÷ (PC – NC)) x 100%, where negative control (NC) is the average intensity of negative control wells without virus, and positive control (PC) is the average of positive control wells with virus but no antibody.

Authentic MERS-CoV neutralization assay

1 d prior to infection with MERS-CoV, Vero E6 cells (BEI NR-596) were resuspended in DMEM (Gibco) supplemented with 10% FBS (Sigma) and added to 384-well tissue-culture treated plates at a concentration of 6,000 cells in 30 μL for each well. On the day of infection, a 12-point serial dilution of antibodies of interest starting from 200 μg/mL was made in quadruplicate. 30 μL volumes of the antibodies were mixed 1:1 with MERS-CoV at a concentration of 18,000 plaque forming units (PFU) per 30 μL (final starting antibody concentration, 100 μg/mL). These mixtures were incubated at 37°C for 1 h. Next, 30 μL of each mixture was added to wells containing 30 μL of Vero E6 cells, resulting in a final assay volume of 60 μL. The cells were incubated for 24 h, following which 10% neutral buffered formalin was added to fix the samples. The plates were removed from biocontainment and an anti-MERS-CoV spike primary antibody (Sino Biological, 40069-R723) was added to the wells, followed by an Alexa Fluor 647-conjugated goat anti-rabbit antibody (Life Technologies, A21245). The cells were stained with Hoechst dye to facilitate detection of nuclei. Fluorescence signals were quantified with the Operetta imaging system (PerkinElmer). Half-maximal inhibitory concentration (IC_{50}) values were determined using GraphPad Prism as previously described (Covés-Datson et al., 2019). Z' factor scores were calculated to verify the quality of each plate.

Hamsterization of human monoclonal antibodies

Recombinant mAbs were hamsterized by replacing the human IgG1 constant region with a heavy chain locus of the Syrian hamster IgG2a using the MesAur1.0 (GenBank: GCA_00349664.1) genome assembly. To identify the Syrian hamster IgG2a heavy chain locus, an alignment was performed using the mouse IgG2a heavy chain as a reference sequence. Expi293 cells were transfected with codon-optimized hamsterized plasmids following the manufacturer's instructions with the Expifectamine transfection kit as described above.

Syrian hamster efficacy studies

Golden Syrian Hamsters acquired from Envigo (Indianapolis, IN USA) were acclimated at IRF facility for 10 d and weighed 2 d prior to study commencement. At 5-6 weeks old, equal numbers of males and females were grouped into eight groups of n = 12 according to weight and sex. The study was blinded and animals were randomly assigned antibody treatment (16 mg/kg) or mock treatment (PBS). Treatment was intraperitoneal (IP), and carried out 24 h prior to intranasal (IN) inoculation with either 5 $\log_{10}$ PFU SARS-CoV-2 (WA01)

or PBS for mock-exposed animals. Animals were weighed and observed daily for clinical signs of disease. On day 3, half of the animals in each group were sacrificed. Remaining animals were sacrificed on day 7. None of the animals used in the study reached endpoint criteria that would have required an unscheduled euthanasia. Following sacrifice, sagittal sections of the left lung lobe were obtained for histopathology. Lung tissue samples underwent fixation for 72 h in 10% neutral-buffered formalin. Next, the tissues were processed in a Tissue-Tek VIP-6 automated vacuum infiltration processor (Sakura Finetek USA). The processed samples were embedded in paraffin with a Tissue-Tek Model TEC-6 unit (Sakura Finetek USA). Tissue sections were then cut to a thickness of 4 μm using a standard semi-automated rotary microtome and lighted water flotation bath (Leica Biosystems). The samples were mounted on glass slides with a positive surface charge (ThermoFisher Scientific) and air-dried at room temperature. The samples were then stained with hematoxylin-eosin and coverslips were added onto the slides. A trained pathologist evaluated the samples by microscopy. Scoring for pathology was based on percent area affected by interstitial pneumonia in the left lung lobe (estimated lesion distribution of interstitial pneumonia as a percentage in a sagittal section of the entire left lung lobe: 0 = 0%, 1 = <25%, 2 = 26-50%, 3 = 51-80% or 4 = >80%).

Vaccinee and convalescent donor IgG binding

Polyclonal IgG were purified from plasma or sera from vaccinated, convalescent and naïve donors with a Pierce Protein G Spin Plate (ThermoFisher Scientific). Briefly, plasma and sera were diluted 1:4 in PBS before incubation with Protein G at room temperature for 30 min, with shaking at 600 rpm. Flowthrough was collected and incubated with the Protein G resin for an additional 15 min to ensure maximum binding. The protein G resin was washed four times with PBS before IgG was eluted with Protein G Elution Buffer (ThermoFisher Scientific). This buffer was neutralized with 1 M Tris pH 8.0 and buffer exchanged into PBS using a 40 kDa MWCO Zeba Plate. IgG was diluted to 100 μg/mL to assess epitope reactivity. Antibodies were added to a SAD200M chip (Carterra) with biotinylated peptide 154 as described above and binding was analyzed using the Epitope Software (Carterra).

QUANTIFICATION AND STATISTICAL ANALYSIS

50% antibody neutralization titers (NT50) values were interpolated from neutralization curves fitted using the dose-response-inhibition model of non-linear regression analysis with 5-parameter Hill slope equation. For Syrian hamster efficacy studies, average body weight was analyzed for statistically significant differences across the 7 d time-course using a mixed-effects repeated measures model with Dunnett's post-test multiple comparison. Hamster clinical and pathology scores were analyzed by Kruskal-Wallis tests with Dunn's post-test multiple comparison. Comparisons of convalescent and vaccinated polyclonal IgG binding to the stem helix were made from a nested, mixed-model ANOVA with Bonferroni-adjusted P-values. Descriptive statistics (mean ± SEM or mean ± SD) and statistical analyses were performed using Prism version 9.3.1 (GraphPad). For all analyses $^{*}P < 0.05$, $^{**}P < 0.01$, $^{***}P < 0.001$, $^{****}P < 0.0001$ and ns, not significant. Data for viral infectivity assay, $\text{Assay}_{\text{NIH}}$ pseudovirus neutralization, and shotgun alanine mutagenesis are from n = 1 experiment. NT50 values for initial screen of broad mAb panel ($\text{Assay}_{\text{Scripps}}$) are from a single screening experiment. All other data are representative of n = 2 experiments.

Cell Host & Microbe, Volume *31*

Supplemental information

Rare, convergent antibodies targeting the stem helix broadly neutralize diverse betacoronaviruses

Cherrelle Dacon, Linghang Peng, Ting-Hui Lin, Courtney Tucker, Chang-Chun D. Lee, Yu Cong, Lingshu Wang, Lauren Purser, Andrew J.R. Cooper, Jazmean K. Williams, Chul-Woo Pyo, Meng Yuan, Ivan Kosik, Zhe Hu, Ming Zhao, Divya Mohan, Mary Peterson, Jeff Skinner, Saurabh Dixit, Erin Kollins, Louis Huzella, Donna Perry, Russell Byrum, Sanae Lembirik, Michael Murphy, Yi Zhang, Eun Sung Yang, Man Chen, Kwanyee Leung, Rona S. Weinberg, Amarendra Pegu, Daniel E. Geraghty, Edgar Davidson, Benjamin J. Doranz, Iyadh Douagi, Susan Moir, Jonathan W. Yewdell, Connie Schmaljohn, Peter D. Crompton, John R. Mascola, Michael R. Holbrook, David Nemazee, Ian A. Wilson, and Joshua Tan

A

B

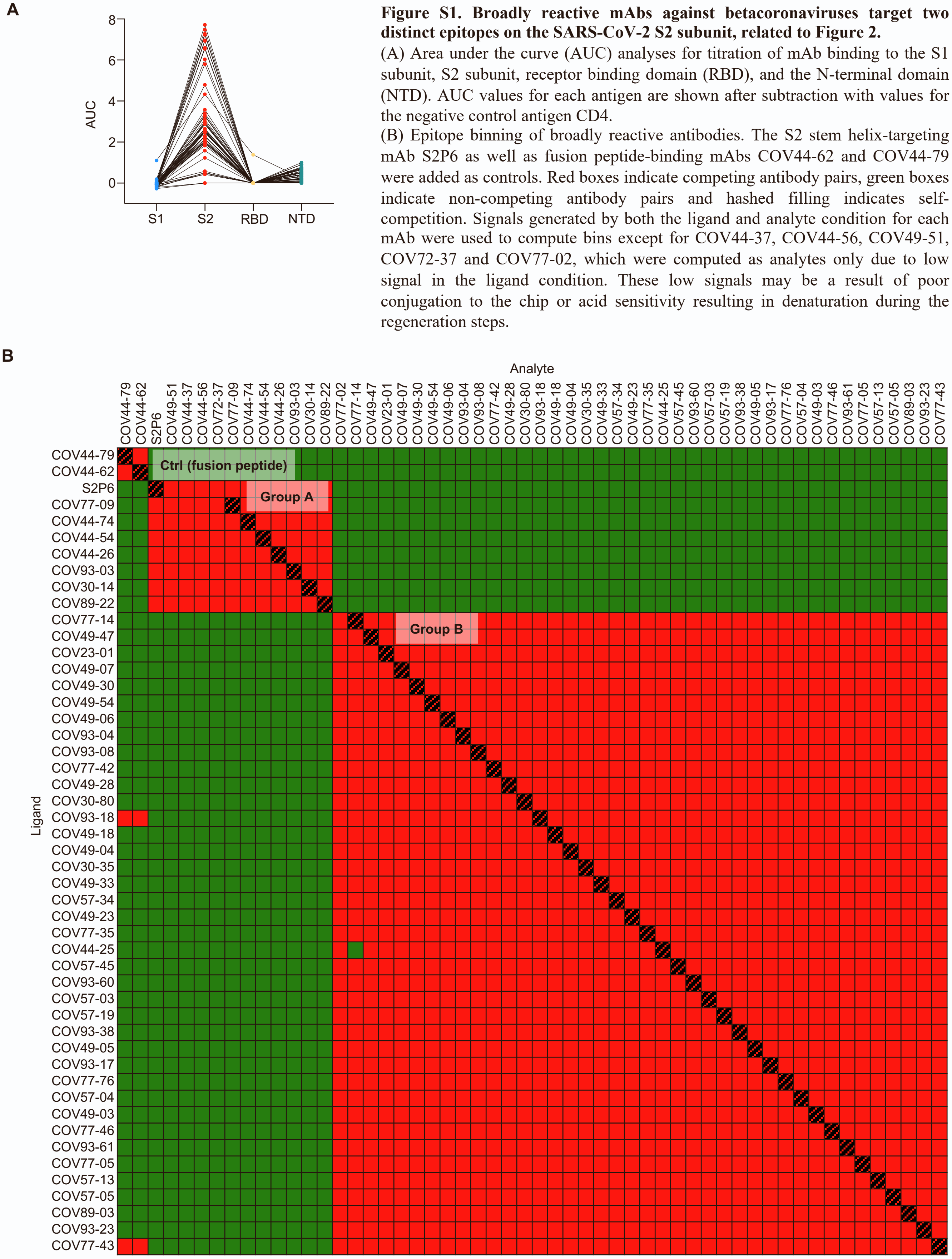

Figure S1. Broadly reactive mAbs against betacoronaviruses target two distinct epitopes on the SARS-CoV-2 S2 subunit, related to Figure 2.

(A) Area under the curve (AUC) analyses for titration of mAb binding to the S1 subunit, S2 subunit, receptor binding domain (RBD), and the N-terminal domain (NTD). AUC values for each antigen are shown after subtraction with values for the negative control antigen CD4.

(B) Epitope binning of broadly reactive antibodies. The S2 stem helix-targeting mAb S2P6 as well as fusion peptide-binding mAbs COV44-62 and COV44-79 were added as controls. Red boxes indicate competing antibody pairs, green boxes indicate non-competing antibody pairs and hashed filling indicates self-competition. Signals generated by both the ligand and analyte condition for each mAb were used to compute bins except for COV44-37, COV44-56, COV49-51, COV72-37 and COV77-02, which were computed as analytes only due to low signal in the ligand condition. These low signals may be a result of poor conjugation to the chip or acid sensitivity resulting in denaturation during the regeneration steps.

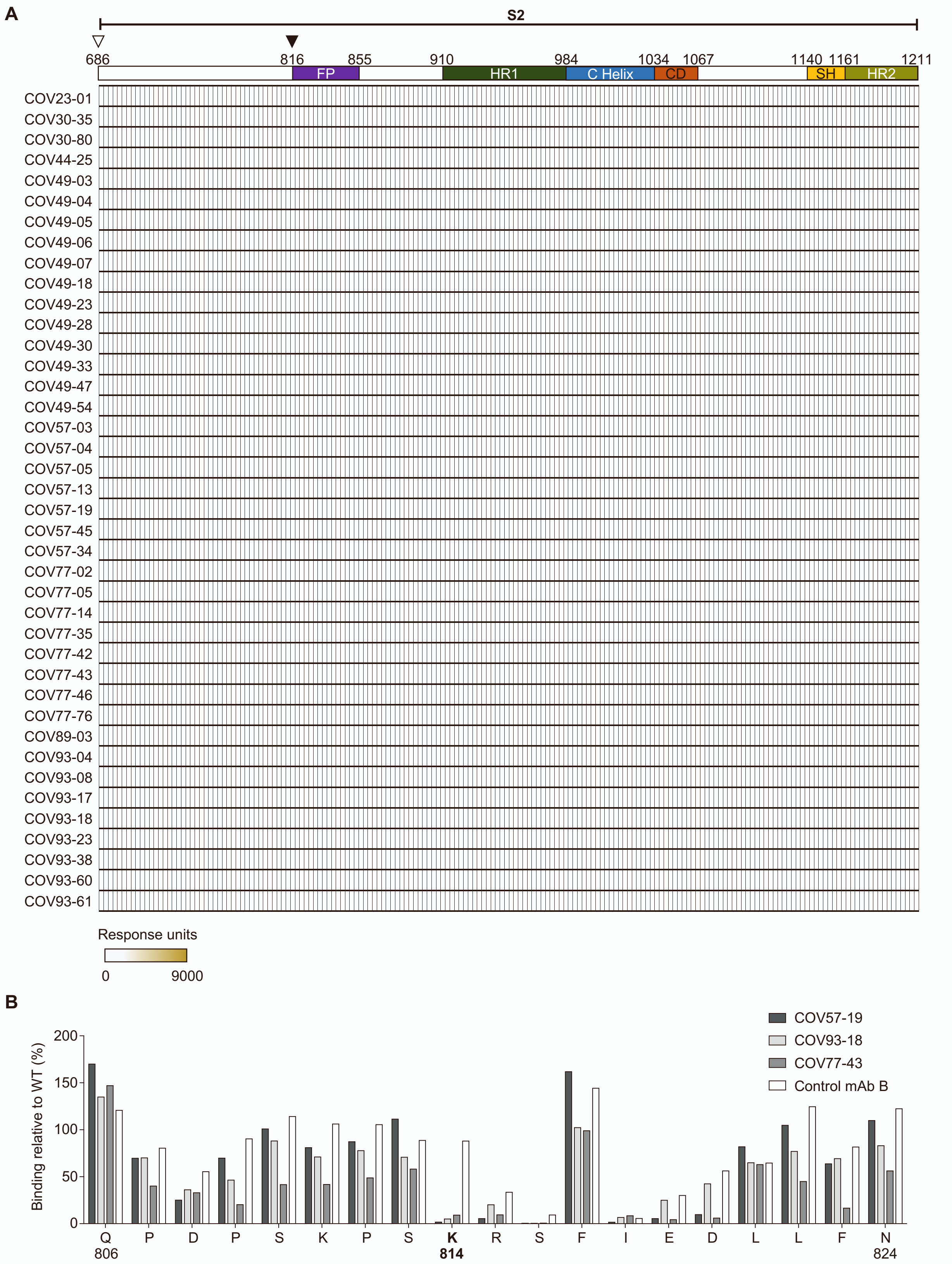

Figure S2. Group B mAbs bind to a conformational epitope that includes K814, related to Figure 2.

(A) The heat map shows the binding responses of Group B mAbs, 10 μg/mL, to an array of biotinylated peptides spanning the SARS-CoV-2 Wuhan-Hu-1 Spike S2 domain.

(B) Effect of mutations on binding of three group B mAbs in shotgun mutagenesis assay. A residue was considered critical if mutation of this residue resulted in a reduction of binding signal for the three group B mAbs but not control mAb B, which targets a conformational epitope not in this region.

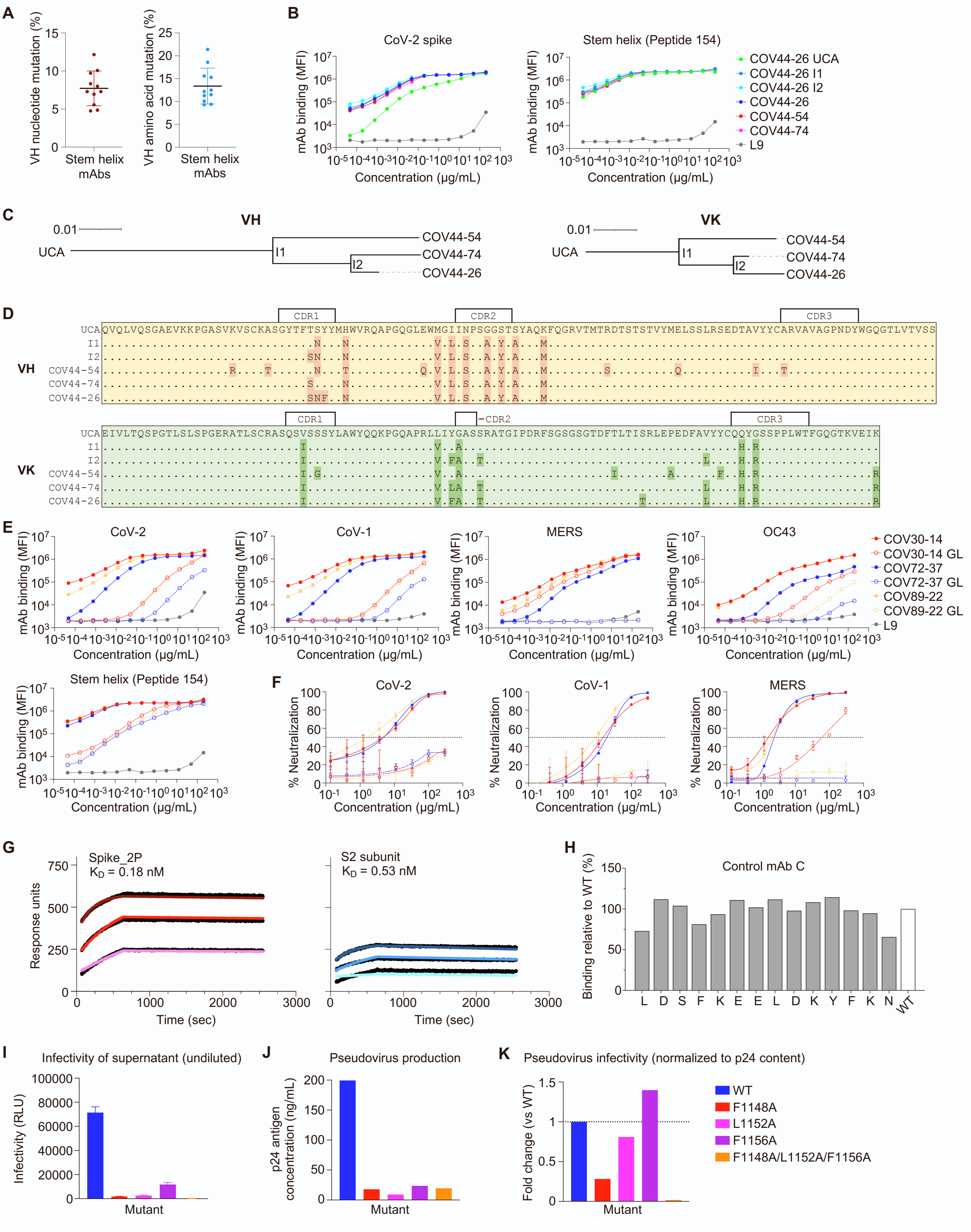
A
VH nucleotide mutation (%)
Stem helix mAbs
VH amino acid mutation (%)
Stem helix mAbs
B
CoV-2 spike
Stem helix (Peptide 154)
mAb binding (MFI)
Concentration (µg/mL)
COV44-26 UCA
COV44-26 I1
COV44-26 I2
COV44-26
COV44-54
COV44-74
L9
C
VH
VK
0.01
UCA
I1
I2
COV44-54
COV44-74
COV44-26
D
CDR1
CDR2
CDR3
UCA QVQLVQSGAEVKKPGASVKVSCKASGYTFTSYYMHWVRQAPGQGLEWMGIINPSGGSTSYAQKFQGRVTMTRDTSTSTVYMELSSLRSEDTAVYYCARVAVAGPNDYWGQGTLVTVSS
UCA EIVLTQSPGTLSLSPGERATLSCRASQSVSSSYLAWYQQKPGQAPRLLIYGASSRATGIPDRFSGSGSGTDFTLTISRLEPEDFAVYYCQQYGSSPPLWTFGQGTKVEIK
E
CoV-2
CoV-1
MERS
OC43
Stem helix (Peptide 154)
COV30-14
COV30-14 GL
COV72-37
COV72-37 GL
COV89-22
COV89-22 GL
L9
F
% Neutralization
G
Spike_2P
K_D = 0.18 nM
S2 subunit
K_D = 0.53 nM
Response units
Time (sec)
H
Control mAb C
Binding relative to WT (%)
L D S F K E E L D K Y F K N WT
I
Infectivity of supernatant (undiluted)
Infectivity (RLU)
Mutant
J
Pseudovirus production
p24 antigen concentration (ng/mL)
Mutant
K
Pseudovirus infectivity (normalized to p24 content)
Fold change (vs WT)
Mutant
WT
F1148A
L1152A
F1156A
F1148A/L1152A/F1156A

Figure S3. Characterization of stem helix-specific mAbs, related to Figure 3.

(A) Percentage mutation of VH nucleotides and VH amino acids for stem helix-specific mAbs, as inferred using IMGT (imgt.org). Error bars show mean ± SD.

(B) Binding of COV44-26-lineage antibodies to SARS-CoV-2 spike and peptide 154 from the stem helix region. L9 IgG1 (malaria-specific; [S1]) was included as a negative control mAb for binding experiments. Interconnected data points in the titration curves are shown without curve fitting.

(C) VH and VK lineage trees of the clonal family consisting of COV44-26, COV44-54 and COV44-74. The unmutated common ancestor (UCA) and intermediates (I1 and I2) were inferred using Cloanalyst [S2].

(D) Heavy and light chain sequence alignments of members of the COV44-26 lineage. CDR positions were determined using IMGT (imgt.org).

(E) Binding of VJ germline-reverted and mature COV30-14, COV72-37 and COV89-22 to spike of human coronaviruses. For the SARS-CoV-2 spike and stem helix peptide, the L9 negative control curves are the same as in Figure S3B.

(F) Neutralization of SARS-CoV-2, SARS-CoV and MERS-CoV pseudoviruses by VJ germline-reverted and mature COV30-14, COV72-37 and COV89-22. The dotted line represents 50% neutralization and error bars show mean ± SD.

(G) Kinetics of binding of COV89-22 Fab to SARS-CoV-2 spike protein, pre-fusion stabilized with two proline mutations (2P), as well as the unmodified S2 subunit of SARS-CoV-2 spike.

(H) Shotgun mutagenesis showing binding of control mAb C to residues in the stem helix region.

(I) Infectivity of undiluted supernatants containing WT SARS-CoV-2 pseudotyped virus, compared to F1148A, L1152A, F1156A (single), and F1148A/L1152A/F1156A (triple) spike mutants. RLU, relative light units. Error bars show mean ± SD.

(J) Pseudovirus production of WT SARS-CoV-2, as well as F1148A, L1152A, F1156A (single), and F1148A/L1152A/F1156A (triple) spike mutants, based on p24 antigen concentrations of each preparation.

(K) Normalized pseudovirus infectivity as calculated by ratio of infectivity in RLU to p24 antigen concentration in ng/mL, with the WT pseudovirus normalized to 1.

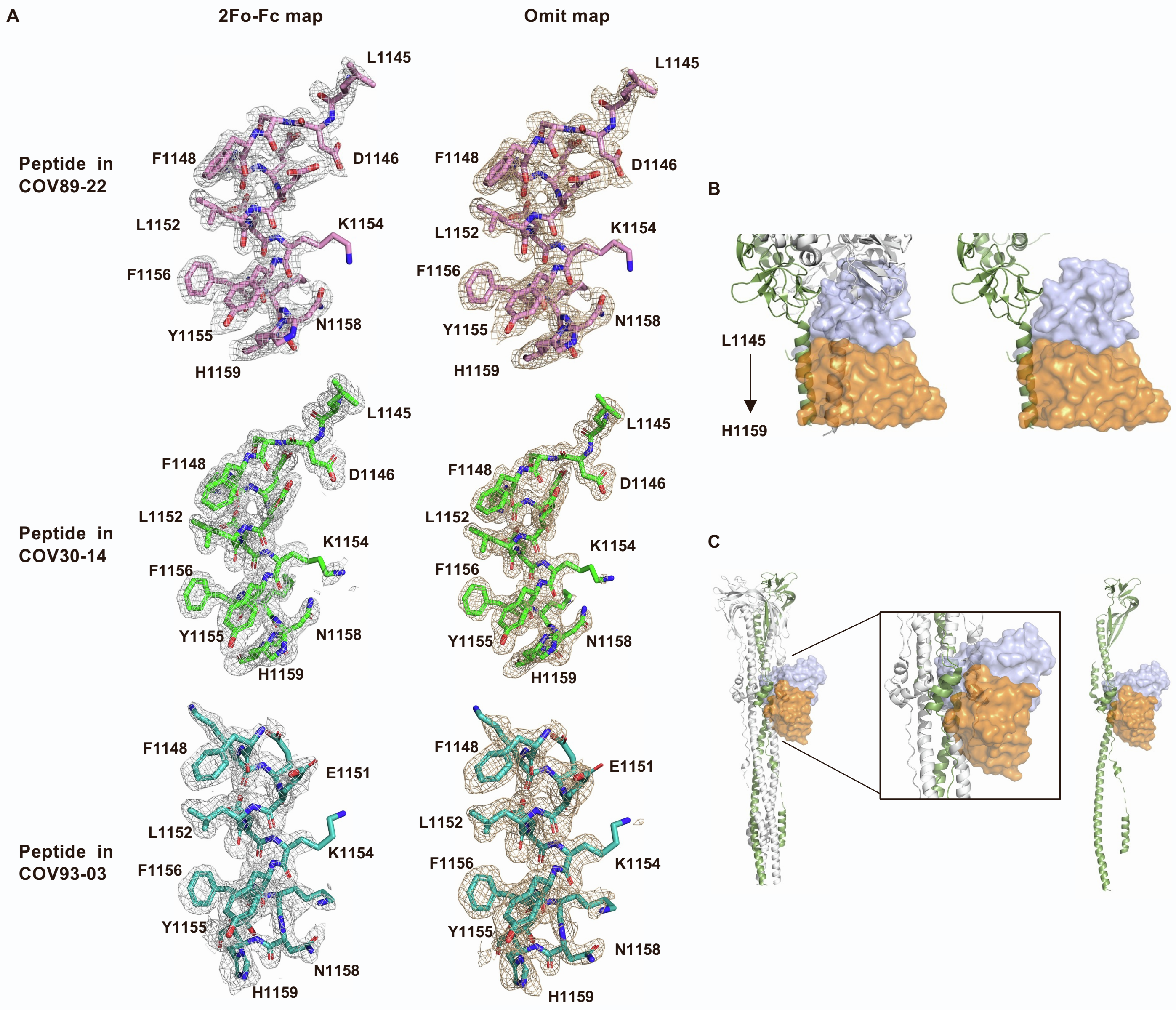

Figure S4. Electron density maps for the stem helix peptide and overall binding of COV89-22 to the stem helix peptide, related to Figures 4 and 5.

(A) The 2Fo-Fc electron density maps are represented in a gray mesh and contoured at 1.0σ for the stem helix peptide bound to COV89-22, COV30-14, and COV93-03, respectively. The Fo-Fc unbiased omit electron density maps are represented in a brown mesh and contoured at 2.0σ for each of the stem helix peptides.

(B) Location of stem helix peptide (L1145-H1159) on a protomer of the SARS-CoV-2 spike protein. Structures of COV89-22-peptide complex were superimposed onto the stem helix of an intact SARS-CoV-2 spike trimer and monomer structure in the pre-fusion state (PDB: 6XR8). The monomer shows the interaction surface of stem helix with COV89-22 which is located on the inside of three helix bundle in the stem region. COV89-22 would clash with the three-helix bundle in the stem region in the pre-fusion state. Conformational changes or conformational dynamics would be required for COV89-22 binding.

(C) The structures of the COV89-22-peptide complex was superimposed onto the stem helix of an intact SARS-CoV-2 spike trimer and monomer structure in the post-fusion state (PDB: 6XRA). Stabilization of an intermediate state would suggest a possible neutralization mechanism of COV89-22.

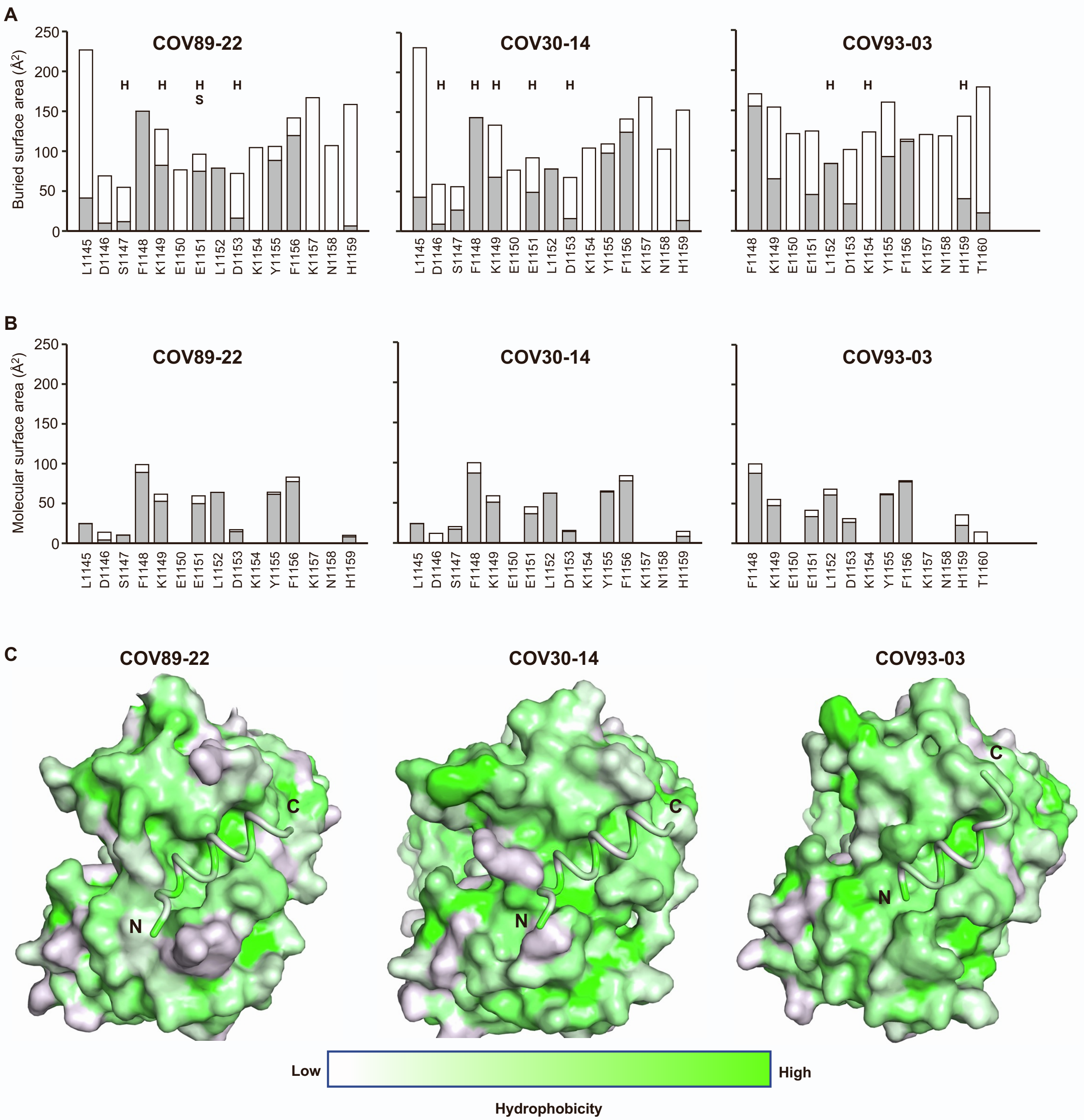

Figure S5. Buried molecular surface area and hydrophobicity of COV89-22, COV30-14, and COV93-03 in complex with SARS-CoV-2 stem helix peptides, related to Figures 4 and 5.

(A) Comparison of the peptide buried area of COV89-22, COV30-14, and COV93-03. Buried surface area (in gray) and accessible surface area (in white) of each residue of the stem helix peptide in complex with antibody is shown in the stacked bar chart. Residues of COV89-22, COV30-14 or COV93-03 that form polar interactions are denoted with "H" on top of each bar if they form a hydrogen-bond or "S" if they form a salt bridge. Buried and accessible surface areas were calculated with PISA [S3].

(B) Comparison of the peptide molecular surface contact area with COV89-22, COV30-14, and COV93-03. The molecular surface contact area for side chain (in gray) and main chain (in white) of each residue of the stem helix peptide in complex with antibody is shown in the stacked bar chart. Where there is no value, the peptide residue is not in contact with the antibody. The molecular surface contact areas were computed with the Molecular Surface package [S4].

(C) The SARS-CoV-2 stem peptide inserts into a hydrophobic groove shaped by the heavy and light chains of COV30-14, COV89-22, and COV93-03. Surfaces of the Fabs are colored in a green gradient by hydrophobicity calculated by Color h (https://pymolwiki.org/index.php/Color_h).

A

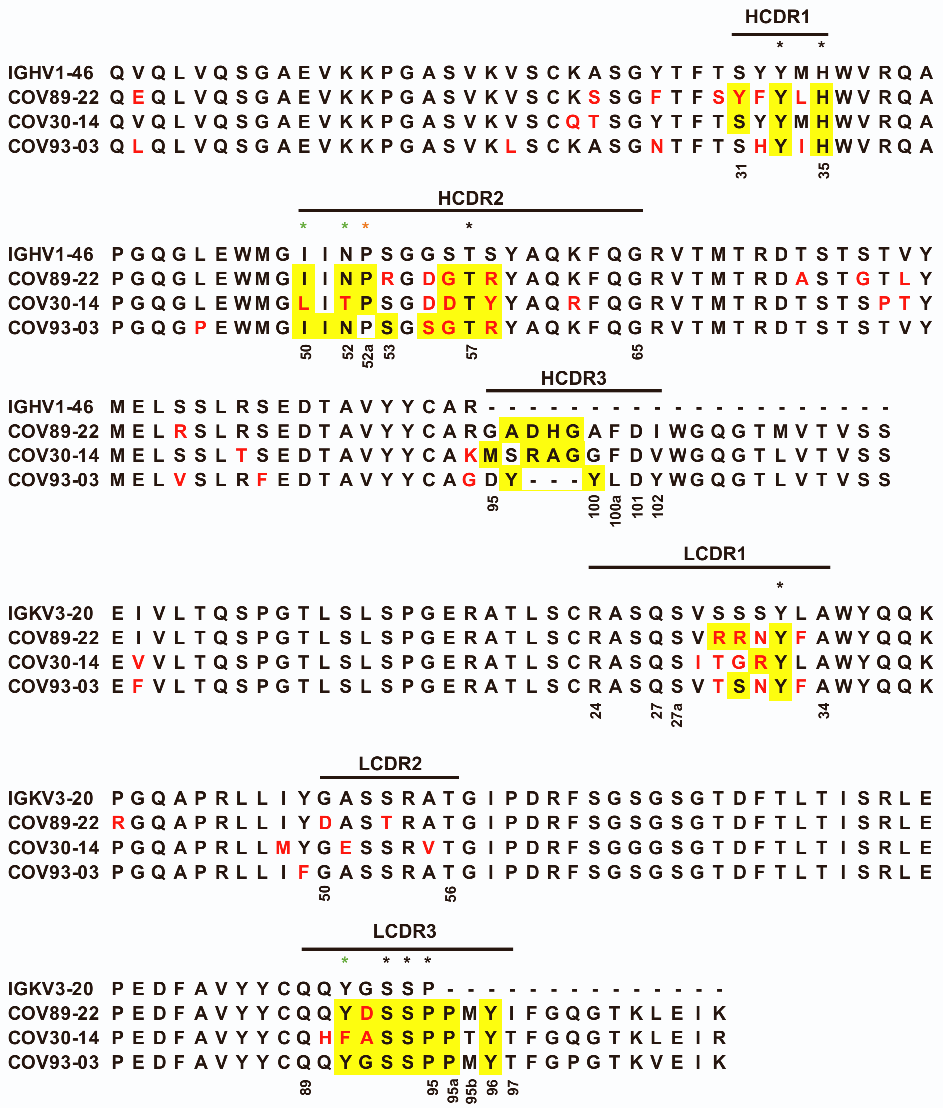

C

FR1 — CDR1 — FR2 — CDR2 — FR3 — CDR3 (positions marked: 33, 35, 50, 52, 57)

Gene	Sequence
IGHV1-2	QVQLVQSGA.EVKKP GASVKVSCKAS GYTF....TGYY MHWVRQAP GQGLEWMGR INPN..SGGT NYAQKFQ.G RVTSTRDTSI STAYMELSRLRS DDTVVYYC AR
IGHV1-3	QVQLVQSGA.EVKKP GASVKVSCKAS GYTF....TSYA MHWVRQAP GQRLEWMGW INAG..NGNT KYSQKFQ.G RVTITRDTSA STAYMELSSLRS EDTAVYYC AR
IGHV1-8	QVQLVQSGA.EVKKP GASVKVSCKAS GYTF....TSYD INWVRQAT GQGLEWMGW MNPN..SGNT GYAQKFQ.G RVTMTRNTSI STAYMELSSLRS EDTAVYYC AR
IGHV1-18	QVQLVQSGA.EVKKP GASVKVSCKAS GYTF....TSYG ISWVRQAP GQGLEWMGW ISAY..NGNT NYAQKLQ.G RVTMTTDTST STAYMELRSLRS DDTAVYYC AR
IGHV1-24	QVQLVQSGA.EVKKP GASVKVSCKVS GYTL....TELS MHWVRQAP GKGLEWMGG FDPE..DGET IYAQKFQ.G RVTMTEDTST DTAYMELSSLRS EDTAVYYC AT
IGHV1-38-4	QVQLVQSWA.EVRKS GASVKVSCSFS GFTI....TSYG IHWVQQSP GQGLEWMGW INPG..NGSP SYAKKFQ.G RFTMTRDMST TTAYTDLSSLTS EDMAVYYY AR
IGHV1-45	QMQLVQSGA.EVKKT GSSVKVSCKAS GYTF....TYRY LHWVRQAP GQALEWMGW ITPF..NGNT NYAQKFQ.D RVTITRDRSM STAYMELSSLRS EDTAMYYC AR
**IGHV1-46**	**QVQLVQSGA.EVKKP GASVKVSCKAS GYTF....TSYY MHWVRQAP GQGLEWMGI INPS..GGST SYAQKFQ.G RVTMTRDTST STVYMELSSLRS EDTAVYYC AR**
IGHV1-58	QMQLVQSGP.EVKKP GTSVKVSCKAS GFTF....TSSA VQWVRQAR GQRLEWIGW IVVG..SGNT NYAQKFQ.E RVTITRDMST STAYMELSSLRS EDTAVYYC AA
IGHV1-68	QVQLGQSEA.EVKKP GASVKVSCKAS GYTF....TCCS LHWLQQAP GQGLERMRW ITLY..NGNT NYAKKFQ.G RVTITRDMSL RTAYIELSSLRS EDSAVYYW AR
IGHV1-69	QVQLVQSGA.EVKKP GSSVKVSCKAS GGTF....SSYA ISWVRQAP GQGLEWMGG IIPI..FGTA NYAQKFQ.G RVTITADEST STAYMELSSLRS EDTAVYYC AR
IGHV1-69-2	EVQLVQSGA.EVKKP GATVKISCKVS GYTF....TDYY MHWVQQAP GKGLEWMGL VDPE..DGET IYAEKFQ.G RVTITADTST DTAYMELSSLRS EDTAVYYC AT
IGHV1-69D	QVQLVQSGA.EVKKP GSSVKVSCKAS GGTF....SSYA ISWVRQAP GQGLEWMGG IIPI..FGTA NYAQKFQ.G RVTITADEST STAYMELSSLRS EDTAVYYC AR
IGHV1-NL1	QVQLLQPGV.QVKKP GSSVKVSC*AS RYTF....TKYF TRWV*QSP GQGHXWMG* INPY..NDNT HYAQTFW.G RVTITSDRSM STAYMELSXLRS EDMVVYYC VR

FR1 — CDR1 — FR2 — CDR2 — FR3 — CDR3 (positions marked: 32, 91, 95)

Gene	Sequence
IGKV3-7	EIVMTQSPPTLSLSP GERVTLSCRAS QSVS.....SSY LTWYQQKP GQAPRLLIY GA.......S TRATSIP.A RFSGSG..SG TDFTLTISSLQP EDFAVYYC QQDHNLP
IGKV3-11	EIVLTQSPATLSLSP GERATLSCRAS QSV......SSY LAWYQQKP GQAPRLLIY DA.......S NRATGIP.A RFSGSG..SG TDFTLTISSLEP EDFAVYYC QQRSNWP
IGKV3-15	EIVMTQSPATLSVSP GERATLSCRAS QSV......SSN LAWYQQKP GQAPRLLIY GA.......S TRATGIP.A RFSGSG..SG TEFTLTISSLQS EDFAVYYC QQYNNWP
**IGKV3-20**	**EIVLTQSPGTLSLSP GERATLSCRAS QSVS.....SSY LAWYQQKP GQAPRLLIY GA.......S SRATGIP.D RFSGSG..SG TDFTLTISRLEP EDFAVYYC QQYGSSP**
IGKV3D-7	EIVMTQSPATLSLSP GERATLSCRAS QSVS.....SSY LSWYQQKP GQAPRLLIY GA.......S TRATGIP.A RFSGSG..SG TDFTLTISSLQP EDFAVYYC QQDYNLP
IGKV3D-11	EIVLTQSPATLSLSP GERATLSCRAS QGV......SSY LAWYQQKP GQAPRLLIY DA.......S NRATGIP.A RFSGSG..PG TDFTLTISSLEP EDFAVYYC QQRSNWH
IGKV3D-15	EIVMTQSPATLSVSP GERATLSCRAS QSV......SSN LAWYQQKP GQAPRLLIY GA.......S TRATGIP.A RFSGSG..SG TEFTLTISSLQS EDFAVYYC QQYNNWP
IGKV3D-20	EIVLTQSPATLSLSP GERATLSCGAS QSVS.....SSY LAWYQQKP GLAPRLLIY DA.......S SRATGIP.D RFSGSG..SG TDFTLTISRLEP EDFAVYYC QQYGSSP

Figure S6. Comparison of COV89-22, COV30-14 and COV93-03 sequences to the IGHV1-46 and IGKV3-20 germlines, related to Figures 4 and 5.

(A) Alignment of the heavy-chain variable domain sequences of COV89-22, COV30-14, and COV93-03 with the human germline IGHV1-46 sequence.

(B) Alignment of the light-chain variable domain sequences of COV89-22, COV30-14, and COV93-03 with the human germline IGKV3-20 sequence. Sequences that correspond to HCDR1, HCDR2, HCDR3, LCDR1, LCDR2, and LCDR3 are indicated. Residues that differ from the germline are in red. Residues interacting with the stem helix peptide are highlighted in yellow. Residue are labeled in Kabat numbering. Black asterisks represent residues identical to germline that interact with the stem helix. The orange asterisk represents the residue identical to germline but interacts in only 2 of 3 antibodies with the stem helix. Green asterisks represent residues that are identical in 2 of 3 antibodies to germline, but still all interact with the stem helix. CDR positions were determined using IgBlast (https://www.ncbi.nlm.nih.gov/igblast/).

(C) Alignment of IGHV1- and IGKV3-encoded amino acid sequences. Amino acids in green are important contact residues for the stem helix and those highlighted in red are unique residues to IGHV1-46 or unique motifs to IGKV3-20/IGKV3D-20.

A

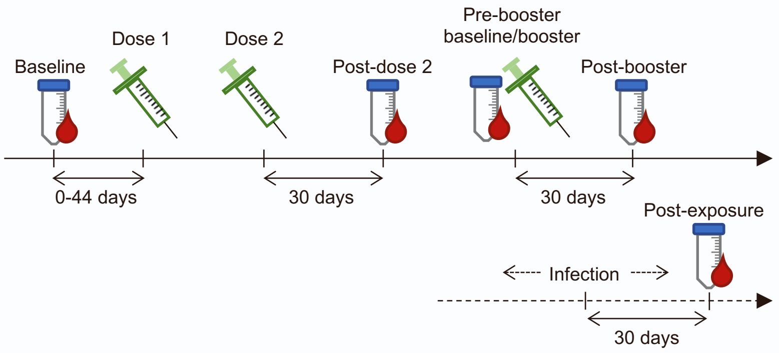

B

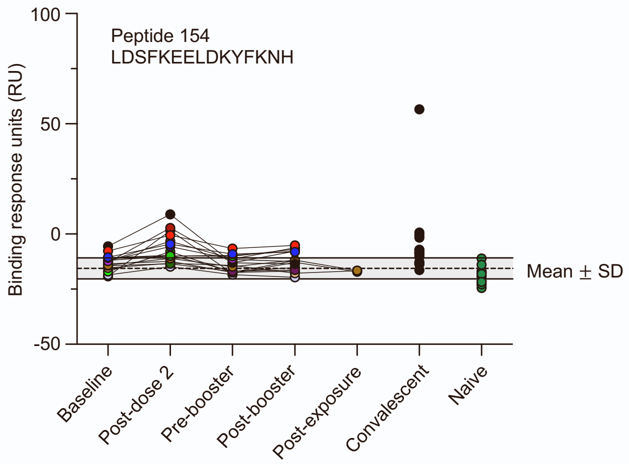

Analysis	*P-value*
Post-dose 2 v Baseline	**<0.001**
Post-dose 2 v Pre-booster	**<0.001**
Post-booster v Pre-booster	1
Post-booster v Baseline	1
Post-dose 2 v Naïve	**0.0182**
Convalescent v Post-dose 2	1
Convalescent v Naïve	**0.0049**

Figure S7. Polyclonal antibody responses to the stem helix after mRNA-1273 vaccination and SARS-CoV-2 infection, related to Figure 2.

(A) Timeline of plasma/serum sample collection and Moderna mRNA-1273 vaccination. Plasma was collected 30 days after documented SARS-CoV-2 infection for three out of 16 volunteers.

(B) Polyclonal IgG reactivity from mRNA-1273-vaccinated (n=16), convalescent unvaccinated (n=16) and COVID-19 naïve (n=13) individuals to peptide 154 from the stem helix region in the S2 subunit. All polyclonal IgG samples were tested at a 100 µg/mL concentration. Background is shown as mean ± SD of donors in the baseline and COVID-19-naïve groups. Pairwise comparisons were made from a nested, mixed-model ANOVA with Bonferroni-adjusted P-values.

Table S1. X-ray data collection and refinement statistics, related to Figures 4 and 5.

**Data collection**	COV89-22+ peptide 154	COV30-14+ peptide 154	COV93-03+ peptide 155
Beamline	SSRL12-1	APS 23-ID-B	APS 23-ID-B
Wavelength (Å)	0.9795	1.033	1.033
Space group	$P2_1$	$P2_12_12_1$	$P2_12_12_1$
Unit cell parameters			
a, b, c (Å)	42.08 76.91 154.38	75.36 83.20 170.38	65.86 65.97 197.65
α, β, γ (°)	90 93.8 90	90 90 90	90 90 90
Resolution (Å) [a]	50-1.6 (1.66-1.60)	50-1.5 (1.55-1.50)	50-1.75 (1.81-1.75)
Unique reflections [a]	99,459 (2,721)	137,051 (5,040)	80,275 (4,909)
Redundancy [a]	6.0 (3.7)	11.7 (6.0)	9.8 (3.4)
Completeness (%) [a]	77.3 (17.7)	80.5 (27.3)	92.6 (52.6)
$<I/\sigma_I>$ [a]	21.3 (0.9)	40.5 (0.8)	22.8 (0.9)
R_{sym}[b] (%) [a]	8.4 (94)	6.9 (>100)	13.8 (65)
R_{pim}[b] (%) [a]	3.6 (55)	2.0 (46)	4.3 (40)
$CC_{1/2}$[c] (%) [a]	99.4 (52.6)	99.9 (51.0)	99.1 (52.0)
**Refinement statistics**			
Resolution (Å)	42.71-1.60	46.9-1.5	46.6-1.75
Reflections (work)	99,449	137,013	80,269
Reflections (test)	4972	6742	4017
R_{cryst}[d] / R_{free}[e] (%)	18.5/21.0	19.8/22.1	19.4/22.3
No. of copies in ASU	2	2	2
No. of atoms	8002	7825	7463
Fab	6694	6487	6473
Peptide	270	270	215
Solvent	1038	1068	775
Average *B*-values (Å^2)	27	31	32
Fab	25	30	31
Peptide	36	29	35
Solvent	35	37	39
Wilson *B*-value (Å^2)	20	21	26
**RMSD from ideal geometry**			
Bond length (Å)	0.005	0.006	0.004
Bond angle (°)	0.79	0.86	0.76
**Ramachandran statistics (%)**[f]			
Favored	98.5	98.4	98.3
Outliers	0.00	0.00	0.12
**PDB code**	8DTX	8DTR	8DTT

[a] Numbers in parentheses refer to the highest resolution shell.

[b] $R_{sym} = \Sigma_{hkl} \Sigma_i | I_{hkl,i} - <I_{hkl}> | / \Sigma_{hkl} \Sigma_i I_{hkl,i}$ and $R_{pim} = \Sigma_{hkl} (1/(n-1))^{1/2} \Sigma_i | I_{hkl,i} - <I_{hkl}> | / \Sigma_{hkl} \Sigma_i I_{hkl,i}$, where $I_{hkl,i}$ is the scaled intensity of the ith measurement of reflection h, k, l, $<I_{hkl}>$ is the average intensity for that reflection, and *n* is the redundancy.

[c] $CC_{1/2}$ = Pearson correlation coefficient between two random half datasets.

[d] $R_{cryst} = \Sigma_{hkl} | F_o - F_c | / \Sigma_{hkl} | F_o | \times 100$, where F_o and F_c are the observed and calculated structure factors, respectively.

[e] R_{free} was calculated as for R_{cryst}, but on a test set comprising 5% of the data excluded from refinement.

[f] From MolProbity [S5].

Table S2. Coronavirus neutralization by IGHV1-46/IGKV3-20 stem helix-specific mAbs ($Assay_{NIH}$), related to Figure 6.

mAb	Pseudovirus neutralization (NT_{50}) (µg/mL)			
	SARS-CoV-2 (WA-1)	SARS-CoV	MERS-CoV	NL63
**COV30-14**	35.9	6.54	0.228	>100
**COV72-37**	33.2	7.10	0.863	>100
**COV89-22**	13.8	1.95	1.24	>100

Table S3. Primers for generation of SARS-CoV-2 spike mutants, related to STAR Methods.

	Forward primer	Reverse primer
**F1148A**	gaactggactccGCcaaggaggaactggac	tcctccttggcggagtccagttcaggttggag
**L1152A**	ccttcaaggaggaaGCTgacaaatacttcaagaaccac	cttgaagtatttgtcAGCttcctccttgaaggagtccagttc
**F1156A**	actggacaaatacGCcaagaaccacaccagc	tggtgtggttcttgGCgtatttgtccagttcctc
**F1148A/ L1152A/ F1156A**	GCcaaggaggaaGCTgacaaatacGCcaagaaccacac	gtatttgtcAGCttcctccttgGCggagtccagttcaggttgga
**End**	ctggacaaggtggaggctgag	caagcttccatggctcgagtcacttacaacaggagccacagga

[a]Capitalized nucleotides are those that differ from the wild-type sequence and were used to generate the mutants.

Supplementary References

S1. Wang, L.T., Pereira, L.S., Flores-Garcia, Y., O'Connor, J., Flynn, B.J., Schon, A., Hurlburt, N.K., Dillon, M., Yang, A.S.P., Fabra-Garcia, A., et al. (2020). A potent anti-malarial human monoclonal antibody targets circumsporozoite protein minor repeats and neutralizes sporozoites in the liver. Immunity *53*, 733-744.e8. 10.1016/j.immuni.2020.08.014.

S2. Kepler, T.B. (2013). Reconstructing a B-cell clonal lineage. I. Statistical inference of unobserved ancestors. F1000Res *2*, 103. 10.12688/f1000research.2-103.v1.

S3. Krissinel, E., and Henrick, K. (2007). Inference of macromolecular assemblies from crystalline state. J Mol Biol *372*, 774-797. 10.1016/j.jmb.2007.05.022.

S4. Connolly, M.L. (1983). Analytical molecular surface calculation. J Appl Crystallogr *16*, 548-558. 10.1107/S0021889883010985.

S5. Chen, V.B., Arendall, W.B., 3rd, Headd, J.J., Keedy, D.A., Immormino, R.M., Kapral, G.J., Murray, L.W., Richardson, J.S., and Richardson, D.C. (2010). MolProbity: all-atom structure validation for macromolecular crystallography. Acta Crystallogr D Biol Crystallogr *66*, 12-21. 10.1107/S0907444909042073.
